# Cytotoxic Potential of a-Azepano- and 3-Amino-3,4-SeCo-Triterpenoids

**DOI:** 10.3390/ijms22041714

**Published:** 2021-02-08

**Authors:** Oxana Kazakova, Irina Smirnova, Elena Tret’yakova, René Csuk, Sophie Hoenke, Lucie Fischer

**Affiliations:** 1 Ufa Institute of Chemistry of the Ufa Federal Research Centre of the Russian Academy of Sciences, 71, pr. Oktyabrya, 450054 Ufa, Russia; si8081@yandex.ru (I.S.); tretya3@yandex.ru (E.T.); 2 Organic Chemistry, Martin-Luther-University Halle-Wittenberg, Kurt-Mothes-Str. 2, D-06120 Halle (Saale), Germany; sophie.hoenke@chemie.uni-halle.de (S.H.); lucie.fischer2018@gmx.de (L.F.)

**Keywords:** triterpenoids, betulin, oleanolic acid, ursolic acid, glycyrrhetinic acid, azepane, cytotoxicity, cell cycle analysis, NCI-60 cell panel, apoptosis

## Abstract

Semi-synthetic triterpenoids, holding an amino substituted seven-membered A-ring (azepano-ring), which could be synthesized from triterpenic oximes through a Beckmann type rearrangement followed by a reduction of lactame fragment, are considered to be novel promising agents exhibiting anti-microbial, alpha-glucosidase, and butyrylcholinesterase inhibitory activities. In this study, in an attempt to develop new antitumor candidates, a series of A-ring azepano- and 3-amino-3,4-seco-derivatives of betulin, oleanolic, ursolic, and glycyrrhetinic acids were evaluated for their cytotoxic activity against five human cancer cell lines and non-malignant mouse fibroblasts by means of a colorimetric sulforhodamine assay. Azepanoallobetulinic acid amide derivative **11** was the most cytotoxic compound of this series but showed little selectivity between the different human tumor cell lines. Flow cytometry experiments showed compound **11** to act mainly by apoptosis (44.3%) and late apoptosis (21.4%). The compounds were further screened at the National Cancer Institute towards a panel of 60 cancer cell lines. It was found that compounds **3**, **4**, **7**, **8**, **9**, **11**, **15**, **16**, **19**, and **20** showed growth inhibitory (GI_50_) against the most sensitive cell lines at submicromolar concentrations (0.20–0.94 μM), and their cytotoxic activity (LC_50_) was also high (1–6 μM). Derivatives **3**, **8**, **11**, **15**, and **16** demonstrated a certain selectivity profile at GI_50_ level from 5.16 to 9.56 towards K-562, CCRF-CEM, HL-60(TB), and RPMI-8226 (Leukemia), HT29 (Colon cancer), and OVCAR-4 (Ovarian cancer) cell lines. Selectivity indexes of azepanoerythrodiol **3** at TGI level ranged from 5.93 (CNS cancer cell lines SF-539, SNB-19 and SNB-75) to 14.89 for HCT-116 (colon cancer) with SI 9.56 at GI_50_ level for the leukemia cell line K-562. The present study highlighted the importance of A-azepano-ring in the triterpenic core for the development of novel antitumor agents, and a future aim to increase the selectivity profile will thus lie in the area of modifications of azepano-triterpenic acids at their carboxyl group.

## 1. Introduction

Demographic changes such as the growth of the world population combined with an increase in life expectancy led to an increasing number of patients suffering from oncological diseases. Thus, cancer is a one of the leading causes of death worldwide, and the number of new cases of diseases caused by cancer is expected to rise by about 70% over the next two decades [1]. Therefore, there is an urgent need of novel antitumor agents acting by novel modes of action. A promising strategy is to generate new antitumor agents from natural products. This seems particularly interesting for two reasons: On the one hand, pharmaceuticals derived from natural substances have already proven to be very effective in many cases [2], and on the other hand, this strategy results in sustainability in the sense of a green, ecologically oriented chemistry. 

Natural products remain an excellent source of drugs; for example, from 1980s to date, nearly 50% of all drugs are of natural product origin. Pentacyclic triterpenoids, which are naturally occurring secondary metabolites widespread in plants, marine invertebrates, and fungi, were recently reconsidered as model compounds for the development of innovative anticancer agents. Their activity against proteins involved in tumor development enhances the opportunity to exploit these compounds as new multi-target therapeutic agents [3].

Over the past years, a large number of triterpenoids have been chemically modified in order to improve their bioactivity and bioavailability and to enhance their protective and/or therapeutic effects. Many studies reported triterpenoids including betulin and betulinic acid, oleanolic, ursolic, boswellic, maslinic, and glycyrrhetinic acids to exhibit a broad spectrum of biological activities, such as anti-inflammatory, anticancer, antimicrobial, or antidiabetic [4,5,6,7,8,9].

As far as cytotoxic properties are concerned, the presence of further functional groups seems to be necessary for pentacyclic triterpene carboxylic acid derivatives to achieve sufficiently low EC_50_ values. Thus, the presence of an amino substituent in ring A leads to cytotoxic but unselective compounds [10]. Simple carboxylic acid amides are often cytotoxic in micro polar concentrations and highly selective [11,12,13,14], whereas rhodamine B conjugates can reach EC_50_ values in the nano-molar concentration range but hold the risk of being highly cytotoxic for all types of cells [15,16].

Among these derivatives, triterpenoids, which could be synthesized from triterpenic oximes through a Beckmann type rearrangement followed by a subsequent reduction reaction holding an A-azepane ring or a 3-amino-3,4-seco-4(24)-en-fragment are a group of new and promising modifications exhibiting anti-microbial [17,18,19,20], anticancer [21,22,23,24,25,26], alpha-glucosidase [27,28] as well as butyrylcholinesterase [29] inhibitory activities.

## 2. Results and Discussion

### 2.1. Chemistry

**Figure 1:**
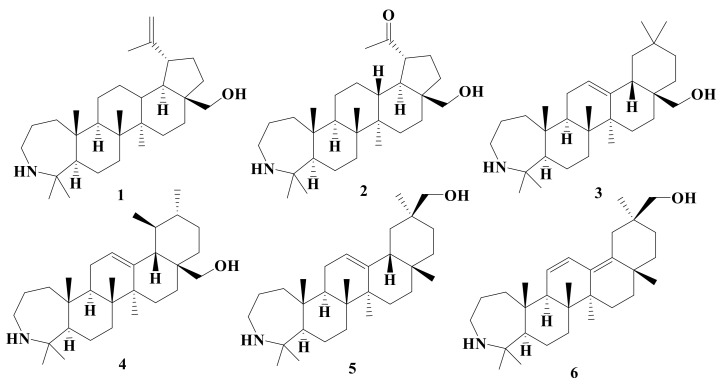
Azepanotriterpenic alcohols: Azepanobetulin **1**, azepanomessagenin **2**, azepanoerythrodiol **3**, azepanouvaol **4**, azepano-glycyrrhetols **5** and **6**.

**Figure 2:**
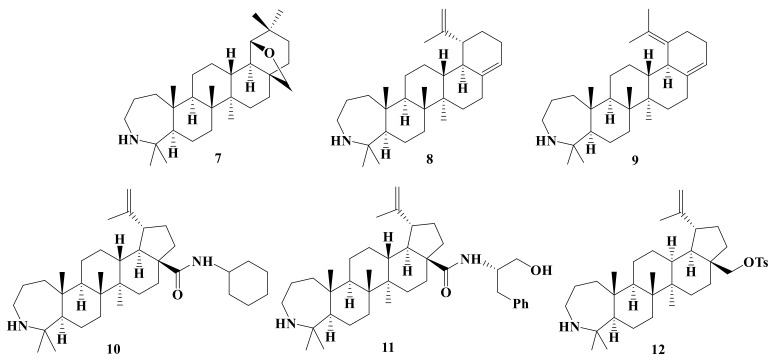
E-ring and C28 azepanobetulin derivatives: Azepanoallobetulin **7**, *abeo*-lupanes **8** and **9**, amides **10** and **11**, tosylate **12.**

**Figure 3:**
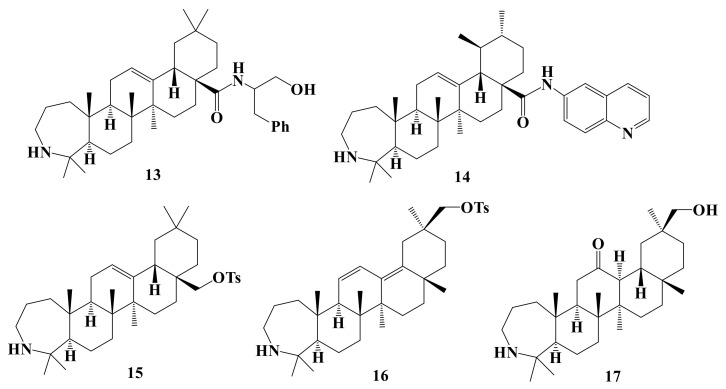
Azepanoerythrodiol, azepanouvaol, and azepano-glycyrrhetol derivatives: Amides **13** and **14**, tosylates **15** and **16**, 12-ketone **17**.

**Figure 4:**
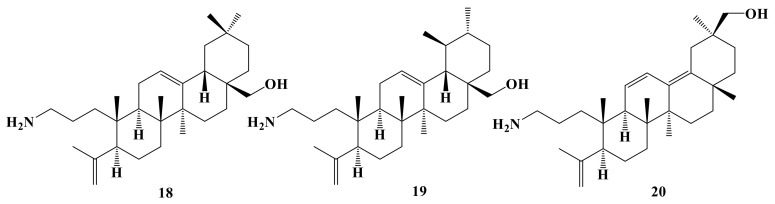
3-Amino-3,4-seco-4(23)-en triterpenic derivatives of erythrodiol **18**, uvaol **19** and glycyrrhetol-dien **20**.

In this study, a total of 20 A-ring azepano- **1–17** and 3-amino-3,4-seco- **18–20** triterpenoids were synthesized from betulin, oleanolic, ursolic, and glycyrrhetinic acids according to methods described earlier [17,18,19,21,22,26,29]. Their structures are presented at Figure 1, Figure 2, Figure 3 and Figure 4.

### 2.2. Biological Evaluation

#### 2.2.1. Cytotoxic Activities on Tumor Cell Cultures (A375, HT29, MCF-7, A2780, FaDu, and NIH 3T3)

The cytotoxicity of compounds **1–20** was evaluated by means of a colorimetric sulforhodamine B (SRB) assay. For all compounds, the EC_50_ values were determined for the human tumor cell lines A375 (malignant melanoma), HT29 (colorectal carcinoma), MCF-7 (breast carcinoma), A2780 (ovarian carcinoma), FaDu (hypopharyngeal carcinoma), and NIH 3T3 (non-malignant mouse fibroblasts). The results are compiled in the Table 1.

The results from the SRB assays showed all compounds of good cytotoxicity with EC_50_ values ranging between 0.88 µM (compound **11** for FaDu cells) and 7.92 µM (compound **2** for A375 cells). Compound **11** was the most cytotoxic of this series, but this compound showed little selectivity between the different human tumor cell lines as well as to non-malignant fibroblasts. Compound **6**, however, was most cytotoxic for A2780 ovarian cancer cells (EC_50_ = 3.93 µM) but significantly less cytotoxic for non-malignant fibroblasts (NIH 3T3, 11.68 µM) thus resulting in a selectivity factor S (EC_50 (NIH 3T3_)/EC_50 (A2780_) of approximately 3 (Table 2). 

#### 2.2.2. Cell Cycle Analysis

**Figure 5:**
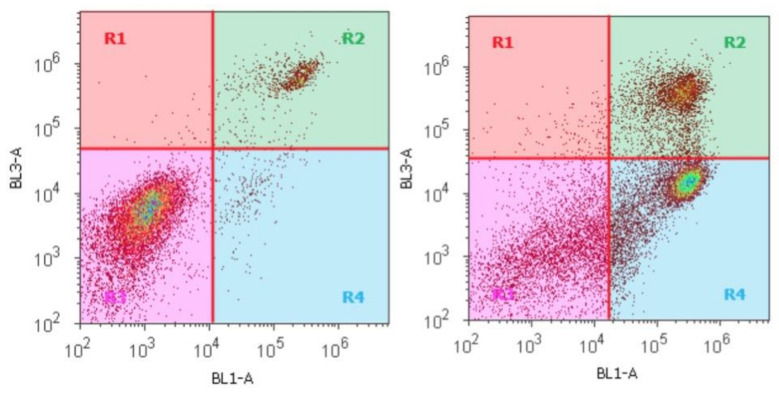
Annexin V/PI flow cytometry of **11** employing A375 cells (48 h of incubation, 2 × EC_50_ concentration); control experiment (left), incubation with **11** (right); R1 necrotic cells, R2 late apoptotic cells, R3 viable cells, and R4 apoptotic cells.

Due to its good cytotoxic properties, compound **11** was subjected to flow cytometric measurements (Annexin V/PI assay). Thereby, A375 cells were treated with 2 × EC_50_ concentrations of **11** for 48 h, and the results from these experiments are depicted in Figure 5.

Thereby, the BL1-A signal corresponds to the FITC signal for annexin V (x-axis) while PI is detected at BL3-A (y-axis). Thus, cells in R1 correspond to necrotic cells, those in R2 to late apoptosis, cells in R3 are viable cells, and cells in R4 have died from apoptosis. Thus, from the 48 h incubation of **11**, 44.3% of the A375 cells have died by apoptosis and 21.4% by late apoptosis. The number of necrotic cells remained small (1.1%).

#### 2.2.3. NCI-60 Anticancer Drug Screening

Taking into account the results of cytotoxicity of triterpenoids **1–20** against the five human cancer cell lines and non-malignant mouse fibroblasts, they were subjected to the NCI-60 Anticancer Drug Screening. Compounds **1–9**, **11–17**, **19,** and **20** were selected by National Cancer Institute (NCI, Bethesda, Rockville, MD, USA) Developmental Therapeutic Program (DTP) and tested at one dose assay (10^−5^ M) towards a panel of approximately 60 cancer cell lines representing different cancer types: Leukemia, melanoma, lung, colon, CNS, ovarian, renal, prostate, and breast cancers. Primary anticancer assays were performed according to the NCI protocol as described elsewhere (see e.g., http://dtp.nci.nih.gov (accessed on 16 October 2019)) [30,31,32,33,34]. The compounds were added at a single concentration and the cell cultures were incubated for 48 h. The end point determinations were made with a protein binding dye, sulforhodamine B (SRB). The results for each compound are reported as the percent growth (GP %) of treated cells compared to untreated control cells (negative numbers indicate cell kill). The range of percent growth shows the lowest and the highest percent growth found among the different cancer cell lines (Table 3 and Figures S1–S18 Supporting Material).

The anticancer activity results showed that the cytotoxic effect of the studied azepano derivatives was directly dependent on the type of triterpenic core. So, the azepanomessagenin **2** did not show antiproliferative activity, while the azepanobetulin **1** and azepanoerythrodiol **3** were effective for 28 and 30 cancer cell lines. The anticancer activity of the azepano-glycyrretols was strongly influenced by the presence of an additional double bond in the triterpene core—thus, azepano-glycyrretol **5** was active only towards 8 human tumor cell lines, while diene **6** demonstrated a pronounced antiproliferative effect against the whole NCI-60 cancer cell line panel. Azepanouvaol **4** was also highly active against 57 cell lines. The transformation of azepanobetulin **1** into azepanoallobetulin **7** led to a significant increase in activity against 52 cancer cell lines, whereas the anticancer effect of *abeo*-lupanes **8** and **9** influenced by the position of the double bond. Thus, abeo-lupane **8** effectively acted against 55 cell lines, while its regio-isomer **9** was active only towards 36 lines. Modification at the C28 position of azepanobetulin derivatives increased the activity of the initial compounds—the introduction of a tosyl fragment (compound **12**) led to activity against 39 cancer cell lines, and the amide fragment (compound **11**) was effective against the all NCI-60 cancer cell line panel. At the same time, the introduction of amide fragments into the C28 position of the azepanotriterpenic alcohols **4** and **5** led to a general decrease of anticancer effect-amide **13** was active only towards 6 tumor cell lines, and amide **14**-against 45 cell lines. Replacement of the hydroxyl group to tosyl as in compounds **15** and **16** also led to a decrease in antiproliferative activity-these compounds inhibited 22 and 40 cell lines, respectively. Oxidation of the double bond to a 12-oxo group (compound **17**) led to a complete loss of cytotoxic activity, whereas 3-amino-3,4-seco-4(23)-en triterpenic derivatives of uvaol **19** and glycyrrhetol **20** showed an activity against 45 and 52 cancer cell lines, respectively.

Finally, 15 A-azepano- **1**, **3**, **4**, **6**, **7–9**, **11**, **12**, **14–16,** and 3-amino-3,4-seco-triterpenoids **19**, **20** were selected in an advanced assay against a panel of approximately sixty tumor cell lines at 10-fold dilutions of five concentrations (100 µM, 10 µM, 1 µM, 0.1 µM, and 0.01 µM). The percentage of growth was evaluated spectrophotometrically versus controls not treated with the test agents after 48-h exposure and using SRB protein assay to estimate cell viability or growth. Three antitumor activity dose-response parameters were calculated for each cell line: GI_50_—molar concentration of the compound that inhibits 50% net cell growth; TGI—molar concentration of the compound leading to the total inhibition; and LC_50_—molar concentration of the compound leading to 50% net cell death (presented in negative logarithm). Furthermore, mean graph midpoints (MG_MID) were calculated for each of the parameters, giving an average activity parameter over all cell lines for the tested compound. For the MG_MID calculation, insensitive cell lines were included with the highest concentration tested (see the Supporting Material Figures S19–S32 and Tables S1–S3).

Thus, all compounds exhibited significant antiproliferative effect towards human cancer cell lines, and among them, the highest cytotoxic activity in five-dose testing mode screening was observed for compounds **3**, **4**, **7**, **8**, **9**, **11**, **15**, **16**, **19**, and **20** with growth inhibitory (GI_50_) against the most sensitive cell lines at submicromolar concentrations (0.20–0.94 μM). Cytotoxic activity (LC_50_) of these compounds against the most sensitive cancer cell lines was also high (1–6 μM). The compounds **3** and **19** showed a broad spectrum of growth inhibition activity (GI_50_ < 10 μM) against all human tumor cells with average GI_50_/TGI/LC_50_ values of 3.83/15.49/39.56 µM (**3**), and 1.42/3.89/16.97 µM (**20**), respectively. Compounds **1**, **4**, **7**, **8**, **9**, **11**, **12**, **14**, **16**, and **20** inhibited the growth of all tested cancer cell lines and showed inhibition activity (GI_50_ < 3 μM) against all human tumor cell lines with average GI_50_/TGI/LC_50_ values of 1.70/3.34/17.48 μM (**1**), 1.65/3.31/7.64 µM (**4**), 1.68/3.64/17.17 µM (**7**), 1.73/3.53/16.89 µM (**8**), 1.75/3.35/6.43 µM (**9**), 1.29/2.64/5.62 µM (**11**), 1.77/3.43/5.84 µM (**12**), 1.72/3.35/6.52 µM (**14**), 1.72/3.52/19.43 µM (**16**), and 1.66/3.36/17.30 µM (**20**), respectively. Compounds **6** and **15** demonstrated growth inhibition activity (GI_50_ < 15 μM) towards all cancer cell lines with average GI_50_/TGI/LC_50_ values of 4.21/13.17/41.94 µM (**6**) and 1.99/3.80/29.02 µM (**15**) (Tables S1–S3 Supporting Material). Mean GI_50_ values for these compounds in comparison with standard anticancer agent’s doxorubicin and 5-fluorouracil [35] are given at Table S3 Supporting Material.

The selectivity index (SI) was calculated by dividing the full panel MG_MID_60_ (μM) of the compounds **1**, **3**, **4**, **6–9**, **11**, **12**, **14–16**, **19,** and **20** by their individual subpanel MG_MID of the cell line (μM) and is to be considered as a measure of the compounds’ selectivity (Table 4). Ratios between 3 and 6 mean moderate selectivity, ratios greater than 6 indicate high selectivity towards the corresponding cell line, while compounds not meeting either of these criteria are rated nonselective [36]. In this context, the compounds **4**, **6–9**, **12**, **14**, and **20** in the present study were found to be nonselective at all the GI_50_, TGI, and LC_50_ levels (selectivity indexes 0.57–1.59, 0.66–1.52 and 0.17–2.95, respectively). Based on the selectivity ratio, compound **11** in the study was found to be moderate selective for a growth inhibition regarding the leukemia subpanel with a selectivity ratio at GI_50_ level of 3.79, otherwise, it was found lower selective against other cell panels. Compound **3** was moderately selective at the TGI and LC_50_ levels towards colon, CNS, prostate cancer, and melanoma (selectivity indexes 3.59–4.69 and 3.18–4.63, respectively), as well as compounds **1**, **15**, **16**, and **19** at the LC_50_ level (selectivity indexes 3.09–4.81). 

Furthermore, derivatives **3**, **8**, **11**, **15**, and **16** demonstrated a certain selectivity profile towards some individual cell lines at GI_50_ and TGI levels. GI_50_ level was from 5.16 to 6.45 for HL (60)-TB, K-562, CCRF-CEM, RPMI-8226 (leukemia), HT29 (colon cancer), and OVCAR-4 (ovarian cancer). At the same time, TGI level of moderate selectivity was observed for compound **11** towards leukemia cell line HL(60)-TB (SI = 5.74). Selectivity indexes of compound **3** at TGI level was 5.93–14.89 for HCT-116 (colon cancer), SF-539, SNB-19, and SNB-75 (CNS cancer). However, for compound **3** high selectivity at GI_50_ level was observed only for leukemia cell line K-562 (SI = 9.56) (Table 5).

A raw comparison of the activities of studied compounds with respect to the activity reported for the standard drugs doxorubicin and 5-fluorouracil, used by NCI as control [35] reflects that the activity displayed for these compounds was lower than for doxorubicin except for colon cancer HCT-15 and ovarian cancer NCI/ADR-RES (compound **11**). Comparison of the compounds **1**, **3**, **4**, **6**, **7–9**, **11**, **12**, **14–16**, **19**, and **20** activities with 5-fluorouracil showed that the studied compounds were more active against cell lines of leukemia CCRF-CEM, HL(60)-TB (exception compound **4**), K-562; NSCL cancer HOP-92, NCI-H226, and NCI-H522; CNS cancer SNB-75 (exception compounds **3** and **6**); melanoma SK-MEL-2 and UACC-257; ovarian cancer OVCAR-4 (exception **3** and **6**), OVCAR-5, and SK-OV-3; renal cancer RXF 393 (exception **3**); prostate cancer PC-3 (exception **1**); breast cancer MDA-MB-31/ATCC, HS 578T, BT-549, and T-47D. Furthermore, compound **11** also showed the best inhibition of colon cancer SW-620, CNS cancer SF-268, melanoma M14, ovarian cancer OVCAR-8, renal cancer 786-0 cell lines; compound **15**—renal cancer TK-10; compound **19**—CNS cancer SF-268, melanoma M14, SK-MEL-28, ovarian cancer IGROV1, OVCAR-8, renal cancer TK-10; compound **20**—CNS cancer SF-268 and renal cancer TK-10 (Table S1 Supporting Material). 

## 3. Materials and Methods

### 3.1. Pharmacological Studies

#### 3.1.1. SRB Assay

In short, exponentially growing cells were seeded into 96-well plates on day 0 at the appropriate cell densities to prevent confluence of the cells during the period of experiment. After 24 h, the cells were treated with serial dilutions of the compounds (0–30 µM) for 72 h. The final concentration of DMSO never exceeded 0.5%, which was non-toxic to the cells. After a 72 h treatment, the supernatant medium from the 96-well plates was discarded, and the cells were fixed with 10% TCA. For a thorough fixation, the plates were allowed to rest at 4 °C. After fixation, the cells were washed in a strip washer. The washing was done four times with water using alternate dispensing and aspiration procedures. The plates were then dyed with 100 µL of 0.4% SRB for about 20 min. After dying, the plates were washed with 1% acetic acid to remove the excess of the dye and allowed to air-dry overnight. Tris base solution (200 µL, 10 mM) was added to each well and absorbance was measured at λ = 570 nm (using a 96 well plate reader, Tecan Spectra, Crailsheim, Germany). The percentages of surviving cells relative to untreated controls were determined. The EC_50_ values were averaged from three independent experiments performed each in triplicate calculated from semi logarithmic dose response curves applying a non-linear 4P Hills-slope equation (GraphPad Prism5; the variables top and bottom were set to 100 and 0, respectively). 

#### 3.1.2. Annexin V/PI Assay

Approximately 600,000 cells (A375) were seeded in cell culture flasks and were allowed to grow for 1 day. After removing of the medium, the substance loaded medium was added, and the flasks were incubated for 48 h. All cells were harvested, centrifuged (1200 rpm, 5 min), and washed twice (PBS (*w*/*w*)). Approximately 100,000 cells were washed with annexin V bounding buffer (BD Biosciences^®^) and treated with a propidium iodide solution (3 μL, 1 mg/mL) and annexin V (5 μL, BD Biosciences^®^) for 15 min at room temperature in the dark. After adding annexin V bounding buffer (400 μL) the suspension was submitted to a FACS measurement. Calculation was performed as suggested from the supplier (BD Biosciences^®^).

#### 3.1.3. In Vitro Cancer Screen in NCI, USA

The screening is a two-stage process, beginning with the evaluation of all compounds against the 60 cell lines at a single dose of 10^−5^ M. Compounds that exhibit significant growth inhibition are evaluated against the 60-cell panel at five concentration levels. The human tumor cell lines of the cancer-screening panel are grown in RPMI 1640 medium containing 5% fetal bovine serum and 2 mM *L*-glutamine. For a typical screening experiment, cells are inoculated into 96-well micro titer plates in 100 mL at plating densities ranging from 5000 to 40,000 cells/well depending on the doubling time of individual cell lines. After cell inoculation, the micro titer plates are incubated at 37 °C, 5% CO, 95% air, and 100% relative humidity for 24 h prior to addition of experimental drugs. After 24 h, two plates of each cell line are fixed in situ with TCA, to represent a measurement of the cell population for each cell line at the time of drug addition (Tz). Experimental drugs are solubilized in dimethylsulfoxide at 400-fold the desired final maximum test concentration and stored frozen prior to use. At the time of drug addition, an aliquot of frozen concentrate is dissolved and diluted to twice the desired final maximum test concentration with complete medium containing 50 mg/mL gentamicin. Additional four, 10-fold, or ½ log serial dilutions are made to provide a total of five drug concentrations plus control. Aliquots of 100 mL of these different drug dilutions are added to the appropriate micro titer wells already containing 100 mL of medium, resulting in the required final drug concentrations. Following drug addition, the plates are incubated for an additional 48 h at 37 °C, 5% CO_2_, 95% air, and 100% relative humidity. For adherent cells, the assay is terminated by the addition of cold TCA. Cells are fixed in situ by the gentle addition of 50 mL of cold 50% TCA (final concentration, 10% TCA) and incubated for 60 min at 4 °C. The supernatant is discarded, and the plates are washed five times with tap water and air dried. Sulforhodamine B (SRB) solution (100 mL) at 0.4% in 1% acetic acid is added to each well, and plates are incubated for 10 min at room temperature. After staining, unbound dye is removed by washing five times with 1% acetic acid and the plates are air dried. Bound stain is subsequently solubilized with 10 mM Trizma base, and the absorbance is read on an automated plate reader at a wavelength of 515 nM. For suspension cells, the methodology is the same except that the assay is terminated by fixing settled cells at the bottom of the wells by gently adding 50 mL of 80% TCA (final concentration, 16% TCA). Using the seven absorbance measurements [time zero (Tz), control growth (C), and test growth in the presence of drug at the five concentration levels (Ti)], the percentage growth is calculated at each of the drug concentrations levels. Percentage growth inhibition is calculated as:

[(Ti_Tz)/(C_Tz)]_100 for concentrations for which Ti_Tz
[(Ti_Tz)/Tz]_100 for concentrations for which Ti < Tz

Three dose response parameters are calculated for each experimental agent. Growth inhibition of 50% (GI_50_) is calculated from [(Ti_Tz)/(C_Tz)]_100 ¼ 50, which is the drug concentration resulting in a 50% reduction in the net protein increase (as measured by SRB staining) in control cells during the drug incubation. The drug concentration resulting in total growth inhibition (TGI) is calculated from (Ti ¼ Tz). The LC_50_ (concentration of drug resulting in a 50% reduction in the measured protein at the end of the drug treatment as compared to that at the beginning) indicating a net loss of cells following treatment is calculated from

[(Ti_Tz)/Tz]_100¼_50

Values are calculated for each of these three parameters if the level of activity is reached; however, if the effect is not reached or is exceeded, the value for that parameter is expressed as greater or less than the maximum or minimum concentration tested [30,31,32,33,34].

## 4. Conclusions

In summary, a series of A-ring azepano- and 3,4-seco-derivatives of betulin, oleanolic, ursolic, and glycyrrhetinic acids were evaluated as cytotoxic agents. Azepanoallobetulinic acid amide derivative **11** was the most cytotoxic compound of this series but showed little selectivity between the different human tumor cell lines, while azepano-glycyrrhetol-diene **6** was most cytotoxic for A2780 ovarian cancer cells (EC_50_ = 3.93 µM) but significantly less cytotoxic for non-malignant fibroblasts (NIH 3T3, 11.68 µM) thus resulting in a selectivity factor S (EC_50 (NIH 3T3_)/EC_50 (A2780_) of approximately 3. Flow cytometry experiments showed compound **11** to act mainly by apoptosis (44.3%) and late apoptosis (21.4%). The further screening toward the NCI-60 cancer cell panel showed submicromolar level of GI_50_ for compounds **3**, **4**, **7**, **8**, **9**, **11**, **15**, **16**, **19**, and **20** (range from 0.20 to 0.94 μM) against the most sensitive cell lines, while LC_50_ was also high (1–6 μM). Derivatives **3**, **8**, **11**, **15**, and **16** demonstrated a certain selectivity profile from 5.16 to 9.56 toward individual cell lines of Leukemia, Colon cancer and Ovarian cancer. Selectivity of a leader compound azepanoerythrodiol **3** at TGI level ranged from 5.93 for SF-539, SNB-19, and SNB-75 (CNS cancer) to 14.89 for HCT-116 (colon cancer). Thus, A-ring-azepano triterpenoids are interesting starting structures for the synthesis of biologically active molecules, especially of cytotoxic agents.

## Figures and Tables

**Table 1:** Cytotoxicity * of compounds **1**–**20** against the human cancer cell lines A375, HT29, MCF-7, A2780, FaDu, and non-malignant mouse fibroblasts NIH 3T3.

Compound	EC_50_*					
	A375	HT29	MCF-7	A2780	FaDu	NIH 3T3
1	2.52 ± 0.2	1.63 ± 0.3	1.91 ± 0.1	2.39 ± 0.2	2.32 ± 0.3	1.60 ± 0.4
2	7.82 ± 0.3	6.32 ± 0.4	5.78 ± 0.7	7.71 ± 0.4	6.21 ± 1.2	8.03 ± 1.4
3	n.s.	n.s.	n.s.	n.s.	n.s.	n.s.
4	2.4 ± 0.2	1.71 ± 0.3	2.30 ± 0.2	2.63 ± 0.1	1.69 ± 0.4	2.77 ± 0.4
5	5.69 ± 0.4	3.97 ± 0.4	5.68 ± 0.5	6.36 ± 0.6	4.81 ± 0.9	7.43 ± 1.2
6	6.65 ± 0.3	4.84 ± 0.5	7.10 ± 0.5	3.93 ± 0.5	4.44 ± 0.6	11.68 ± 1.4
7	n.s.	n.s.	n.s.	n.s.	n.s.	n.s.
8	1.78 ± 0.1	1.30 ± 0.2	1.41 ± 0.1	1.88 ± 0.2	1.75 ± 0.2	2.41 ± 0.5
9	1.71 ± 0.1	1.18 ± 0.1	1.52 ± 0.0	2.19 ± 0.1	1.58 ± 0.2	2.65 ± 0.5
10	2.18 ± 0.2	0.98 ± 0.1	1.90 ± 0.1	2.27 ± 0.4	2.04 ± 0.1	1.50 ± 0.2
11	1.00 ± 0.1	1.02 ± 0.03	1.17 ± 0.2	0.78 ± 0.4	0.88 ± 0.1	1.23 ± 0.3
12	2.03 ± 0.1	1.96 ± 0.3	1.63 ± 0.1	1.68 ± 0.1	1.588 ± 0.1	1.94 ± 0.2
13	3.08 ± 0.1	2.07 ± 0.1	2.82 ± 0.3	4.15 ± 0.2	3.79 ± 0.3	3.08 ± 0.3
14	1.57 ± 0.8	1.55 ± 0.1	1.64 ± 0.1	1.80 ± 0.1	1.79 ± 0.2	1.69 ± 0.1
15	2.36 ± 0.1	2.33 ± 0.2	2.40 ± 0.1	1.48 ± 0.2	2.02 ± 0.2	2.76 ± 0.3
16	1.91 ± 0.1	1.54 ± 0.1	1.54 ± 0.1	2.38 ± 0.1	1.52 ± 0.1	2.45 ± 0.1
17	6.35 ± 0.5	5.01 ± 0.8	5.55 ± 0.6	9.19 ± 0.5	6.36 ± 1.8	9.87 ± 1.3
18	3.38 ± 0.2	1.72 ± 0.2	2.65 ± 0.2	4.00 ± 0.2	3.34 ± 0.3	4.02 ± 0.7
19	2.97 ± 0.2	1.84 ± 0.2	2.05 ± 0.2	3.30 ± 0.2	2.95 ± 0.4	4.64 ± 0.6
20	3.13 ± 0.1	1.88 ± 0.3	2.84 ± 0.2	4.35 ± 0.2	2.66 ± 0.2	3.46 ± 0.3

* EC_50_ values in μM from SRB assays after 72 h of treatment, the values are averaged from three independent experiments performed each in triplicate, confidence interval CI = 95%; mean ± standard mean error, cut-off 30 µM. n.s.—not soluble.

**Table 2:** Selectivity * of compounds **1**, **2**, **4**–**6**, **8**–**20**.

Compound	SI *				
	A375	HT29	MCF-7	A2780	FaDu
1	0.63	1.00	0.84	0.67	0.69
2	1.03	1.27	1.34	1.04	1.29
4	1.15	1.62	1.20	1.06	1.64
5	1.31	1.87	1.29	1.17	1.54
6	1.76	2.60	1.65	2.97	2.63
8	1.35	1.85	1.71	1.28	1.38
9	1.55	2.25	1.74	1.21	1.68
10	0.68	1.53	0.79	0.66	0.74
11	1.23	0.62	1.05	1.57	1.40
12	0.96	0.99	1.19	1.15	1.23
13	1.00	1.51	1.09	0.74	0.81
14	1.08	1.09	1.03	0.94	0.94
15	1.17	1.18	1.15	1.86	1.37
16	1.28	1.59	1.59	1.03	1.61
17	1.55	1.97	1.78	1.07	1.55
18	1.19	2.34	1.52	1.01	1.20
19	1.56	2.52	2.26	1.41	1.57
20	1.11	1.84	1.22	0.79	1.30

* Selectivity index (SI) is defined as: SI = EC50 (NIH 3T3)/EC50 (tumor cell line).

**Table 3:** Anticancer screening data in concentration 10 µM.

Comp. (NSC)	60 Cell Lines Assay in 1 Dose 10 µM Concentration					
	Mean Growth, %	Range of Growth, %	Most Sensitive Cell Lines	Growth % of the Most Sensitive Cell Lines	Positive Cytostatic Effect ^a^	Positive Cytotoxic Effect ^b^
1 (797815)	20.76	−82.49 to 84.36	SK-MEL-5 (Melanoma)	−82.49	27/59	16/59
			HCT-116 (Colon cancer)	−78.96		
2 (799588)	99.25	68.07 to 136.13	HT29 (Colon cancer)	68.07	0/59	0/59
3 (761972)	18.91	−88.44 to 89.35	COLO 205 (Colon cancer)	−88.44	20/57	19/57
			LOX IMVI (Melanoma)	−88.23		
			HCT-116 (Colon cancer)	−85.85		
			M14 (Melanoma)	−82.21		
			SK-MEL-28 (Melanoma)	−81.53		
4 (797816)	−31.86	−99.04 to 23.04	NCI-H226 (NSC lung cancer)	−99.04	18/59	40/59
			LOX IMVI (Melanoma)	−94.90		
			HCT-116 (Colon cancer)	−94.73		
			SK-MEL-28 (Melanoma)	−84.86		
			NCI-H460 (NSC lung cancer)	−83.30		
			U251 (CNS cancer)	−83.30		
5 (797798)	56.19	−4.98 to 98.92	COLO 205 (Colon cancer)	−4.98	13/58	2/58
			SR (leukemia)	−1.30		
6 (804743)	−77.22	−99.73 to −5.08	NCI-H322M (NSC lung cancer)	−99.73	−	59/59
			OVCAR-5 (Ovarian cancer)	−98.74		
			UO-31 (Renal cancer)	−98.13		
			SNB-75 (CNS cancer)	−97.70		
			CAKI-1 (Renal cancer)	−96.49		
			MDA-MB-435 (Melanoma)	−95.56		
			ACHN (Renal cancer)	−95.35		
7 (797792)	−27.32	−99.38 to 76.37	HCT-116 (Colon cancer)	−99.38	16/59	41/58
			786-0 (Renal cancer)	−96.23		
			LOX IMVI (Melanoma)	−94.90		
			CAKI-1 (Renal cancer)	−92.03		
8 (801866)	−46.72	−99.54 to 75.89	LOX IMVI (Melanoma)	−99.54	7/60	49/60
			HCT-116 (Colon cancer)	−96.24		
			IGROV1 (Ovarian cancer)	−94.22		
			HCC-2998 (Colon cancer)	−93.75		
			OVCAR-3 (Ovarian cancer)	−93.30		
			CAKI-1 (Renal cancer)	−92.49		
			RXF 393 (Renal cancer)	−91.86		
9 (804757)	13.57	−100.00 to 106.93	LOX IMVI (Melanoma)	−100.00	15/58	24/58
			COLO 205 (Colon cancer)	−93.54		
			U251 (CNS cancer)	−90.81		
			OVCAR-8 (Ovarian cancer)	−87.34		
11 (799581)	−83.06	−97.92 to −46.66	A498 (Renal cancer)	−97.92	−	59/59
			OVCAR-3 (Ovarian cancer)	−95.88		
			ACHN (Renal cancer)	−95.87		
			SK-MEL-5 (Melanoma)	−95.48		
			SNB-75 (CNS cancer)	−95.12		
			HCT-116 (Colon cancer)	−94.79		
			TK-10 (Renal cancer)	−94.69		
12 (801870)	−3.21	−94.44 to 98.73	SN12C (Renal cancer)	−94.44	11/60	32/60
			OVCAR-5 (Ovarian cancer)	−87.72		
			IGROV1 (Ovarian cancer)	−87.10		
			LOX IMVI (Melanoma)	−86.54		
13 (806830)	74.89	−5.95 to 106.66	SR (leukemia)	−5.95	10/60	1/60
14 (799580)	−27.92	−100.00 to 110.43	HCT-116 (Colon cancer)	−100.00	3/59	42/59
			M14 (Melanoma)	−92.02		
			MDA-MB-435 (Melanoma)	−91.82		
15 (806835)	37.20	−87.74 to 103.57	LOX IMVI (Melanoma)	−87.74	17/60	14/60
			HCT-116 (Colon cancer)	−82.96		
16 (806829)	−0.81	−99.17 to 103.84	LOX IMVI (Melanoma)	−99.17	12/60	31/60
			U251 (CNS cancer)	−95.86		
			HCT-116 (Colon cancer)	−91.45		
			786-0 (Renal cancer)	−90.69		
17 (799502)	95.46	51.57 to 118.35	CAKI-1 (Renal cancer)	51.57	0/59	0/59
19 (811982)	−4.29	−98.96 to 84.53	LOX IMVI (Melanoma)	−98.96	20/60	28/60
			HCC-2998 (Colon cancer)	−93.35		
20 (811985)	−36.47	−100.00 to 90.80	LOX IMVI (Melanoma)	−100.00	8/60	46/60
			786-0 (Renal cancer)	−97.28		
			MDA-MB-435 (Melanoma)	−96.77		
			ACHN (Renal cancer)	−94.94		

^a^ Ratio between number of cell lines with percent growth from 0 to 50 and total number of cell lines.^b^ Ratio between number of cell lines with percent growth of < 0 and total number of cell lines.

**Table 4:** The selectivity indexes of compounds **1**, **3**, **4**, **6**, **7**–**9**, **11**, **12**, **14**–**16**, **19**, and **20** on the growth of tumor cell lines subpanel at the GI_50_, TGI, and LC_50_ levels.

**Panel**	**1**			**3**			**4**			**6**			**7**		
	**SI ^a^**	**SI ^b^**	**SI ^c^**	**SI ^a^**	**SI ^b^**	**SI ^c^**	**SI ^a^**	**SI ^b^**	**SI ^c^**	**SI ^a^**	**SI ^b^**	**SI ^c^**	**SI ^a^**	**SI ^b^**	**SI ^c^**
I *	1.00	0.78	0.17	1.88	0.28	0.39	1.25	1.01	0.95	1.39	1.29	0.76	0.86	0.66	0.18
II	0.99	0.99	2.69	1.33	0.87	0.89	0.52	0.97	1.19	1.33	1.18	1.07	1.13	1.11	2.55
III	1.12	1.11	2.92	1.92	**4.69**	**3.18**	1.08	1.08	1.24	0.82	0.79	0.87	1.06	1.17	2.81
IV	1.03	1.06	2.92	0.58	**3.75**	0.99	0.97	1.03	1.26	0.93	0.98	1.06	1.06	1.04	1.69
V	1.04	1.05	2.86	1.87	**3.59**	**3.23**	0.95	0.99	1.20	1.17	1.11	1.17	0.95	1.08	2.63
VI	1.03	1.05	2.80	0.49	0.81	0.87	0.86	0.92	0.67	0.90	0.86	1.00	0.90	0.91	1.40
VII	0.99	0.97	1.87	0.64	0.89	0.79	1.12	1.03	1.25	1.05	1.10	1.17	1.01	1.13	2.76
VIII	0.57	1.12	**3.09**	2.03	**3.85**	**4.63**	1.01	1.09	1.34	1.59	1.52	1.59	1.01	1.10	2.81
IX	0.99	0.94	1.52	1.23	1.06	0.91	0.95	0.89	0.61	0.63	0.69	0.76	1.00	1.02	2.83
**Panel**	**8**			**9**			**11**			**12**			**14**		
	**SI ^a^**	**SI ^b^**	**SI ^c^**	**SI ^a^**	**SI ^b^**	**SI ^c^**	**SI ^a^**	**SI ^b^**	**SI ^c^**	**SI ^a^**	**SI ^b^**	**SI ^c^**	**SI ^a^**	**SI ^b^**	**SI ^c^**
I	1.17	0.68	0.17	1.13	0.93	0.89	3.79	2.56	-	0.93	0.84	0.58	1.09	0.92	-
II	1.00	1.07	2.73	1.04	1.02	1.05	0.85	0.84	0.88	1.03	1.06	-	0.97	0.99	0.86
III	1.15	1.17	2.88	1.13	1.11	-	1.34	1.21	1.49	1.02	1.02	-	1.04	0.99	1.06
IV	0.97	1.07	2.83	0.96	0.97	0.97	0.85	0.86	0.98	0.99	0.98	-	1.03	1.06	-
V	0.94	1.02	2.51	0.99	1.01	1.04	0.91	0.92	0.92	0.95	1.00	-	0.97	1.01	1.04
VI	0.94	1.01	2.49	0.94	0.95	1.04	1.07	1.01	-	0.99	1.02	1.00	0.97	1.02	0.97
VII	0.97	1.06	2.73	1.02	1.03	1.06	0.88	0.90	0.97	1.01	1.05	0.99	1.04	1.03	1.16
VIII	1.01	1.10	2.83	0.98	1.02	1.06	0.98	0.96	-	1.04	1.09	-	0.98	1.02	-
IX	1.01	1.02	2.01	1.02	0.96	0.91	0.91	0.83	0.93	1.03	0.98	-	0.98	0.97	0.95
**Panel**	**15**				**16**				**19**				**20**		
	**SI ^a^**	**SI ^b^**	**SI ^c^**		**SI ^a^**	**SI ^b^**	**SI ^c^**		**SI ^a^**	**SI ^b^**	**SI ^c^**		**SI ^a^**	**SI ^b^**	**SI ^c^**
I	1.27	0.89	0.29		1.07	0.74	0.19		1.15	1.02	0.51		0.91	0.74	0.17
II	0.63	1.11	0.54		1.00	1.05	**3.12**		0.77	0.75	0.63		0.97	0.99	2.77
III	1.17	1.17	-		1.05	1.07	2.79		1.58	2.16	**4.67**		1.13	1.13	2.90
IV	1.12	0.92	1.09		0.97	1.03	2.99		1.23	1.19	1.35		0.99	1.06	2.86
V	1.09	1.07	1.77		0.97	1.05	**3.15**		0.92	1.18	2.21		0.97	1.04	2.84
VI	1.14	1.13	**4.81**		0.92	0.96	0.66		0.90	0.93	0.86		0.98	1.02	2.66
VII	0.93	0.69	2.02		1.03	1.10	**3.28**		0.91	0.84	1.28		1.03	1.09	2.95
VIII	1.16	1.16	**4.57**		1.03	1.10	**3.18**		0.99	0.74	0.91		0.97	1.05	1.43
IX	1.14	1.05	**3.17**		1.03	1.01	2.57		1.11	1.07	1.00		0.99	0.98	2.49

* I—Leukemia; II—NSCL cancer; III—Colon Cancer; IV—CNS cancer; V—Melanoma; VI—Ovarian Cancer; VII—Renal Cancer, VIII—Prostate cancer; IX—Breast cancer. ^a^ GI_50_; ^b^ TGI; ^c^ LC_50_. Bold values represent best results.

**Table 5:** The selectivity indexes of compounds **3**, **8**, **11**, **15**, and **16** on the growth of individual tumor cell lines at the GI_50_ and TGI levels (SI (GI_50_) ≥ 5.00).

Compound	Panel/Cell Line	SI (GI_50_)	SI (TGI)
3	Leukemia K-562	**9.56**	0.15
	Colon cancer HCT-116	2.33	**14.89**
	CNS cancer SF-539	0.35	**6.43**
	CNS cancer SNB-19	0.33	**5.93**
	CNS cancer SNB-75	0.39	**7.14**
8	Leukemia K-562	**5.41**	-
11	Leukemia CCRF-CEM	**5.16**	3.57
	Leukemia HL-60(TB)	**6.45**	**5.74**
	Leukemia RPMI-8226	**5.38**	4.19
	Colon cancer HT29	**5.38**	0.91
	Ovarian cancer OVCAR-4	**5.38**	2.81
15	Leukemia RPMI-8226	**5.85**	0.86
16	Leukemia RPMI-8226	**5.55**	0.76

Bold values represent best results.

## Supplementary Materials

The following are available online at https://www.mdpi.com/1422-0067/22/4/1714/s1, Figures S1–S32: Anticancer screening data of compounds **1–9**, **11–17**, **19** and **20**, Table S1–S3: Influence of compounds **1**, **3**, **4**, **6**, **7–9**, **11**, **12**, **14–16**, **19**, **20** on the growth of individual tumor cell panel.
