# Supplementary material for: Cytotoxic Potential of a-Azepano- and 3-Amino-3,4-SeCo-Triterpenoids
Source: Int J Mol Sci. 2021 Feb 8;22(4):1714. doi: 10.3390/ijms22041714 (PMC7914897; doi:10.3390/ijms22041714)
Supplement: Supplementary file 1 [file ijms-22-01714-s001.pdf]

Supporting Information

Cytotoxic potential of A-azepano- and 3-amino-3,4-seco-triterpenoids

Oxana Kazakova[1]*, Irina Smirnova[1], Elena Tret'yakova[1],
René Csuk[2], Sophie Hoenke[2], Lucie Fischer[2]

[1] Ufa Institute of Chemistry UFRC RAS, pr. Octyabrya 71, 450054 Ufa, Russian Federation
[2] Martin-Luther-University Halle-Wittenberg, Organic Chemistry, Kurt-Mothes-Str. 2, D-06120 Halle (Saale), Germany

Correspondence
Prof. Dr. Oxana B. Kazakova
Ufa Institute of Chemistry of the
Ufa Federal Research Centre of
the Russian Academy of Sciences
71 Prospect Oktyabrya
Ufa, 450054 Russian Federation
E-mail: obf@anrb.ru

Prof. Dr. René Csuk
Martin-Luther-University
Halle-Wittenberg,
Organic Chemistry,
Kurt-Mothes-Str. 2,
D-06120 Halle (Saale), Germany
E-mail: rene.csuk@chemie.uni-halle.de

Figure S1. Anticancer screening data of compound 1 at single dose assay

Developmental Therapeutics Program One Dose Mean Graph	NSC: D-797815 / 1	Conc: 1.00E-5 Molar	Test Date: May 01, 2017
	Experiment ID: 1705OS96		Report Date: May 18, 2017

Panel/Cell Line	Growth Percent
**Leukemia**	
CCRF-CEM	12.43
HL-60(TB)	-51.88
K-562	-26.59
MOLT-4	4.40
RPMI-8226	-36.88
SR	-40.44
**Non-Small Cell Lung Cancer**	
A549/ATCC	44.88
EKVX	63.26
HOP-62	78.96
HOP-92	81.99
NCI-H226	84.36
NCI-H23	62.57
NCI-H322M	69.84
NCI-H460	-54.44
NCI-H522	64.19
**Colon Cancer**	
COLO 205	-63.45
HCC-2998	-5.02
HCT-116	-78.86
HCT-15	3.28
HT29	2.58
KM12	23.20
SW-620	-66.45
**CNS Cancer**	
SF-268	61.21
SF-295	40.41
SF-539	34.26
SNB-19	49.62
SNB-75	77.06
U251	7.49
**Melanoma**	
LOX IMVI	-57.73
MALME-3M	-65.40
M14	-49.76
MDA-MB-435	13.55
SK-MEL-2	78.76
SK-MEL-28	-65.40
SK-MEL-5	-82.49
UACC-257	-8.72
UACC-62	43.16
**Ovarian Cancer**	
IGROV1	36.30
OVCAR-3	44.42
OVCAR-4	31.81
OVCAR-5	74.96
OVCAR-8	23.54
NCI/ADR-RES	44.82
SK-OV-3	92.20
**Renal Cancer**	
786-0	30.17
ACHN	50.20
CAKI-1	38.39
RXF 393	48.36
SN12C	45.93
TK-10	80.77
UO-31	53.76
**Prostate Cancer**	
PC-3	25.61
DU-145	49.53
**Breast Cancer**	
MCF7	-12.60
MDA-MB-231/ATCC	46.85
HS 578T	46.76
BT-549	77.10
T-47D	36.46
MDA-MB-468	11.74
Mean	20.76
Delta	103.25
Range	174.69

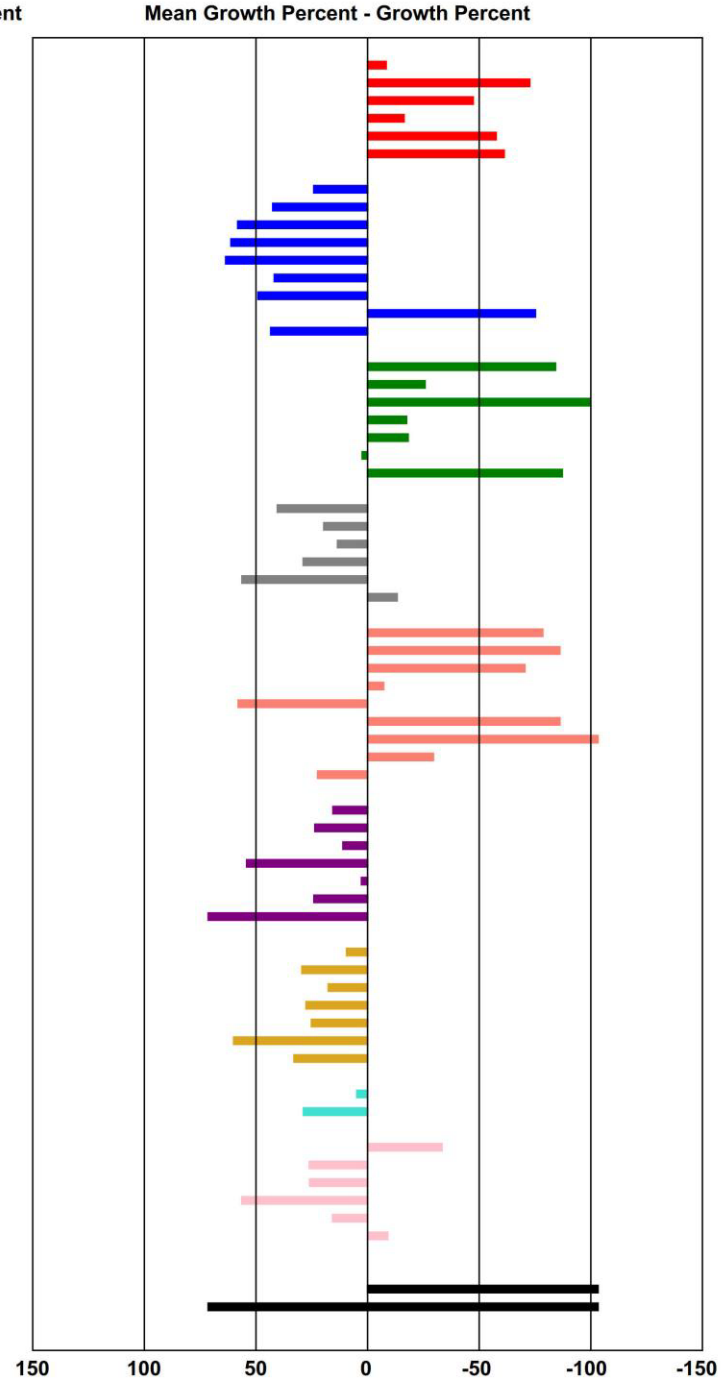

Figure S2. Anticancer screening data of compound 2 at single dose assay

Developmental Therapeutics Program One Dose Mean Graph	NSC: D-799588 / 1	Conc: 1.00E-5 Molar	Test Date: Jul 24, 2017
	Experiment ID: 1707OS42		Report Date: Aug 17, 2017

Panel/Cell Line	Growth Percent
Leukemia	
CCRF-CEM	103.44
HL-60(TB)	136.13
MOLT-4	95.32
RPMI-8226	98.64
SR	86.78
Non-Small Cell Lung Cancer	
A549/ATCC	111.67
EKVX	95.43
HOP-62	111.45
HOP-92	88.73
NCI-H226	106.68
NCI-H23	94.64
NCI-H322M	88.79
NCI-H460	100.32
NCI-H522	91.58
Colon Cancer	
COLO 205	105.64
HCC-2998	100.86
HCT-116	94.31
HCT-15	81.53
HT29	68.07
KM12	100.50
SW-620	102.99
CNS Cancer	
SF-268	91.20
SF-295	104.64
SF-539	105.10
SNB-19	99.05
SNB-75	86.05
U251	108.12
Melanoma	
LOX IMVI	95.60
MALME-3M	92.94
M14	93.63
MDA-MB-435	96.83
SK-MEL-2	105.21
SK-MEL-28	112.66
SK-MEL-5	92.71
UACC-257	123.03
UACC-62	94.66
Ovarian Cancer	
IGROV1	99.10
OVCAR-3	97.23
OVCAR-4	101.04
OVCAR-5	101.51
OVCAR-8	105.20
NCI/ADR-RES	96.36
SK-OV-3	121.52
Renal Cancer	
786-0	93.63
A498	95.26
ACHN	106.83
CAKI-1	97.14
RXF 393	103.51
SN12C	91.18
TK-10	110.20
UO-31	84.39
Prostate Cancer	
PC-3	86.01
DU-145	100.72
Breast Cancer	
MCF7	87.13
MDA-MB-231/ATCC	107.27
HS 578T	96.07
BT-549	100.88
T-47D	102.55
MDA-MB-468	106.17
Mean	99.25
Delta	31.18
Range	68.06

Mean Growth Percent - Growth Percent

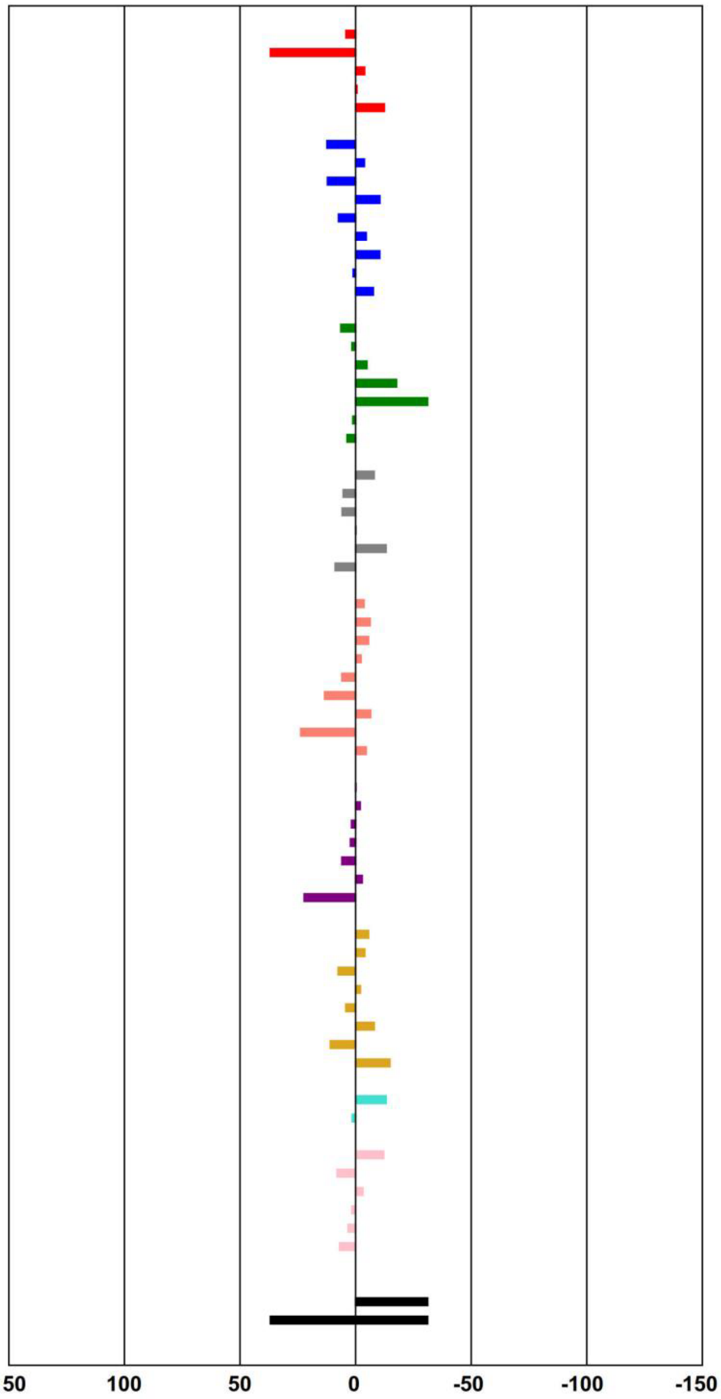

Figure S3. Anticancer screening data of compound 3 at single dose assay

Developmental Therapeutics Program One Dose Mean Graph	NSC: D-761972 / 1	Conc: 1.00E-5 Molar	Test Date: Oct 24, 2011
	Experiment ID: 1110OS52		Report Date: Nov 01, 2012

Panel/Cell Line	Growth Percent
**Leukemia**	
HL-60(TB)	40.15
K-562	-13.40
MOLT-4	-6.15
RPMI-8226	10.81
SR	-30.06
**Non-Small Cell Lung Cancer**	
A549/ATCC	47.00
EKVX	84.09
HOP-62	35.02
HOP-92	25.52
NCI-H23	45.76
NCI-H460	-16.51
NCI-H522	56.08
**Colon Cancer**	
COLO 205	-88.44
HCC-2998	-79.93
HCT-116	-85.85
HCT-15	6.06
HT29	9.52
KM12	25.62
SW-620	-17.40
**CNS Cancer**	
SF-268	71.10
SF-295	-50.49
SF-539	67.10
SNB-19	63.58
SNB-75	44.04
U251	11.93
**Melanoma**	
LOX IMVI	-88.23
MALME-3M	5.35
M14	-82.21
MDA-MB-435	-6.69
SK-MEL-2	68.76
SK-MEL-28	-81.53
SK-MEL-5	-8.81
UACC-257	-12.66
UACC-62	-49.34
**Ovarian Cancer**	
IGROV1	27.05
OVCAR-3	73.68
OVCAR-4	33.15
OVCAR-5	69.43
OVCAR-8	74.25
NCI/ADR-RES	44.88
SK-OV-3	71.79
**Renal Cancer**	
786-0	-1.18
A498	83.40
ACHN	26.00
CAKI-1	89.35
RXF 393	57.47
SN12C	58.75
TK-10	72.06
UO-31	64.03
**Prostate Cancer**	
PC-3	23.85
DU-145	59.54
**Breast Cancer**	
MCF7	-32.11
MDA-MB-231/ATCC	31.49
HS 578T	86.79
BT-549	35.02
T-47D	38.80
MDA-MB-468	-9.62
Mean	18.91
Delta	107.35
Range	177.79

Mean Growth Percent - Growth Percent

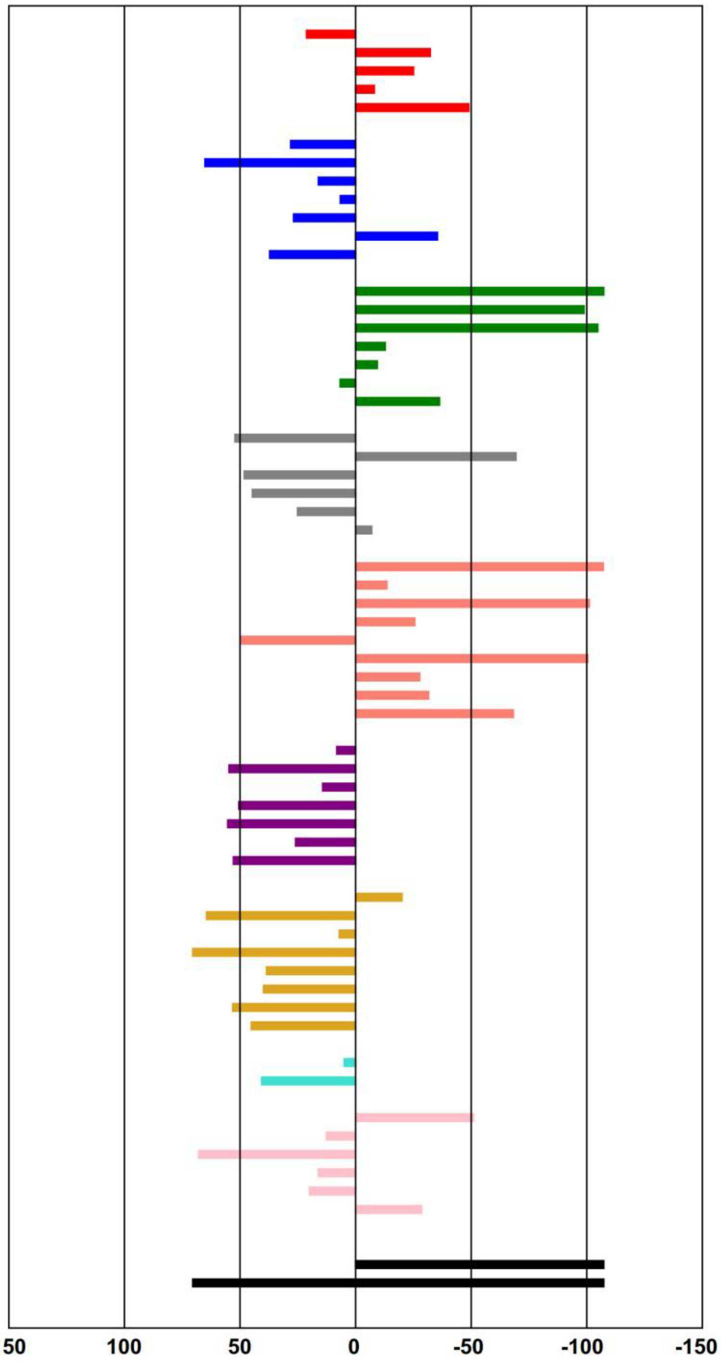

Figure S4. Anticancer screening data of compound 4 at single dose assay

Developmental Therapeutics Program<br>One Dose Mean Graph	NSC: D-797816 / 1	Conc: 1.00E-5 Molar	Test Date: May 08, 2017
	Experiment ID: 1705OS00		Report Date: May 26, 2017

Panel/Cell Line	Growth Percent
Leukemia	
CCRF-CEM	-34.70
HL-60(TB)	-54.98
K-562	-49.14
MOLT-4	-45.58
RPMI-8226	-39.72
SR	-49.90
Non-Small Cell Lung Cancer	
A549/ATCC	11.99
EKVX	19.40
HOP-62	-23.14
HOP-92	-5.92
NCI-H226	-99.04
NCI-H23	36.10
NCI-H322M	-17.88
NCI-H460	-83.30
NCI-H522	-77.84
Colon Cancer	
COLO 205	-78.29
HCC-2998	-38.73
HCT-116	-94.73
HCT-15	6.84
HT29	-75.38
KM12	0.12
SW-620	-69.61
CNS Cancer	
SF-268	5.71
SF-295	-64.59
SF-539	-36.47
SNB-19	-64.86
SNB-75	9.21
U251	-83.30
Melanoma	
LOX IMVI	-94.90
MALME-3M	-71.88
M14	-73.22
MDA-MB-435	-73.87
SK-MEL-2	13.06
SK-MEL-28	-84.86
SK-MEL-5	22.48
UACC-257	-36.88
UACC-62	-72.29
Ovarian Cancer	
IGROV1	-57.90
OVCAR-3	6.84
OVCAR-4	18.47
OVCAR-5	28.40
OVCAR-8	9.93
NCI/ADR-RES	17.62
SK-OV-3	69.60
Renal Cancer	
786-0	-80.95
ACHN	-66.33
CAKI-1	16.37
RXF 393	-27.89
SN12C	-1.54
TK-10	23.04
UO-31	12.51
Prostate Cancer	
PC-3	-43.18
DU-145	-17.44
Breast Cancer	
MCF7	-29.38
MDA-MB-231/ATCC	-54.76
HS 578T	10.53
BT-549	-65.99
T-47D	1.10
MDA-MB-468	-78.65
Mean	-31.86
Delta	67.18
Range	168.64

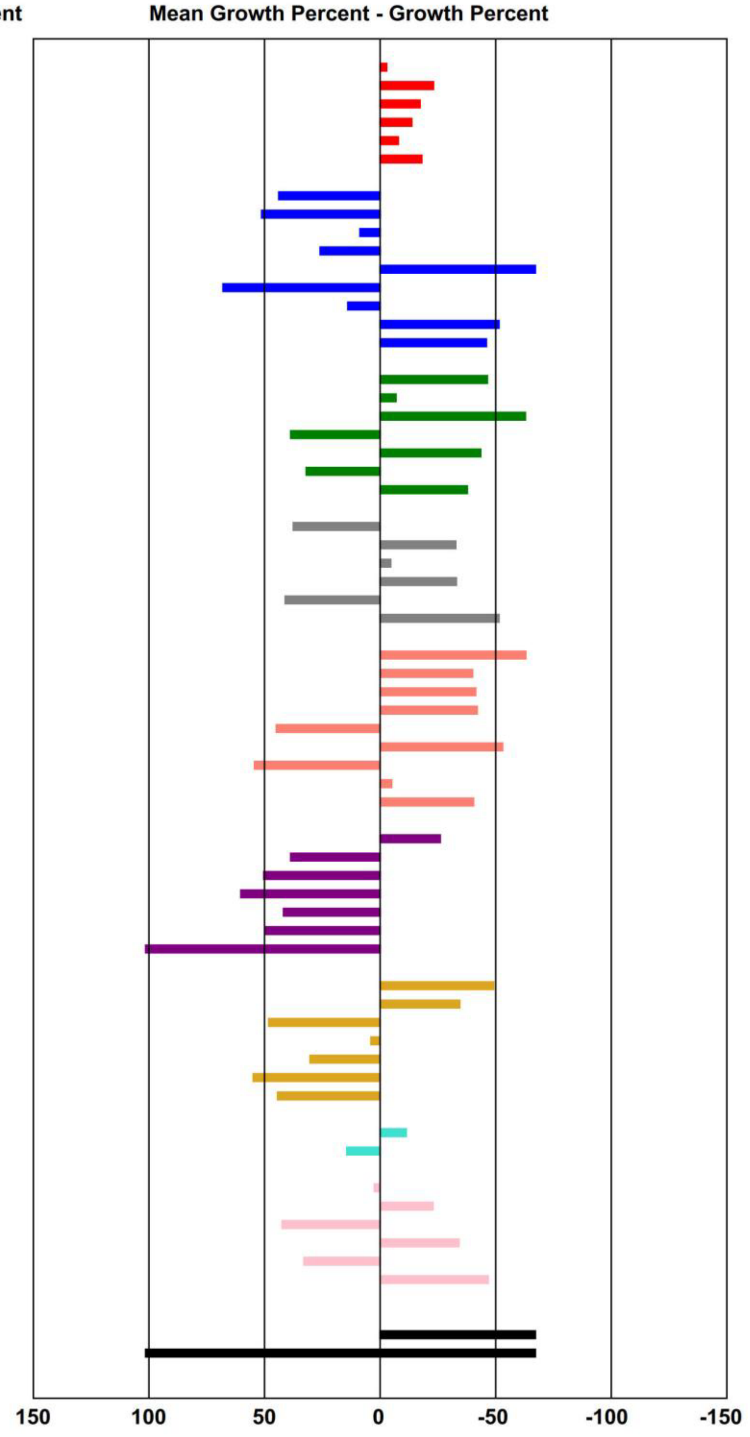

Figure S5. Anticancer screening data of compound 5 at single dose assay

Developmental Therapeutics Program<br>One Dose Mean Graph	NSC: D-797798 / 1	Conc: 1.00E-5 Molar	Test Date: May 01, 2017
	Experiment ID: 1705OS96		Report Date: May 18, 2017

Panel/Cell Line	Growth Percent
**Leukemia**	
HL-60(TB)	66.34
K-562	9.28
MOLT-4	33.75
RPMI-8226	20.14
SR	-1.30
**Non-Small Cell Lung Cancer**	
A549/ATCC	51.00
EKVX	86.50
HOP-62	88.83
HOP-92	38.29
NCI-H226	83.88
NCI-H23	81.34
NCI-H322M	81.75
NCI-H460	4.29
NCI-H522	73.77
**Colon Cancer**	
COLO 205	-4.98
HCC-2998	60.53
HCT-116	21.03
HCT-15	29.93
HT29	5.18
KM12	52.83
SW-620	74.07
**CNS Cancer**	
SF-268	67.13
SF-295	64.13
SF-539	64.62
SNB-19	61.63
SNB-75	98.92
U251	34.20
**Melanoma**	
LOX IMVI	45.18
MALME-3M	57.01
M14	33.62
MDA-MB-435	51.52
SK-MEL-2	48.41
SK-MEL-28	68.15
SK-MEL-5	50.66
UACC-257	55.10
UACC-62	73.57
**Ovarian Cancer**	
IGROV1	56.65
OVCAR-3	72.44
OVCAR-4	60.77
OVCAR-5	83.30
OVCAR-8	83.40
NCI/ADR-RES	74.20
SK-OV-3	93.11
**Renal Cancer**	
786-0	55.48
ACHN	54.29
CAKI-1	76.70
RXF 393	57.83
SN12C	62.15
TK-10	63.30
UO-31	69.31
**Prostate Cancer**	
PC-3	35.14
DU-145	75.58
**Breast Cancer**	
MCF7	40.73
MDA-MB-231/ATCC	57.99
HS 578T	52.67
BT-549	78.50
T-47D	57.67
MDA-MB-468	67.61
Mean	56.19
Delta	61.17
Range	103.90

Mean Growth Percent - Growth Percent

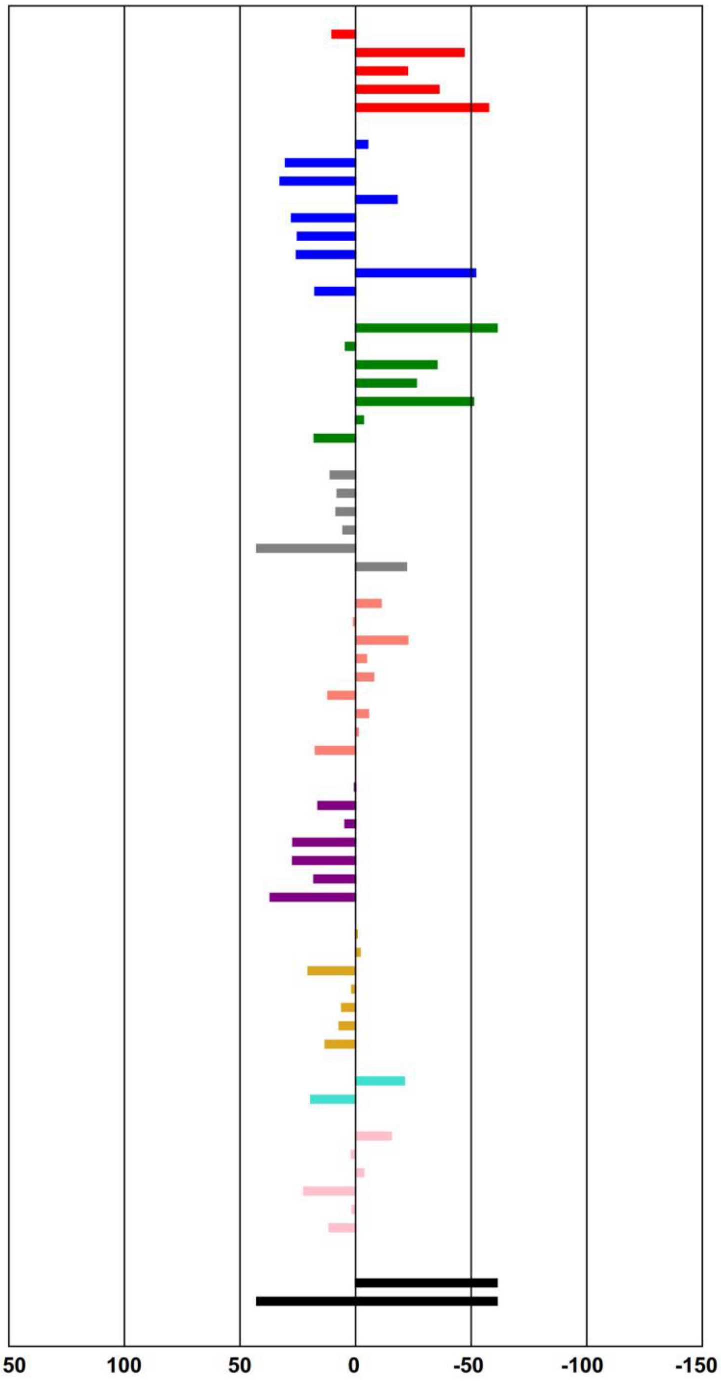

Figure S6. Anticancer screening data of compound 6 at single dose assay

Developmental Therapeutics Program One Dose Mean Graph	NSC: D-804743 / 1	Conc: 1.00E-5 Molar	Test Date: Apr 30, 2018
	Experiment ID: 1804OS61		Report Date: May 16, 2018

Panel/Cell Line	Growth Percent
**Leukemia**	
CCRF-CEM	-44.06
HL-60(TB)	-52.80
K-562	-25.45
MOLT-4	-56.65
RPMI-8226	-43.82
SR	-5.08
**Non-Small Cell Lung Cancer**	
A549/ATCC	-66.53
EKVX	-87.12
HOP-62	-74.48
HOP-92	-76.37
NCI-H226	-59.08
NCI-H23	-84.89
NCI-H322M	-99.73
NCI-H460	-81.57
NCI-H522	-75.62
**Colon Cancer**	
COLO 205	-55.90
HCC-2998	-78.81
HCT-116	-77.38
HCT-15	-89.29
HT29	-62.15
KM12	-85.53
SW-620	-80.66
**CNS Cancer**	
SF-268	-69.91
SF-295	-90.09
SF-539	-93.29
SNB-19	-90.39
SNB-75	-97.70
U251	-59.66
**Melanoma**	
LOX IMVI	-91.45
MALME-3M	-82.63
M14	-82.08
MDA-MB-435	-95.56
SK-MEL-2	-84.91
SK-MEL-28	-97.33
SK-MEL-5	-91.52
UACC-257	-80.55
UACC-62	-94.26
**Ovarian Cancer**	
IGROV1	-84.51
OVCAR-3	-78.83
OVCAR-5	-98.74
OVCAR-8	-57.82
NCI/ADR-RES	-86.43
SK-OV-3	-80.14
**Renal Cancer**	
786-0	-71.98
A498	-88.73
ACHN	-95.35
CAKI-1	-96.49
RXF 393	-70.42
SN12C	-93.98
TK-10	-78.35
UO-31	-98.13
**Prostate Cancer**	
PC-3	-79.09
DU-145	-89.53
**Breast Cancer**	
MCF7	-70.69
MDA-MB-231/ATCC	-94.00
HS 578T	-46.83
BT-549	-87.92
T-47D	-63.85
MDA-MB-468	-79.54
Mean	-77.22
Delta	22.51
Range	94.65

Mean Growth Percent - Growth Percent

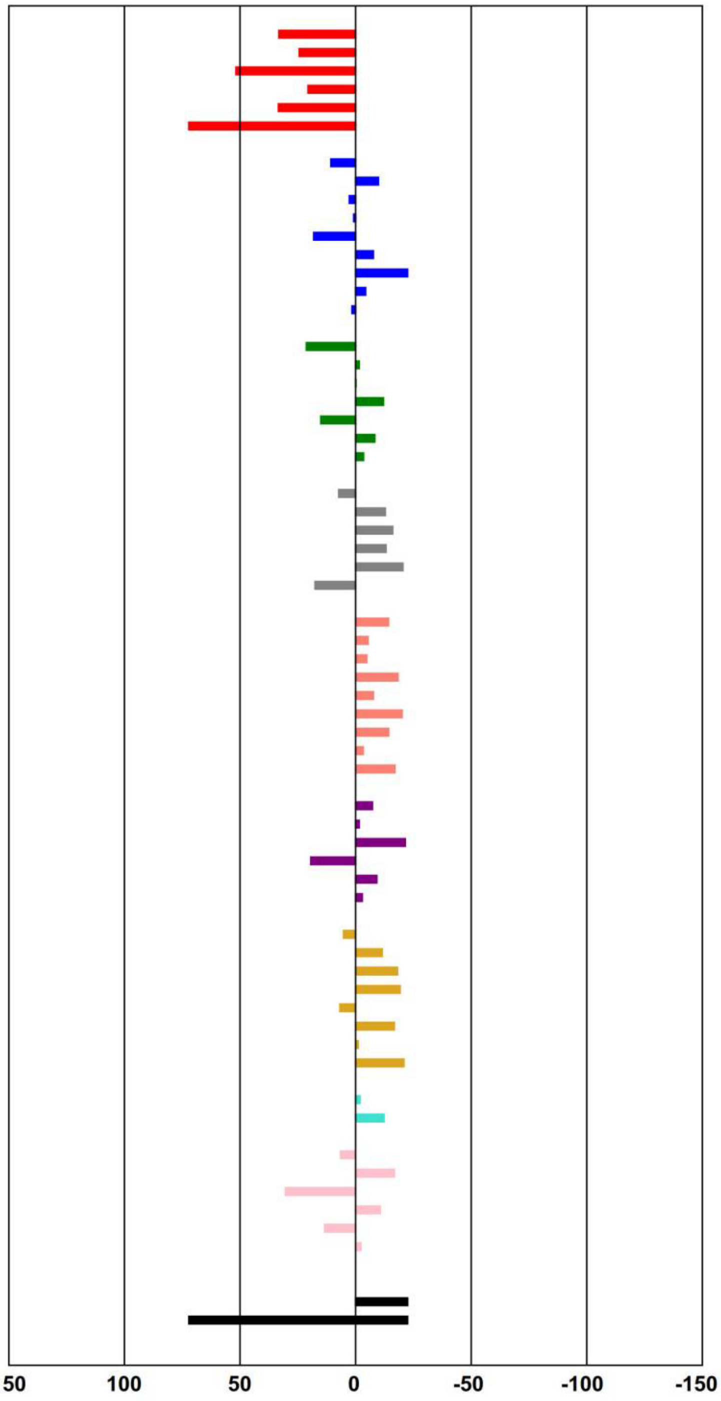

Figure S7. Anticancer screening data of compound 7 at single dose assay

Developmental Therapeutics Program

One Dose Mean Graph

**NSC:** D-797792 / 1	**Conc:** 1.00E-5 Molar	**Test Date:** May 01, 2017
**Experiment ID:** 1705OS96		**Report Date:** May 18, 2017

Panel/Cell Line	Growth Percent
Leukemia	
CCRF-CEM	-6.07
HL-60(TB)	-65.48
K-562	-39.70
MOLT-4	-32.74
RPMI-8226	-29.65
SR	-30.19
Non-Small Cell Lung Cancer	
A549/ATCC	-45.63
EKVX	23.19
HOP-62	27.66
HOP-92	-37.20
NCI-H226	34.81
NCI-H23	41.22
NCI-H322M	-21.40
NCI-H460	-60.64
NCI-H522	9.35
Colon Cancer	
COLO 205	-68.17
HCC-2998	-55.47
HCT-116	-99.38
HCT-15	-63.69
HT29	-49.41
KM12	-69.00
SW-620	-71.93
CNS Cancer	
SF-268	38.23
SF-295	-65.01
SF-539	38.73
SNB-19	-20.21
SNB-75	3.81
U251	-75.61
Melanoma	
LOX IMVI	-94.90
MALME-3M	-63.41
M14	-77.21
MDA-MB-435	-80.01
SK-MEL-2	10.51
SK-MEL-28	27.70
SK-MEL-5	-7.69
UACC-257	45.08
UACC-62	-51.12
Ovarian Cancer	
IGROV1	-58.73
OVCAR-3	-31.21
OVCAR-4	-51.22
OVCAR-5	8.55
OVCAR-8	-45.63
NCI/ADR-RES	-19.60
SK-OV-3	75.76
Renal Cancer	
786-0	-96.23
ACHN	-88.31
CAKI-1	-92.03
RXF 393	-65.27
SN12C	17.72
TK-10	0.42
UO-31	2.86
Prostate Cancer	
PC-3	-10.65
DU-145	-8.90
Breast Cancer	
MCF7	-25.03
MDA-MB-231/ATCC	-62.85
HS 578T	-9.12
BT-549	76.37
T-47D	2.46
MDA-MB-468	-50.53
Mean	-27.32
Delta	72.06
Range	175.75

Mean Growth Percent - Growth Percent

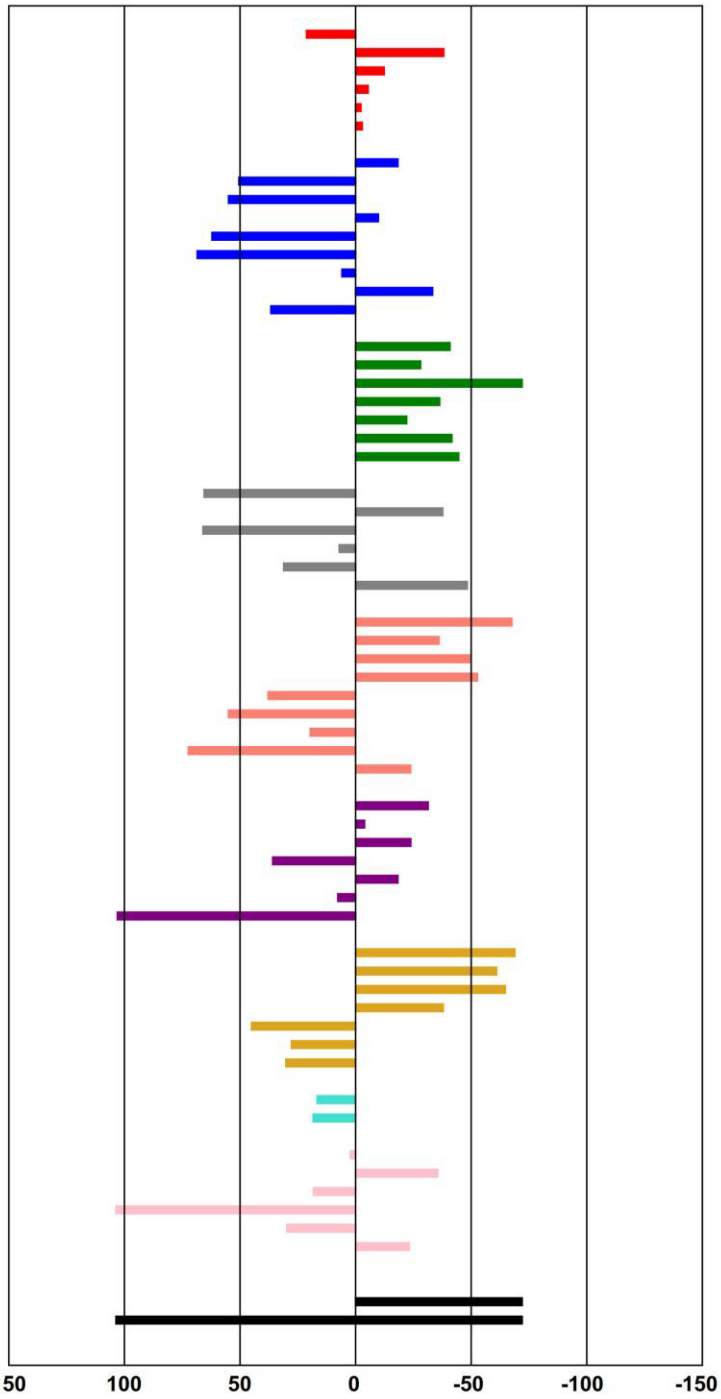

Figure S8. Anticancer screening data of compound 8 at single dose assay

Developmental Therapeutics Program One Dose Mean Graph	NSC: D-801866 / 1	Conc: 1.00E-5 Molar	Test Date: Nov 13, 2017
	Experiment ID: 1711OS00		Report Date: Dec 09, 2017

Panel/Cell Line	Growth Percent
Leukemia	
CCRF-CEM	-23.41
HL-60(TB)	-12.30
K-562	-44.63
MOLT-4	-46.37
RPMI-8226	-49.24
SR	-45.18
Non-Small Cell Lung Cancer	
A549/ATCC	-78.09
EKVX	-47.77
HOP-62	-63.53
HOP-92	-32.54
NCI-H226	23.24
NCI-H23	-58.81
NCI-H322M	-42.30
NCI-H460	-34.61
NCI-H522	-74.15
Colon Cancer	
COLO 205	-81.03
HCC-2998	-93.75
HCT-116	-96.24
HCT-15	-88.99
HT29	-83.75
KM12	-79.04
SW-620	-58.94
CNS Cancer	
SF-268	12.33
SF-295	-84.70
SF-539	9.45
SNB-19	-77.17
SNB-75	23.47
U251	-98.77
Melanoma	
LOX IMVI	-99.54
MALME-3M	-56.11
M14	-82.08
MDA-MB-435	-71.05
SK-MEL-2	-52.34
SK-MEL-28	-42.34
SK-MEL-5	64.23
UACC-257	-67.40
UACC-62	-79.00
Ovarian Cancer	
IGROV1	-94.22
OVCAR-3	-93.30
OVCAR-4	-67.50
OVCAR-5	16.76
OVCAR-8	-84.38
NCI/ADR-RES	-52.24
SK-OV-3	92.97
Renal Cancer	
786-0	-89.53
A498	69.82
ACHN	-82.43
CAKI-1	-92.49
RXF 393	-91.86
SN12C	-87.88
TK-10	-46.59
UO-31	18.40
Prostate Cancer	
PC-3	-21.40
DU-145	-36.79
Breast Cancer	
MCF7	-72.85
MDA-MB-231/ATCC	-76.55
HS 578T	-30.86
BT-549	75.89
T-47D	33.94
MDA-MB-468	-77.92
Mean	-46.72
Delta	52.82
Range	192.51

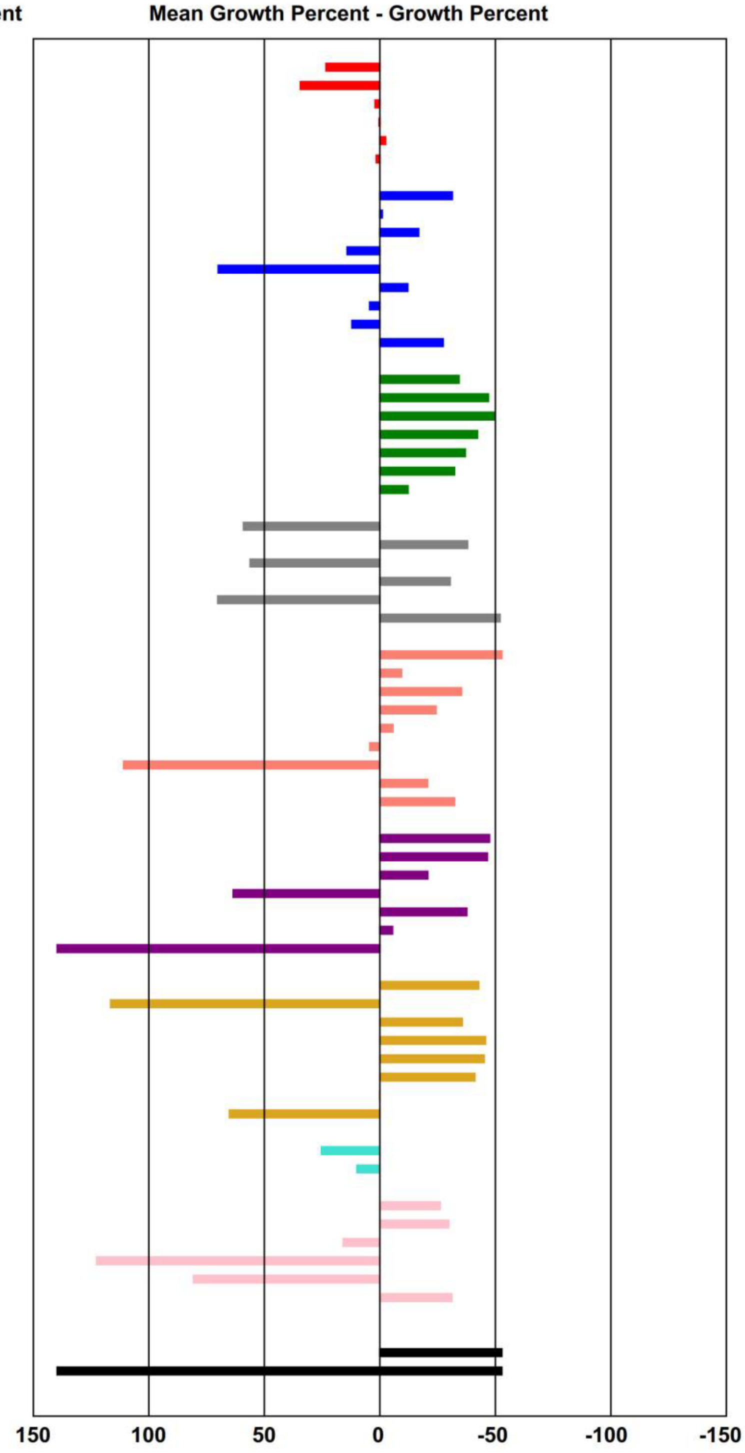

Figure S9. Anticancer screening data of compound 9 at single dose assay

Developmental Therapeutics Program One Dose Mean Graph	NSC: D-804757 / 1	Conc: 1.00E-5 Molar	Test Date: May 21, 2018
	Experiment ID: 1805OS64		Report Date: Jun 08, 2018

Panel/Cell Line	Growth Percent
Leukemia	
CCRF-CEM	5.21
HL-60(TB)	21.72
MOLT-4	-8.43
RPMI-8226	-32.81
SR	5.05
Non-Small Cell Lung Cancer	
A549/ATCC	-13.99
EKVX	84.53
HOP-62	104.59
HOP-92	23.79
NCI-H226	106.93
NCI-H23	63.08
NCI-H322M	20.66
NCI-H460	-70.37
NCI-H522	96.08
Colon Cancer	
COLO 205	-93.54
HCC-2998	-51.50
HCT-116	-32.08
HCT-15	-57.84
HT29	-55.24
KM12	-53.04
SW-620	-66.28
CNS Cancer	
SF-268	40.23
SF-295	-35.20
SF-539	77.48
SNB-19	18.12
SNB-75	76.51
U251	-90.81
Melanoma	
LOX IMVI	-100.00
MALME-3M	3.21
M14	15.48
MDA-MB-435	-17.26
SK-MEL-2	94.56
SK-MEL-28	63.75
SK-MEL-5	89.93
UACC-257	76.67
UACC-62	92.40
Ovarian Cancer	
IGROV1	40.52
OVCAR-3	-28.90
OVCAR-4	-40.99
OVCAR-5	41.09
OVCAR-8	-87.34
NCI/ADR-RES	-45.12
SK-OV-3	102.78
Renal Cancer	
786-0	-72.53
A498	60.16
ACHN	8.91
CAKI-1	30.45
SN12C	48.28
TK-10	80.51
UO-31	55.67
Prostate Cancer	
PC-3	27.74
DU-145	-7.26
Breast Cancer	
MCF7	-28.23
MDA-MB-231/ATCC	-7.94
HS 578T	92.86
BT-549	97.06
T-47D	65.05
MDA-MB-468	-47.15
Mean	13.57
Delta	113.57
Range	206.93

Mean Growth Percent - Growth Percent

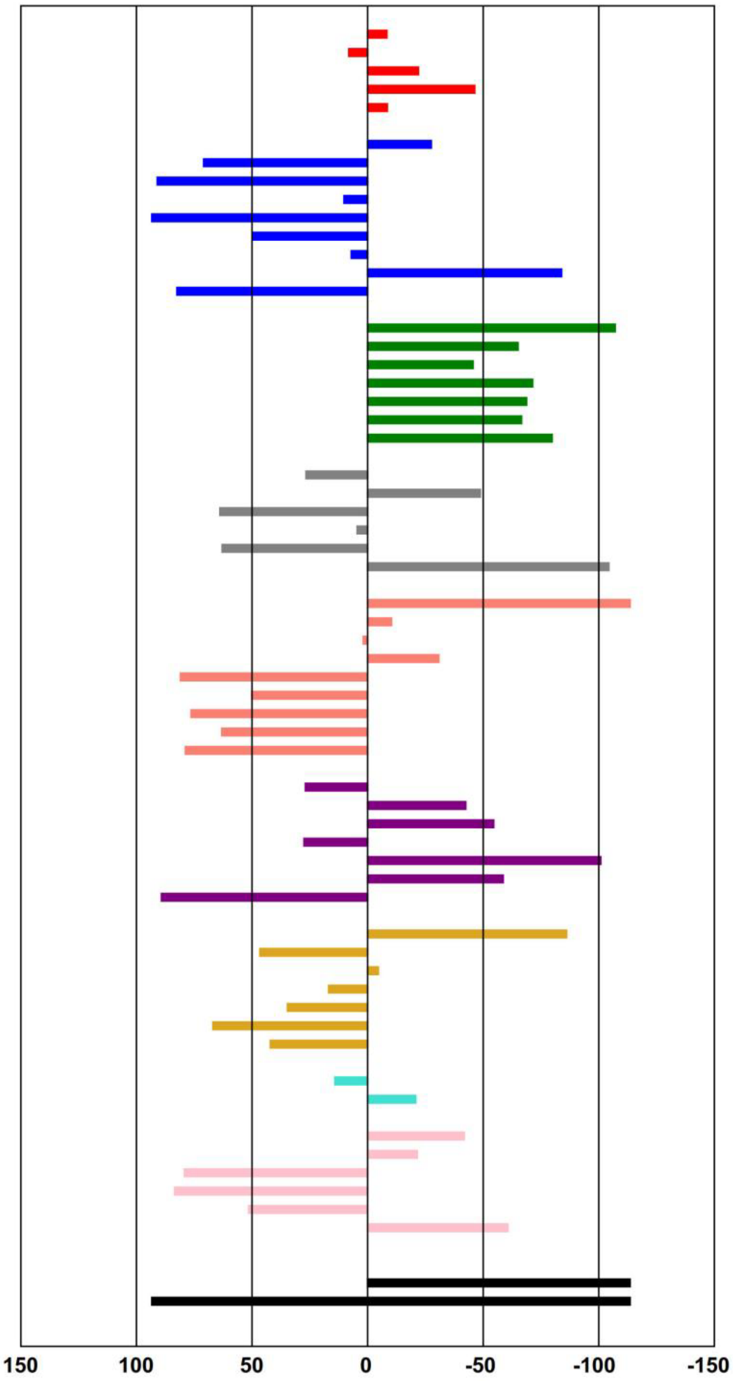

Figure S10. Anticancer screening data of compound 11 at single dose assay

Developmental Therapeutics Program One Dose Mean Graph	NSC: D-799581 / 1	Conc: 1.00E-5 Molar	Test Date: Jul 24, 2017
	Experiment ID: 1707OS42		Report Date: Aug 17, 2017

Panel/Cell Line	Growth Percent
**Leukemia**	
CCRF-CEM	-46.66
HL-60(TB)	-73.26
MOLT-4	-62.57
RPMI-8226	-49.28
SR	-56.78
**Non-Small Cell Lung Cancer**	
A549/ATCC	-83.13
EKVX	-84.14
HOP-62	-86.15
HOP-92	-88.51
NCI-H226	-55.75
NCI-H23	-71.93
NCI-H322M	-86.00
NCI-H460	-86.20
NCI-H522	-79.22
**Colon Cancer**	
COLO 205	-83.45
HCC-2998	-84.68
HCT-116	-94.79
HCT-15	-82.28
HT29	-86.12
KM12	-80.70
SW-620	-82.33
**CNS Cancer**	
SF-268	-81.22
SF-295	-91.75
SF-539	-87.70
SNB-19	-88.04
SNB-75	-95.12
U251	-89.74
**Melanoma**	
LOX IMVI	-86.23
MALME-3M	-82.91
M14	-90.18
MDA-MB-435	-93.55
SK-MEL-2	-92.47
SK-MEL-28	-92.33
SK-MEL-5	-95.48
UACC-257	-88.77
UACC-62	-85.61
**Ovarian Cancer**	
IGROV1	-78.77
OVCAR-3	-95.88
OVCAR-4	-80.86
OVCAR-5	-93.52
OVCAR-8	-76.35
NCI/ADR-RES	-67.61
SK-OV-3	-96.25
**Renal Cancer**	
786-0	-90.70
A498	-97.92
ACHN	-95.87
CAKI-1	-93.50
RXF 393	-91.45
SN12C	-83.28
TK-10	-94.69
UO-31	-92.62
**Prostate Cancer**	
PC-3	-84.86
DU-145	-87.37
**Breast Cancer**	
MCF7	-69.74
MDA-MB-231/ATCC	-87.12
HS 578T	-60.53
BT-549	-88.14
T-47D	-58.03
MDA-MB-468	-90.49
Mean	-83.06
Delta	14.86
Range	51.26

Mean Growth Percent - Growth Percent

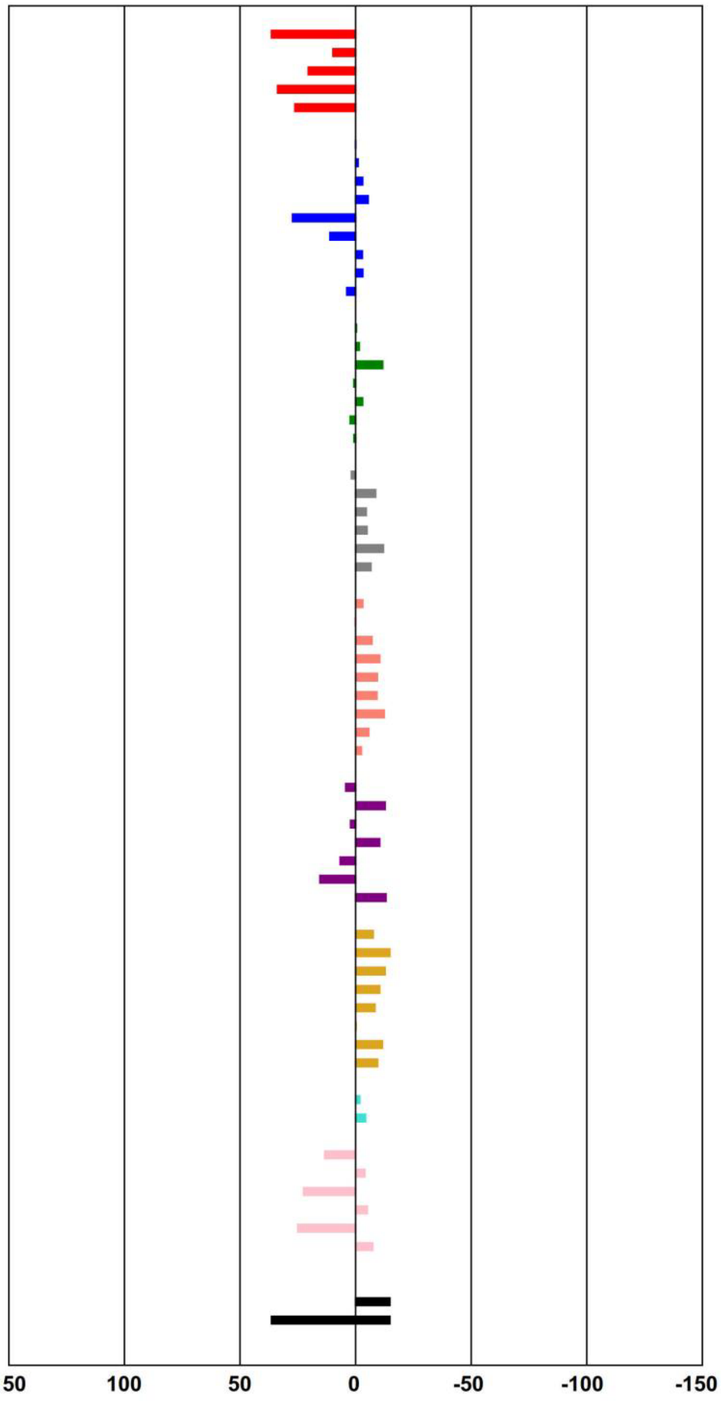

Figure S11. Anticancer screening data of compound 12 at single dose assay

Developmental Therapeutics Program One Dose Mean Graph	NSC: D-801870 / 1	Conc: 1.00E-5 Molar	Test Date: Nov 13, 2017
	Experiment ID: 1711OS00		Report Date: Dec 09, 2017

Panel/Cell Line	Growth Percent
**Leukemia**	
CCRF-CEM	1.15
HL-60(TB)	-34.96
K-562	-33.39
MOLT-4	-11.10
RPMI-8226	-14.81
SR	-46.14
**Non-Small Cell Lung Cancer**	
A549/ATCC	-12.00
EKVX	9.77
HOP-62	69.02
HOP-92	19.03
NCI-H226	82.14
NCI-H23	25.70
NCI-H322M	39.70
NCI-H460	-49.08
NCI-H522	-40.98
**Colon Cancer**	
COLO 205	-86.09
HCC-2998	-75.78
HCT-116	-82.77
HCT-15	-61.50
HT29	-50.82
KM12	-25.19
SW-620	-69.40
**CNS Cancer**	
SF-268	48.23
SF-295	-11.53
SF-539	71.90
SNB-19	38.96
SNB-75	81.57
U251	-82.13
**Melanoma**	
LOX IMVI	-86.54
MALME-3M	4.10
M14	-20.64
MDA-MB-435	-25.35
SK-MEL-2	83.12
SK-MEL-28	68.35
SK-MEL-5	92.12
UACC-257	58.52
UACC-62	87.68
**Ovarian Cancer**	
IGROV1	-87.10
OVCAR-3	-87.72
OVCAR-4	-64.39
OVCAR-5	63.30
OVCAR-8	-77.60
NCI/ADR-RES	-3.42
SK-OV-3	98.73
**Renal Cancer**	
786-0	-77.52
A498	81.33
ACHN	44.63
CAKI-1	-68.31
RXF 393	-79.87
SN12C	-94.44
TK-10	43.18
UO-31	60.97
**Prostate Cancer**	
PC-3	10.45
DU-145	0.90
**Breast Cancer**	
MCF7	-32.58
MDA-MB-231/ATCC	-10.26
HS 578T	61.41
BT-549	83.72
T-47D	59.60
MDA-MB-468	-78.23
Mean	-3.21
Delta	91.23
Range	193.17

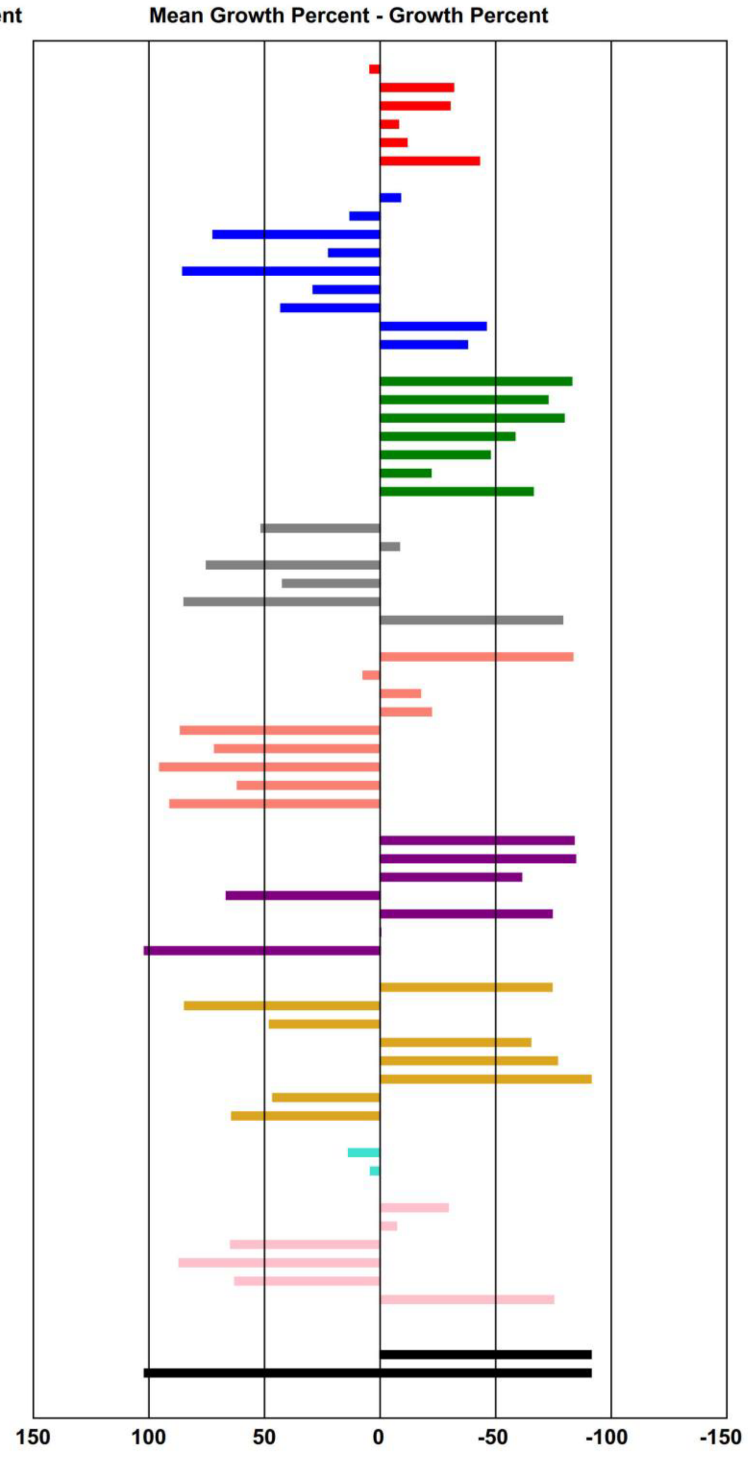

Figure S12. Anticancer screening data of compound 13 at single dose assay

Developmental Therapeutics Program One Dose Mean Graph	NSC: D-806830 / 1	Conc: 1.00E-5 Molar	Test Date: Aug 27, 2018
	Experiment ID: 1808OS00		Report Date: Sep 19, 2018

Panel/Cell Line	Growth Percent
Leukemia	
CCRF-CEM	41.83
HL-60(TB)	31.23
K-562	1.94
MOLT-4	35.13
RPMI-8226	36.05
SR	-5.95
Non-Small Cell Lung Cancer	
A549/ATCC	89.79
EKVX	90.87
HOP-62	89.02
HOP-92	26.43
NCI-H226	91.05
NCI-H23	103.64
NCI-H322M	95.30
NCI-H460	78.31
NCI-H522	96.84
Colon Cancer	
COLO 205	77.78
HCC-2998	55.97
HCT-116	44.43
HCT-15	60.52
HT29	10.58
KM12	95.99
SW-620	79.82
CNS Cancer	
SF-268	85.75
SF-295	102.17
SF-539	90.37
SNB-19	84.78
SNB-75	87.15
U251	68.86
Melanoma	
LOX IMVI	69.61
MALME-3M	106.33
M14	71.38
MDA-MB-435	73.37
SK-MEL-2	106.66
SK-MEL-28	105.97
SK-MEL-5	90.72
UACC-257	105.72
UACC-62	78.46
Ovarian Cancer	
IGROV1	65.07
OVCAR-3	97.34
OVCAR-4	87.74
OVCAR-5	101.36
OVCAR-8	97.00
NCI/ADR-RES	85.60
SK-OV-3	100.41
Renal Cancer	
786-0	65.47
A498	85.07
ACHN	101.28
CAKI-1	77.34
RXF 393	78.43
SN12C	78.61
TK-10	95.51
UO-31	66.48
Prostate Cancer	
PC-3	25.74
DU-145	93.70
Breast Cancer	
MCF7	83.88
MDA-MB-231/ATCC	70.12
HS 578T	85.61
BT-549	89.92
T-47D	69.76
MDA-MB-468	37.81
Mean	74.89
Delta	80.84
Range	112.61

Mean Growth Percent - Growth Percent

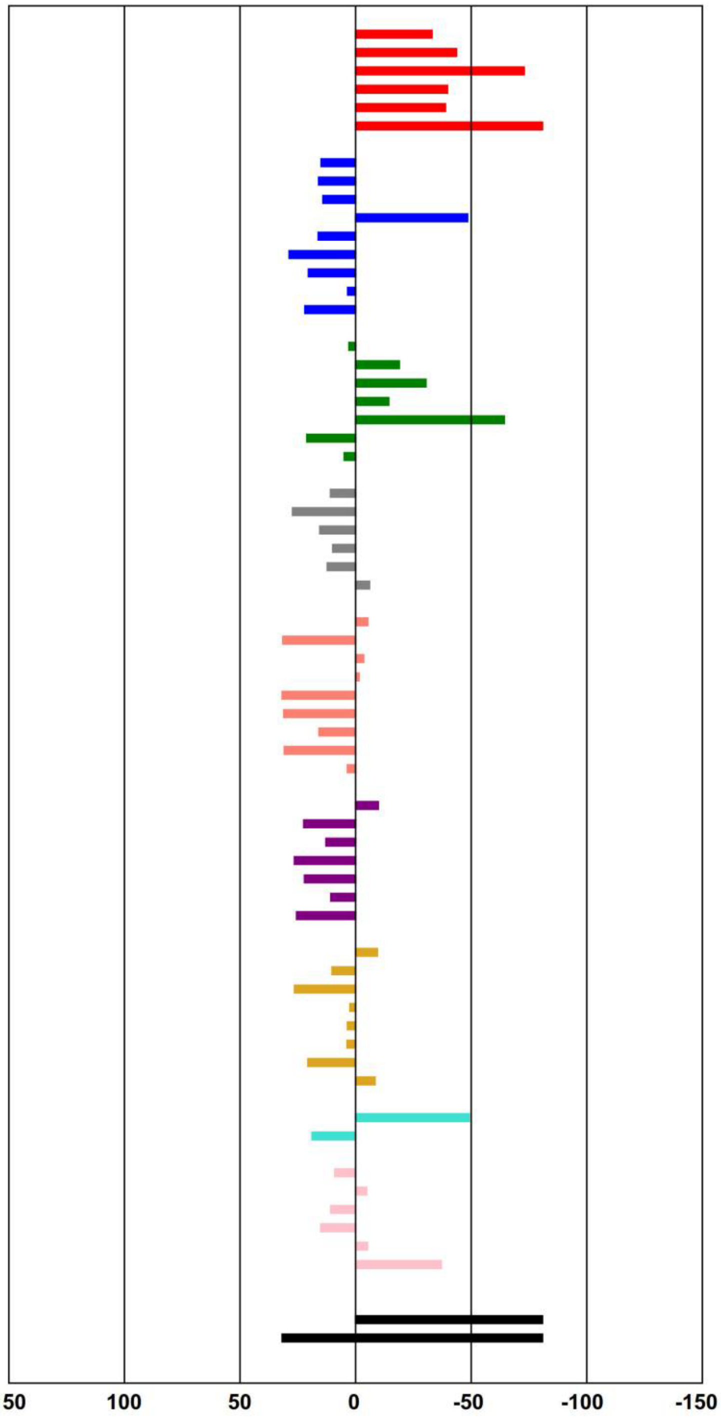

Figure S13. Anticancer screening data of compound 14 at single dose assay

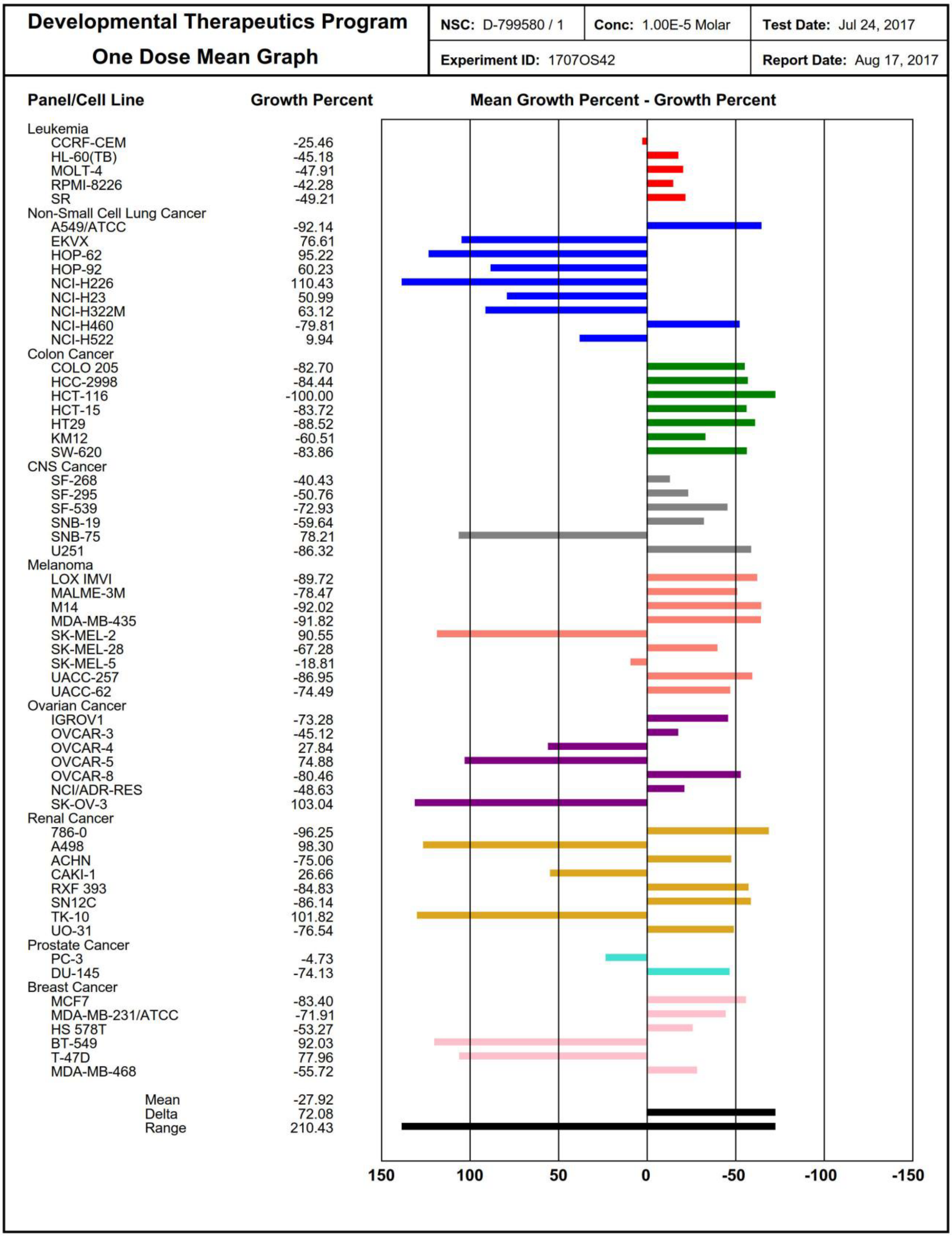

Figure S14. Anticancer screening data of compound 15 at single dose assay

Developmental Therapeutics Program One Dose Mean Graph	NSC: D-806835 / 1	Conc: 1.00E-5 Molar	Test Date: Aug 27, 2018
	Experiment ID: 1808OS00		Report Date: Sep 19, 2018

Panel/Cell Line	Growth Percent
**Leukemia**	
CCRF-CEM	34.52
HL-60(TB)	24.49
K-562	-20.00
MOLT-4	12.91
RPMI-8226	26.80
SR	-27.30
**Non-Small Cell Lung Cancer**	
A549/ATCC	34.65
EKVX	83.04
HOP-62	78.11
HOP-92	41.84
NCI-H226	81.83
NCI-H23	73.70
NCI-H322M	79.72
NCI-H460	-60.19
NCI-H522	61.98
**Colon Cancer**	
COLO 205	-66.51
HCC-2998	-12.34
HCT-116	-82.96
HCT-15	-50.92
HT29	-59.27
KM12	-1.94
SW-620	-71.33
**CNS Cancer**	
SF-268	69.61
SF-295	103.57
SF-539	74.80
SNB-19	85.73
SNB-75	86.37
U251	-81.12
**Melanoma**	
LOX IMVI	-87.74
MALME-3M	95.80
M14	80.69
MDA-MB-435	31.01
SK-MEL-2	102.23
SK-MEL-28	99.77
SK-MEL-5	91.54
UACC-257	97.43
UACC-62	89.70
**Ovarian Cancer**	
IGROV1	16.94
OVCAR-3	76.47
OVCAR-4	23.29
OVCAR-5	84.68
OVCAR-8	-13.72
NCI/ADR-RES	52.33
SK-OV-3	102.97
**Renal Cancer**	
786-0	34.70
A498	95.42
ACHN	48.58
CAKI-1	41.86
RXF 393	67.89
SN12C	46.43
TK-10	73.01
UO-31	60.19
**Prostate Cancer**	
PC-3	53.87
DU-145	37.91
**Breast Cancer**	
MCF7	23.80
MDA-MB-231/ATCC	45.63
HS 578T	85.28
BT-549	88.19
T-47D	1.19
MDA-MB-468	-35.07
Mean	37.20
Delta	124.94
Range	191.31

Mean Growth Percent - Growth Percent

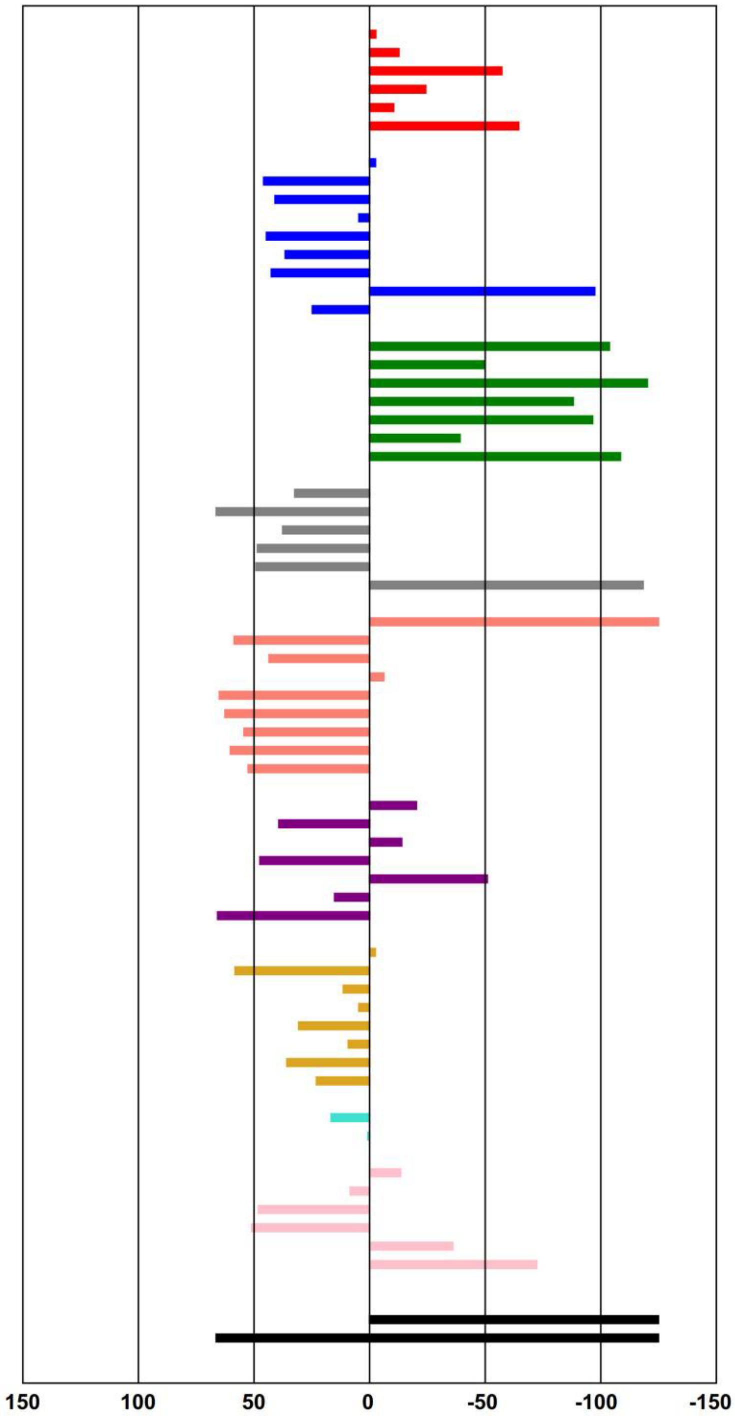

Figure S15. Anticancer screening data of compound 16 at single dose assay

Developmental Therapeutics Program	NSC: D-806829 / 1	Conc: 1.00E-5 Molar	Test Date: Aug 27, 2018
One Dose Mean Graph	Experiment ID: 1808OS00		Report Date: Sep 19, 2018

Panel/Cell Line	Growth Percent
**Leukemia**	
CCRF-CEM	0.85
HL-60(TB)	-58.40
K-562	-28.05
MOLT-4	-24.79
RPMI-8226	-66.03
SR	-30.93
**Non-Small Cell Lung Cancer**	
A549/ATCC	-38.22
EKVX	88.86
HOP-62	53.71
HOP-92	28.55
NCI-H226	66.78
NCI-H23	59.17
NCI-H322M	26.93
NCI-H460	-65.44
NCI-H522	-47.12
**Colon Cancer**	
COLO 205	-77.97
HCC-2998	-28.96
HCT-116	-91.45
HCT-15	-74.52
HT29	-69.23
KM12	-75.84
SW-620	-85.81
**CNS Cancer**	
SF-268	74.71
SF-295	102.41
SF-539	1.10
SNB-19	47.81
SNB-75	82.88
U251	-95.86
**Melanoma**	
LOX IMVI	-99.17
MALME-3M	15.09
M14	-64.90
MDA-MB-435	-38.22
SK-MEL-2	9.43
SK-MEL-28	96.70
SK-MEL-5	76.48
UACC-257	61.85
UACC-62	61.11
**Ovarian Cancer**	
IGROV1	-2.07
OVCAR-3	23.54
OVCAR-4	-40.54
OVCAR-5	34.76
OVCAR-8	-65.62
NCI/ADR-RES	-19.72
SK-OV-3	103.84
**Renal Cancer**	
786-0	-90.69
A498	103.72
ACHN	-46.63
CAKI-1	1.61
RXF 393	-7.17
SN12C	7.76
TK-10	68.56
UO-31	71.55
**Prostate Cancer**	
PC-3	43.55
DU-145	-1.23
**Breast Cancer**	
MCF7	-4.17
MDA-MB-231/ATCC	-43.73
HS 578T	83.36
BT-549	83.61
T-47D	-57.74
MDA-MB-468	-88.46
Mean	-.81
Delta	98.36
Range	203.01

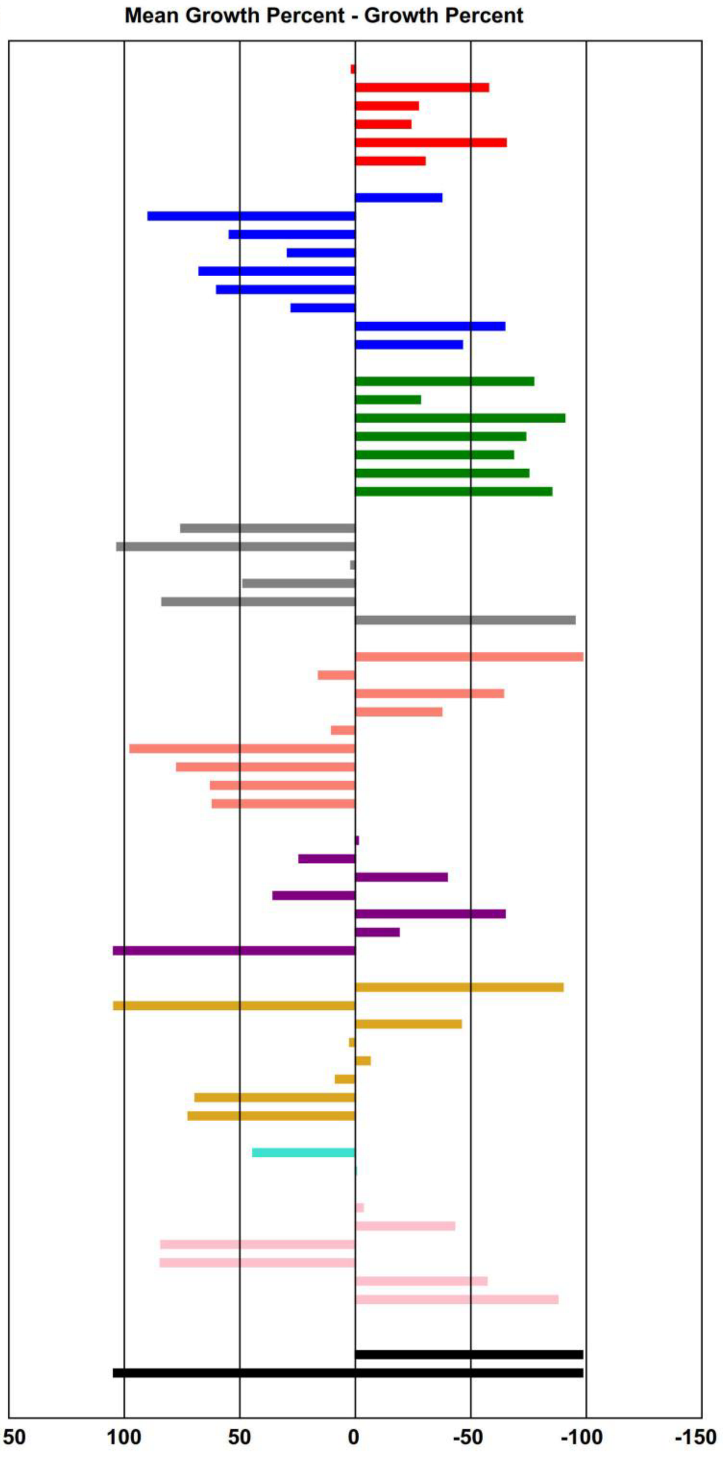

Figure S16. Anticancer screening data of compound 17 at single dose assay

Developmental Therapeutics Program One Dose Mean Graph	NSC: D-799582 / 1	Conc: 1.00E-5 Molar	Test Date: Jul 24, 2017
	Experiment ID: 1707OS42		Report Date: Aug 17, 2017

Panel/Cell Line	Growth Percent
**Leukemia**	
CCRF-CEM	93.13
HL-60(TB)	101.91
MOLT-4	66.97
RPMI-8226	95.01
SR	81.99
**Non-Small Cell Lung Cancer**	
A549/ATCC	92.00
EKVX	96.88
HOP-62	92.38
HOP-92	83.99
NCI-H226	97.86
NCI-H23	104.36
NCI-H322M	98.16
NCI-H460	99.60
NCI-H522	100.64
**Colon Cancer**	
COLO 205	94.17
HCC-2998	100.27
HCT-116	84.05
HCT-15	98.94
HT29	69.79
KM12	95.42
SW-620	98.64
**CNS Cancer**	
SF-268	85.30
SF-295	105.62
SF-539	91.23
SNB-19	99.35
SNB-75	78.07
U251	91.42
**Melanoma**	
LOX IMVI	97.13
MALME-3M	101.93
M14	92.64
MDA-MB-435	92.16
SK-MEL-2	99.51
SK-MEL-28	104.73
SK-MEL-5	98.32
UACC-257	113.98
UACC-62	97.99
**Ovarian Cancer**	
IGROV1	98.11
OVCAR-3	89.77
OVCAR-4	91.97
OVCAR-5	98.33
OVCAR-8	113.99
NCI/ADR-RES	105.43
SK-OV-3	110.01
**Renal Cancer**	
786-0	94.03
A498	100.24
ACHN	102.50
CAKI-1	81.57
RXF 393	91.38
SN12C	101.25
TK-10	100.39
UO-31	95.49
**Prostate Cancer**	
PC-3	83.47
DU-145	96.45
**Breast Cancer**	
MCF7	87.35
MDA-MB-231/ATCC	118.35
HS 578T	99.38
BT-549	102.81
T-47D	87.51
MDA-MB-468	86.90
Mean	95.46
Delta	28.49
Range	51.38

Mean Growth Percent - Growth Percent

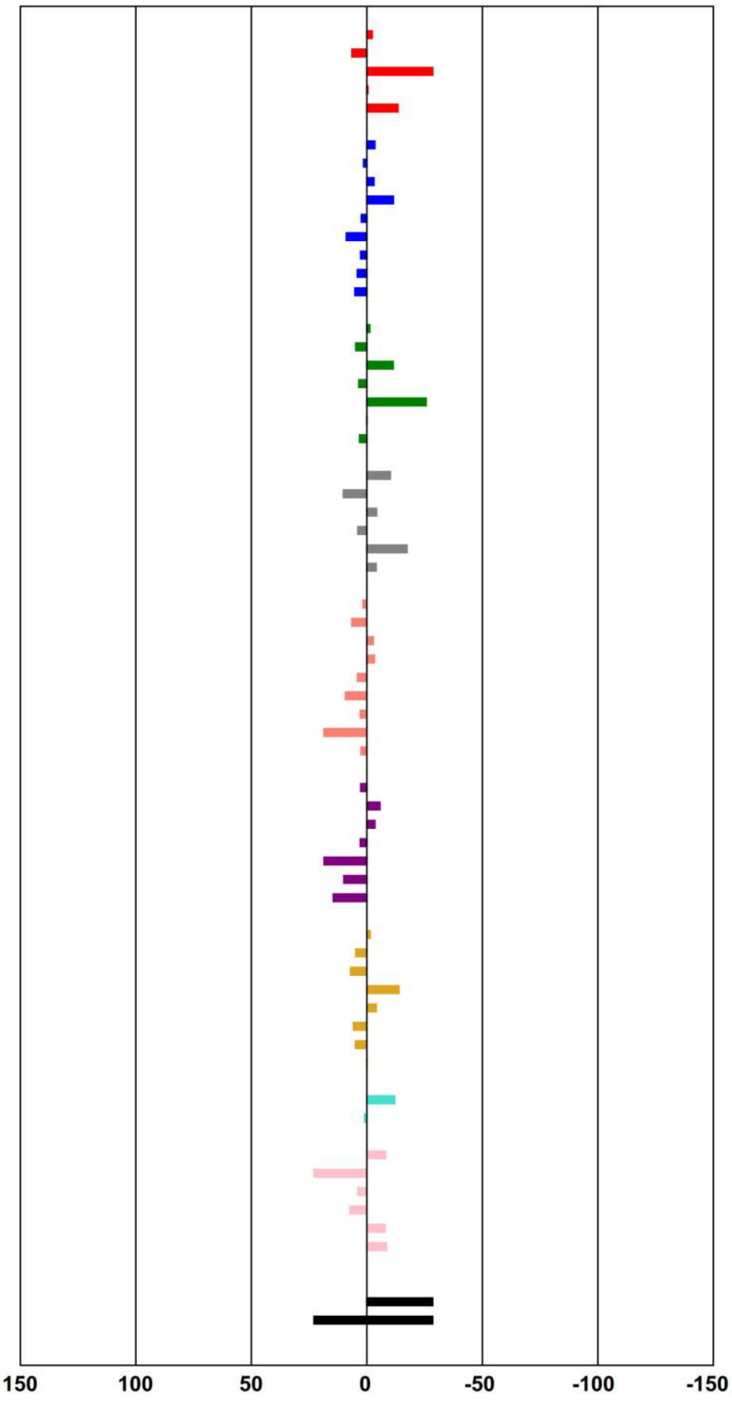

Figure S17. Anticancer screening data of compound 19 at single dose assay

Developmental Therapeutics Program	NSC: D-811982 / 1	Conc: 1.00E-5 Molar	Test Date: Jan 14, 2019
One Dose Mean Graph	Experiment ID: 1901OS55		Report Date: Feb 04, 2019

Panel/Cell Line	Growth Percent
Leukemia	
CCRF-CEM	0.72
HL-60(TB)	-45.20
K-562	1.75
MOLT-4	-10.56
RPMI-8226	-33.67
SR	-35.35
Non-Small Cell Lung Cancer	
A549/ATCC	-31.64
EKVX	74.13
HOP-62	-63.11
HOP-92	4.60
NCI-H226	84.53
NCI-H23	60.88
NCI-H322M	43.66
NCI-H460	-81.91
NCI-H522	-41.77
Colon Cancer	
COLO 205	-57.21
HCC-2998	-93.35
HCT-116	-57.79
HCT-15	-39.11
HT29	-44.55
KM12	-41.29
SW-620	-74.75
CNS Cancer	
SF-268	59.03
SF-295	77.15
SF-539	13.04
SNB-19	72.26
SNB-75	18.50
U251	-74.34
Melanoma	
LOX IMVI	-98.96
MALME-3M	-79.10
M14	-46.23
MDA-MB-435	-60.30
SK-MEL-2	-24.68
SK-MEL-28	-35.27
SK-MEL-5	67.13
UACC-257	-56.72
UACC-62	79.36
Ovarian Cancer	
IGROV1	30.01
OVCAR-3	20.07
OVCAR-4	5.39
OVCAR-5	60.57
OVCAR-8	-48.50
NCI/ADR-RES	14.06
SK-OV-3	14.09
Renal Cancer	
786-0	39.28
A498	72.54
ACHN	63.37
CAKI-1	22.37
RXF 393	21.74
SN12C	-4.38
TK-10	-68.29
UO-31	34.08
Prostate Cancer	
PC-3	10.96
DU-145	28.36
Breast Cancer	
MCF7	0.96
MDA-MB-231/ATCC	3.87
HS 578T	25.30
BT-549	55.32
T-47D	-34.06
MDA-MB-468	-54.70
Mean	-4.29
Delta	94.67
Range	183.49

Mean Growth Percent - Growth Percent

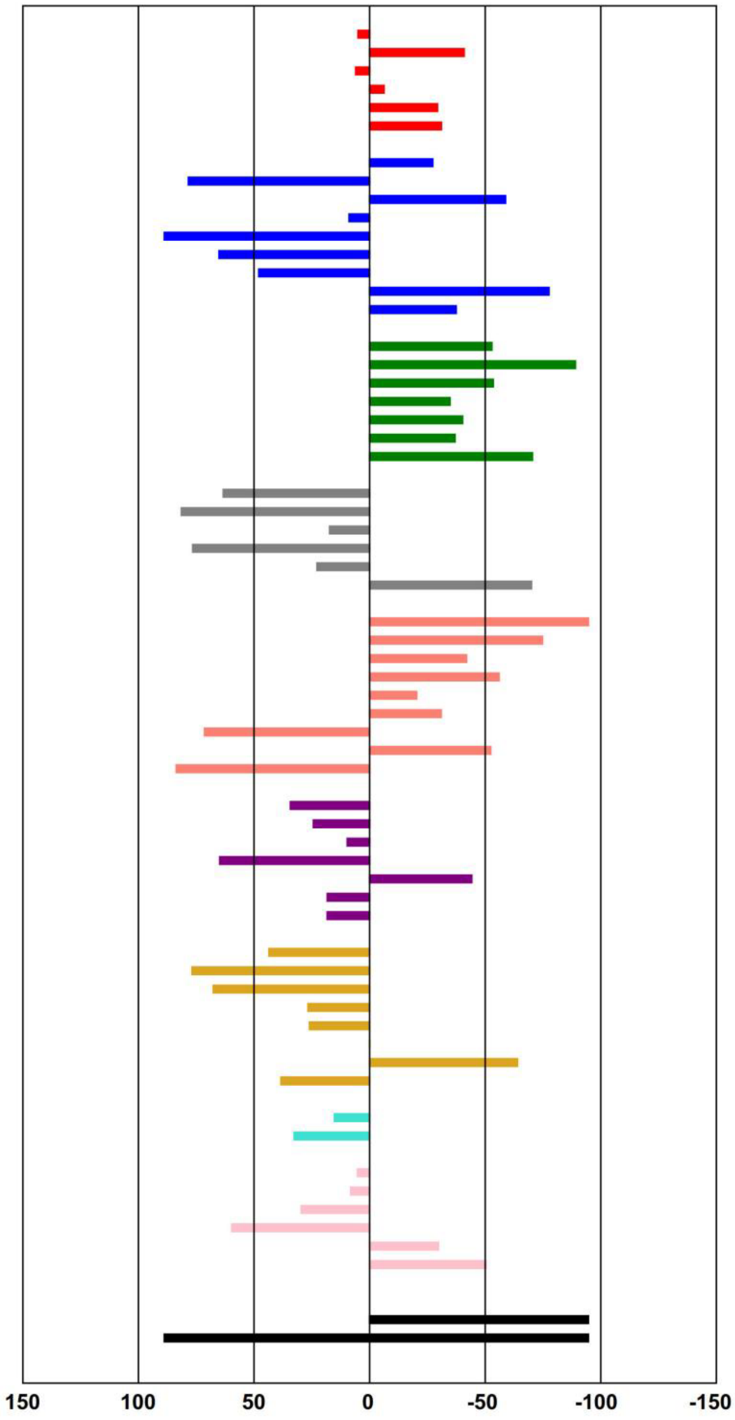

Figure S18. Anticancer screening data of compound 20 at single dose assay

Developmental Therapeutics Program One Dose Mean Graph	NSC: D-811985 / 1	Conc: 1.00E-5 Molar	Test Date: Jan 14, 2019
	Experiment ID: 1901OS55		Report Date: Feb 04, 2019

Panel/Cell Line	Growth Percent
**Leukemia**	
CCRF-CEM	0.79
HL-60(TB)	-57.96
K-562	0.27
MOLT-4	-10.47
RPMI-8226	-33.32
SR	-31.42
**Non-Small Cell Lung Cancer**	
A549/ATCC	-62.20
EKVX	42.52
HOP-62	-54.91
HOP-92	-52.89
NCI-H226	90.80
NCI-H23	-37.10
NCI-H322M	-14.29
NCI-H460	-86.19
NCI-H522	-43.10
**Colon Cancer**	
COLO 205	-64.41
HCC-2998	-92.03
HCT-116	-86.55
HCT-15	-82.03
HT29	-38.71
KM12	-71.73
SW-620	-80.83
**CNS Cancer**	
SF-268	-55.77
SF-295	73.26
SF-539	-89.96
SNB-19	34.30
SNB-75	-6.45
U251	-64.47
**Melanoma**	
LOX IMVI	-100.00
MALME-3M	-45.18
M14	-66.22
MDA-MB-435	-96.77
SK-MEL-2	-52.49
SK-MEL-28	-79.83
SK-MEL-5	17.55
UACC-257	-60.39
UACC-62	77.64
**Ovarian Cancer**	
IGROV1	6.46
OVCAR-3	-58.79
OVCAR-4	-55.44
OVCAR-5	19.15
OVCAR-8	-38.66
NCI/ADR-RES	-40.75
SK-OV-3	8.02
**Renal Cancer**	
786-0	-97.28
A498	68.30
ACHN	-94.94
CAKI-1	-81.62
RXF 393	-69.50
SN12C	-13.45
TK-10	-68.36
UO-31	47.22
**Prostate Cancer**	
PC-3	-33.98
DU-145	-72.65
**Breast Cancer**	
MCF7	-78.49
MDA-MB-231/ATCC	-75.70
HS 578T	-51.23
BT-549	80.04
T-47D	-43.69
MDA-MB-468	-62.10
Mean	-36.47
Delta	63.53
Range	190.80

Mean Growth Percent - Growth Percent

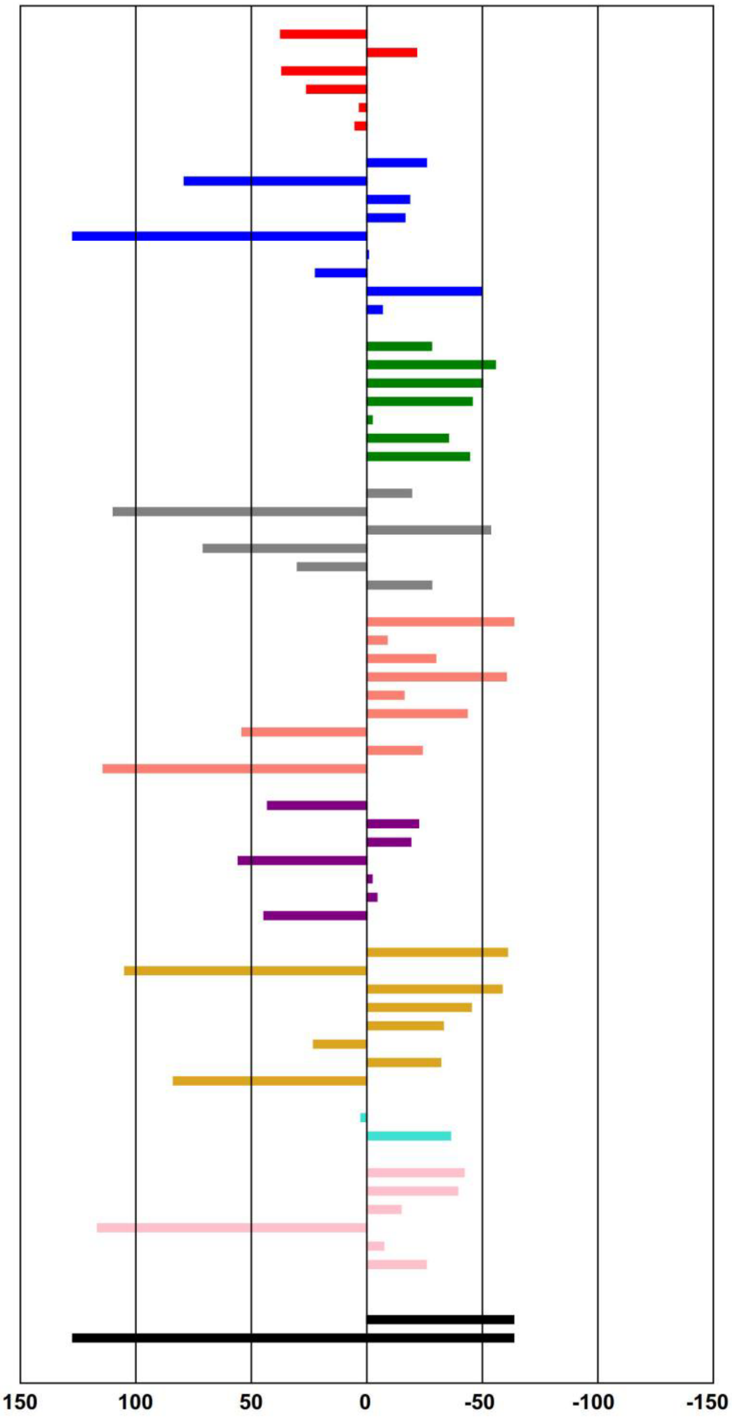

Figure S19. Anticancer screening data of compound 1 at a 5-dose assay

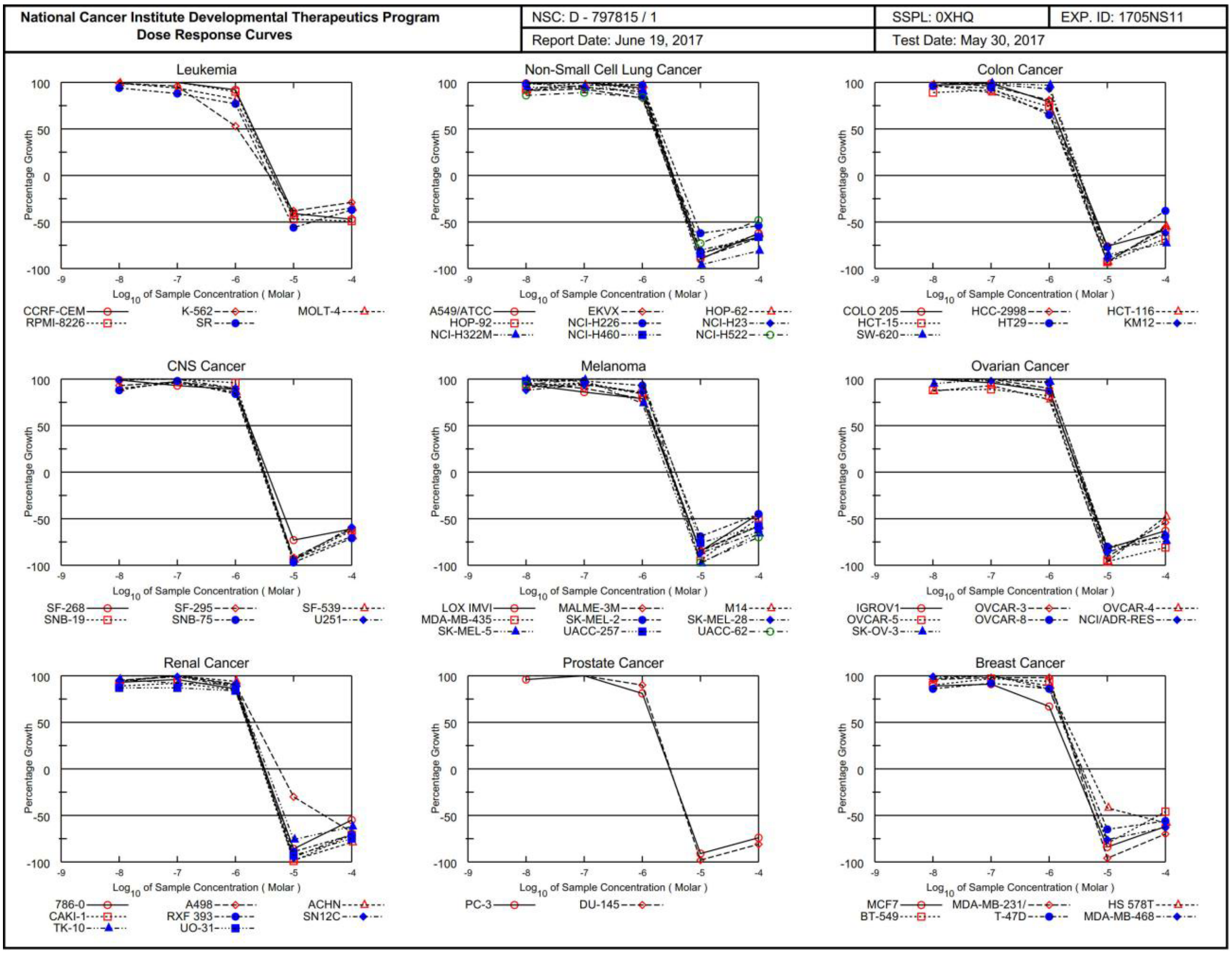

Figure S20. Anticancer screening data of compound 3 at a 5-dose assay

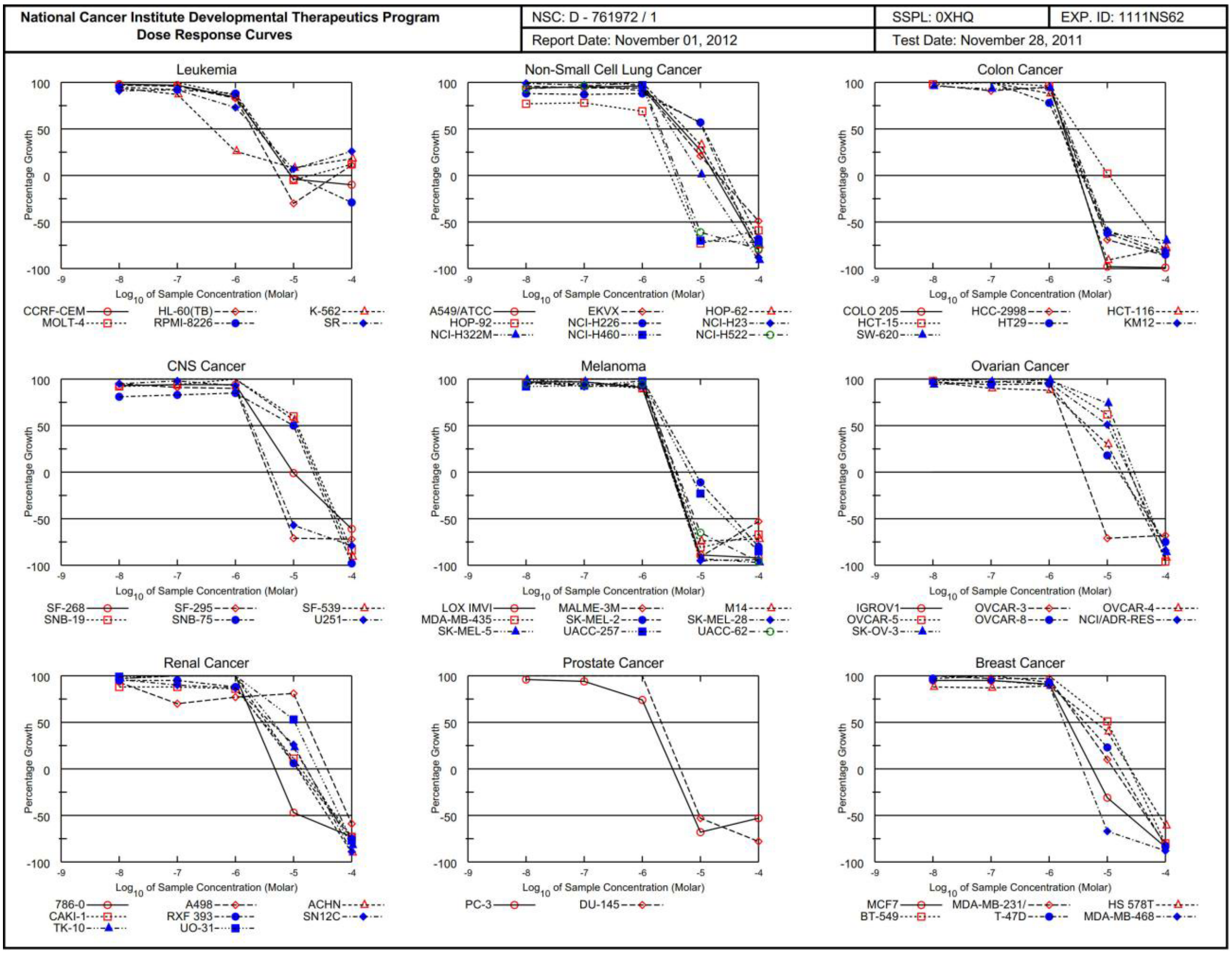

Figure S21. Anticancer screening data of compound 4 at a 5-dose assay

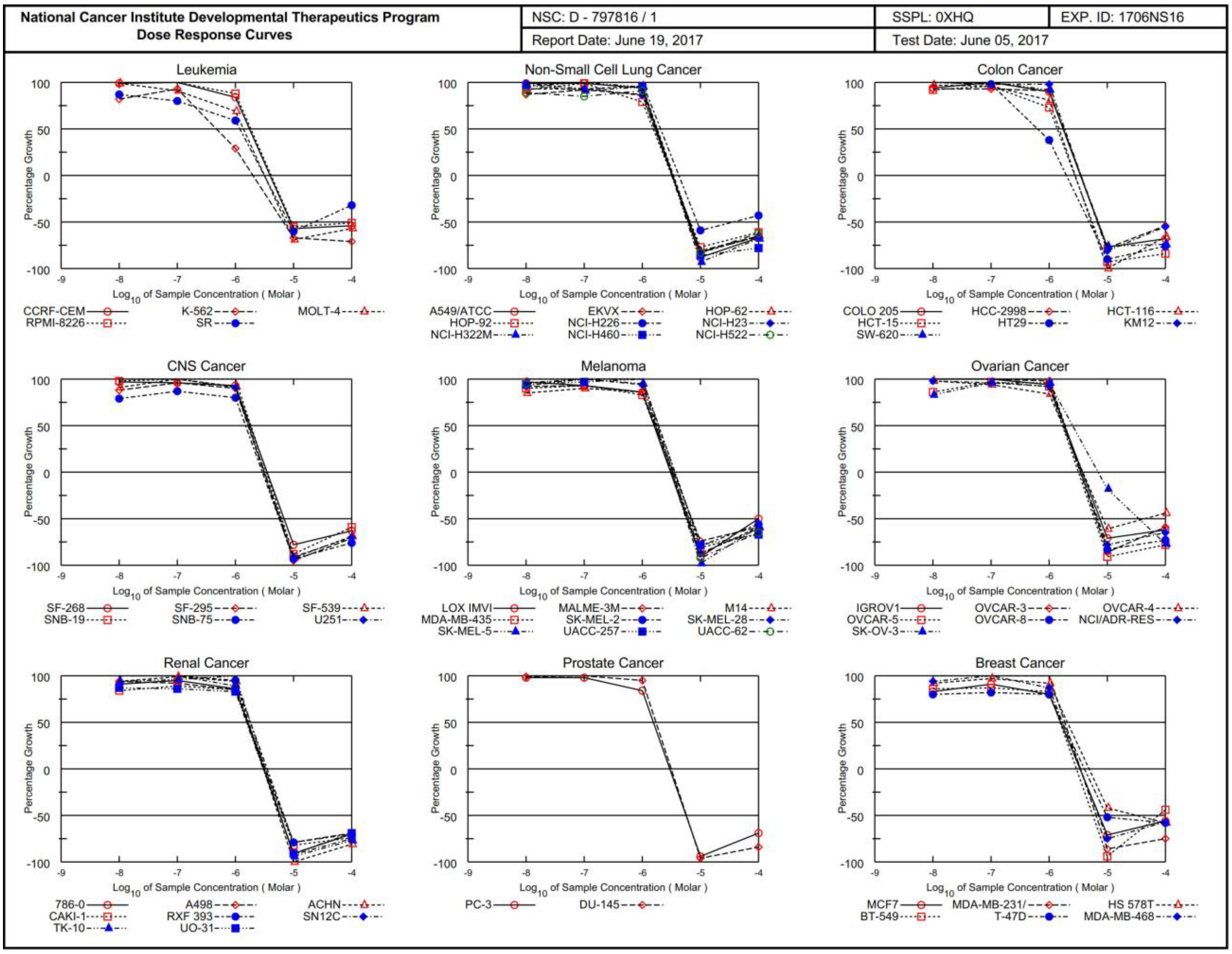

Figure S22. Anticancer screening data of compound 6 at a 5-dose assay

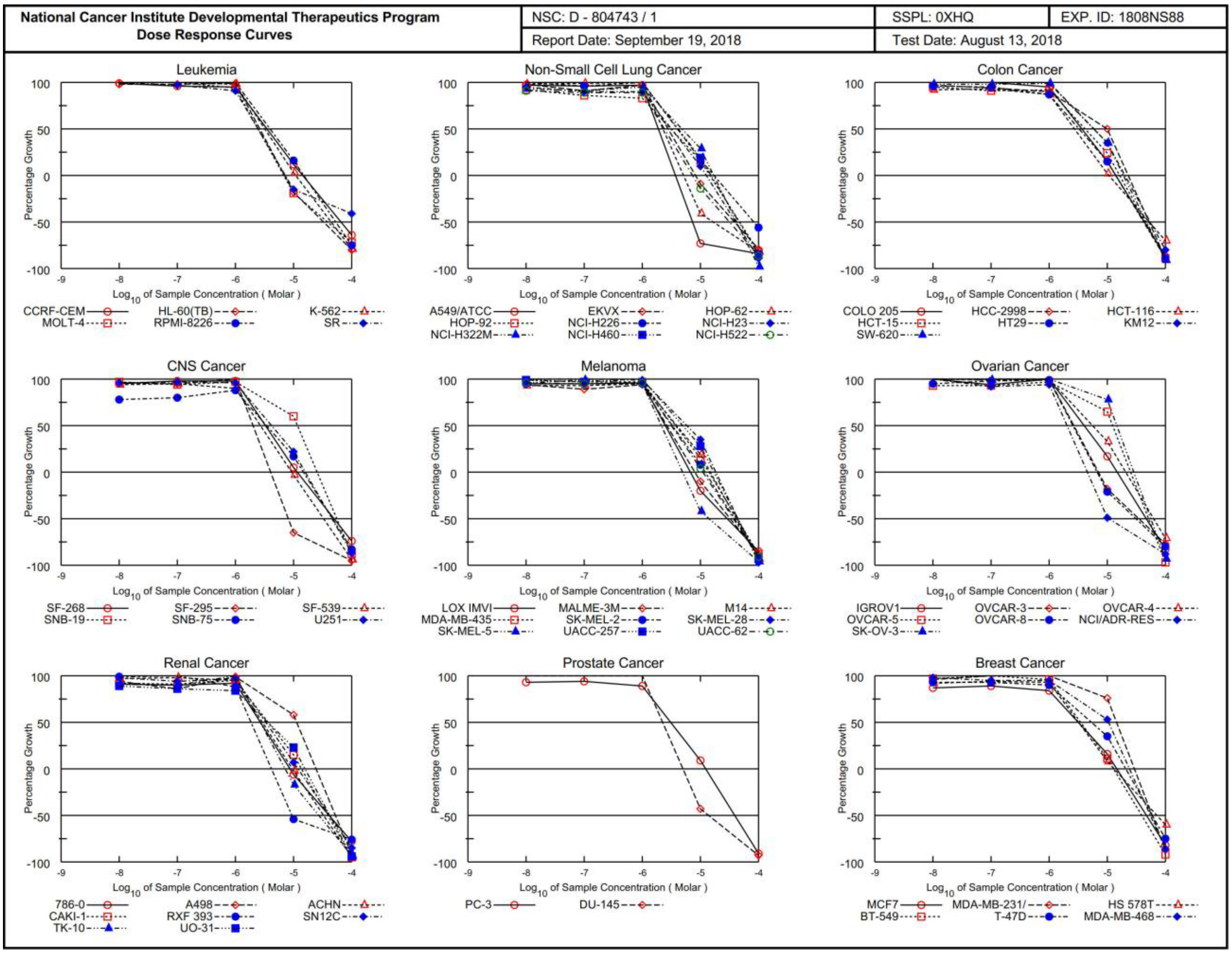

Figure S23. Anticancer screening data of compound 7 at a 5-dose assay

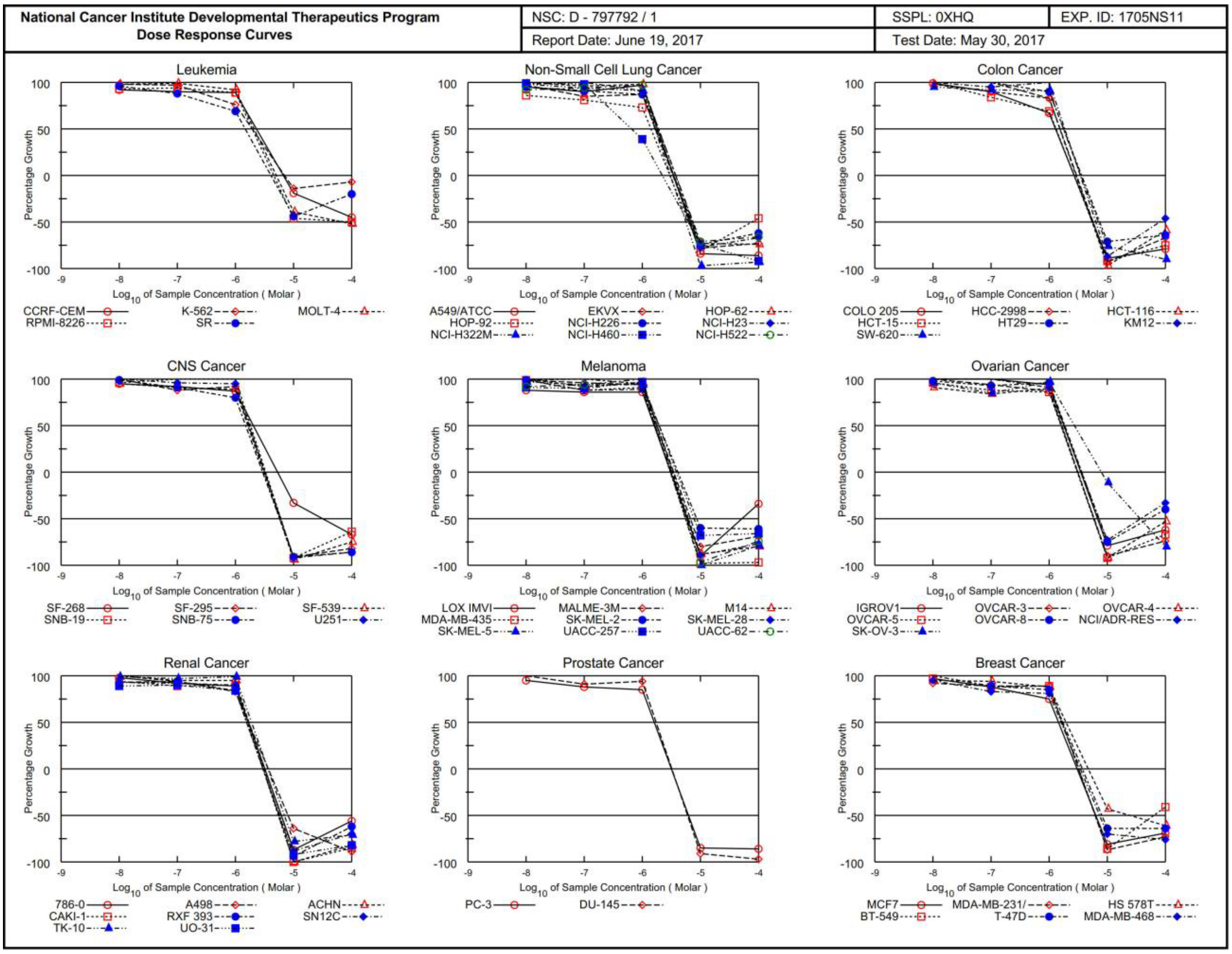

Figure S24. Anticancer screening data of compound 8 at a 5-dose assay

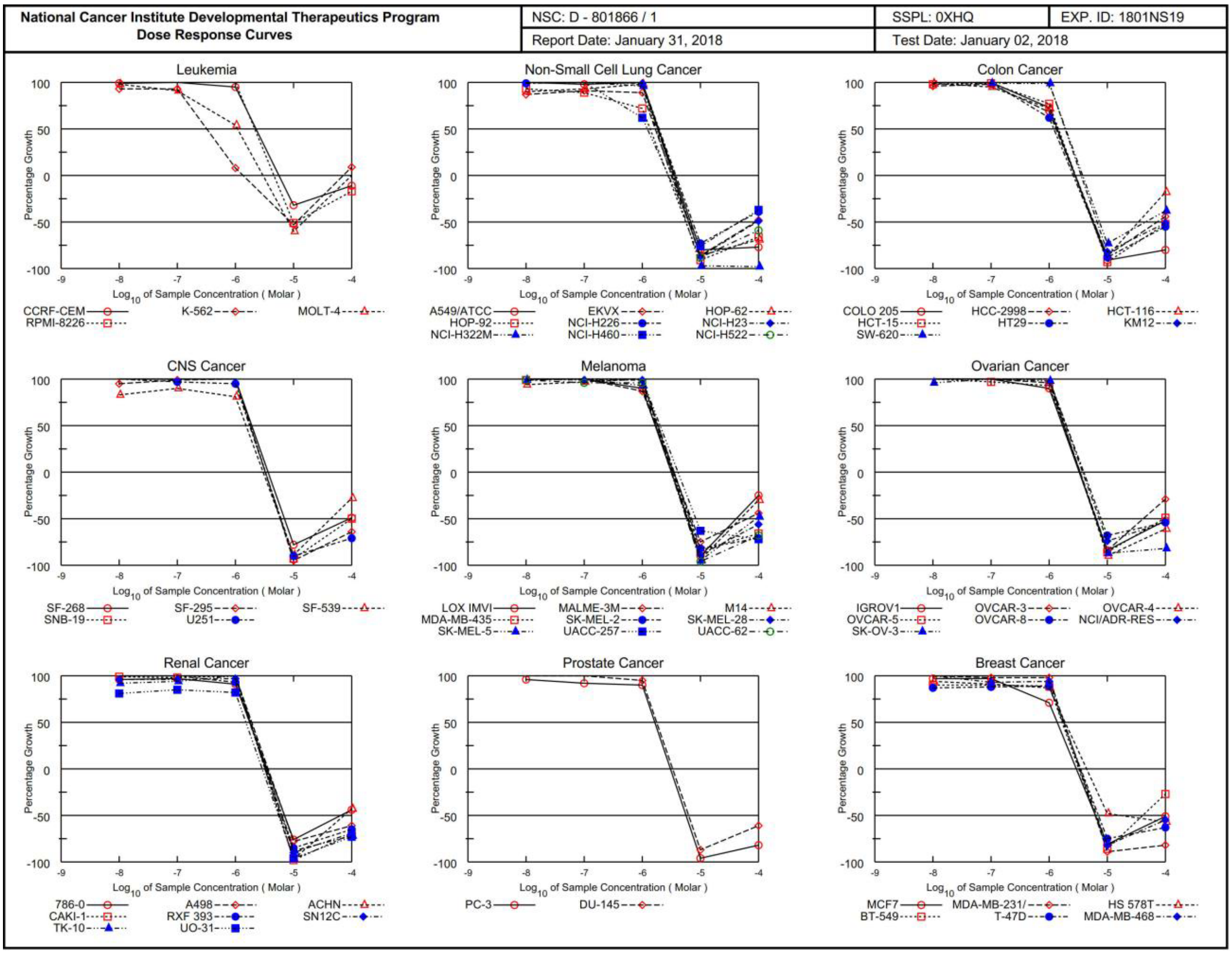

Figure S25. Anticancer screening data of compound 9 at a 5-dose assay

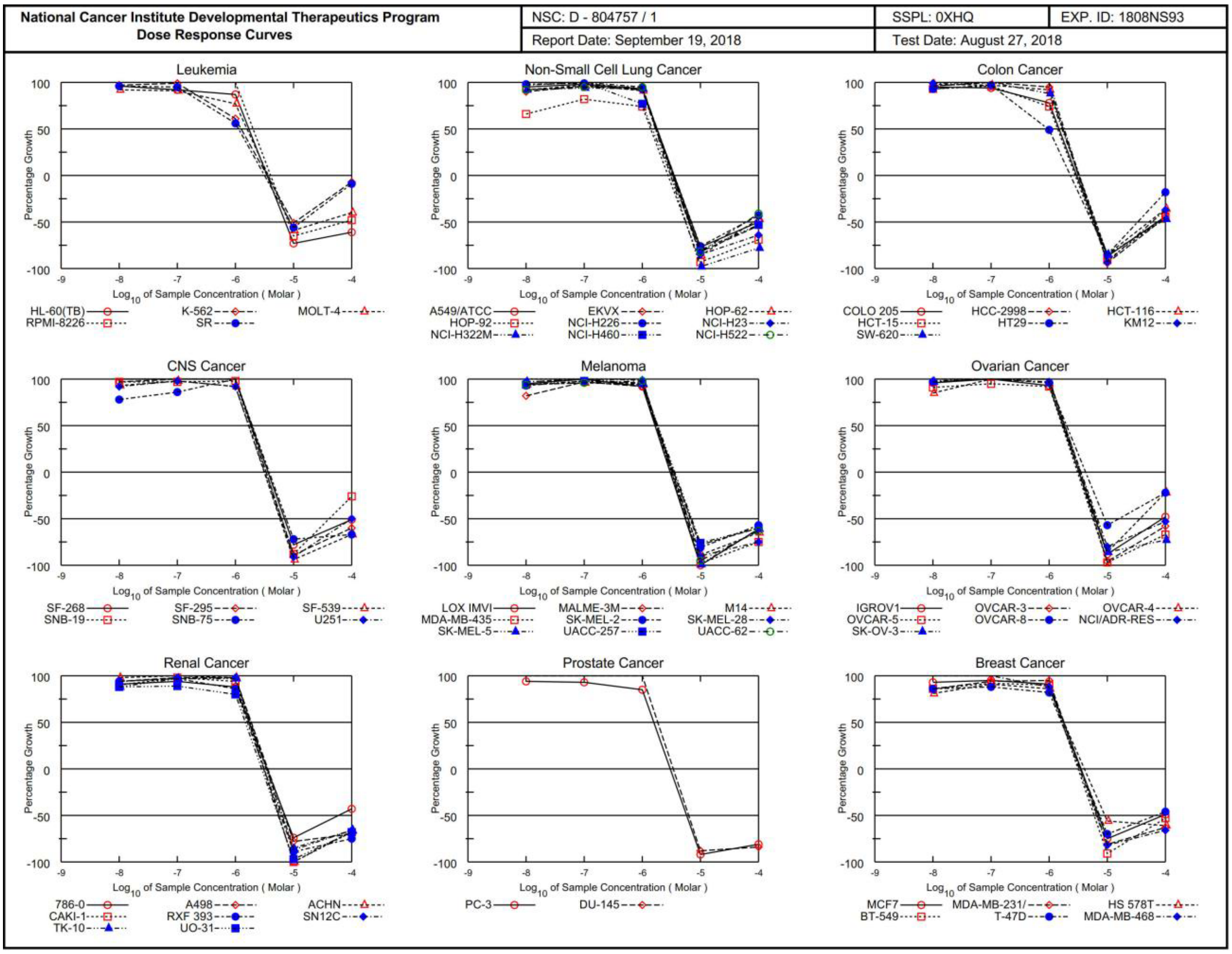

Figure S26. Anticancer screening data of compound 11 at a 5-dose assay

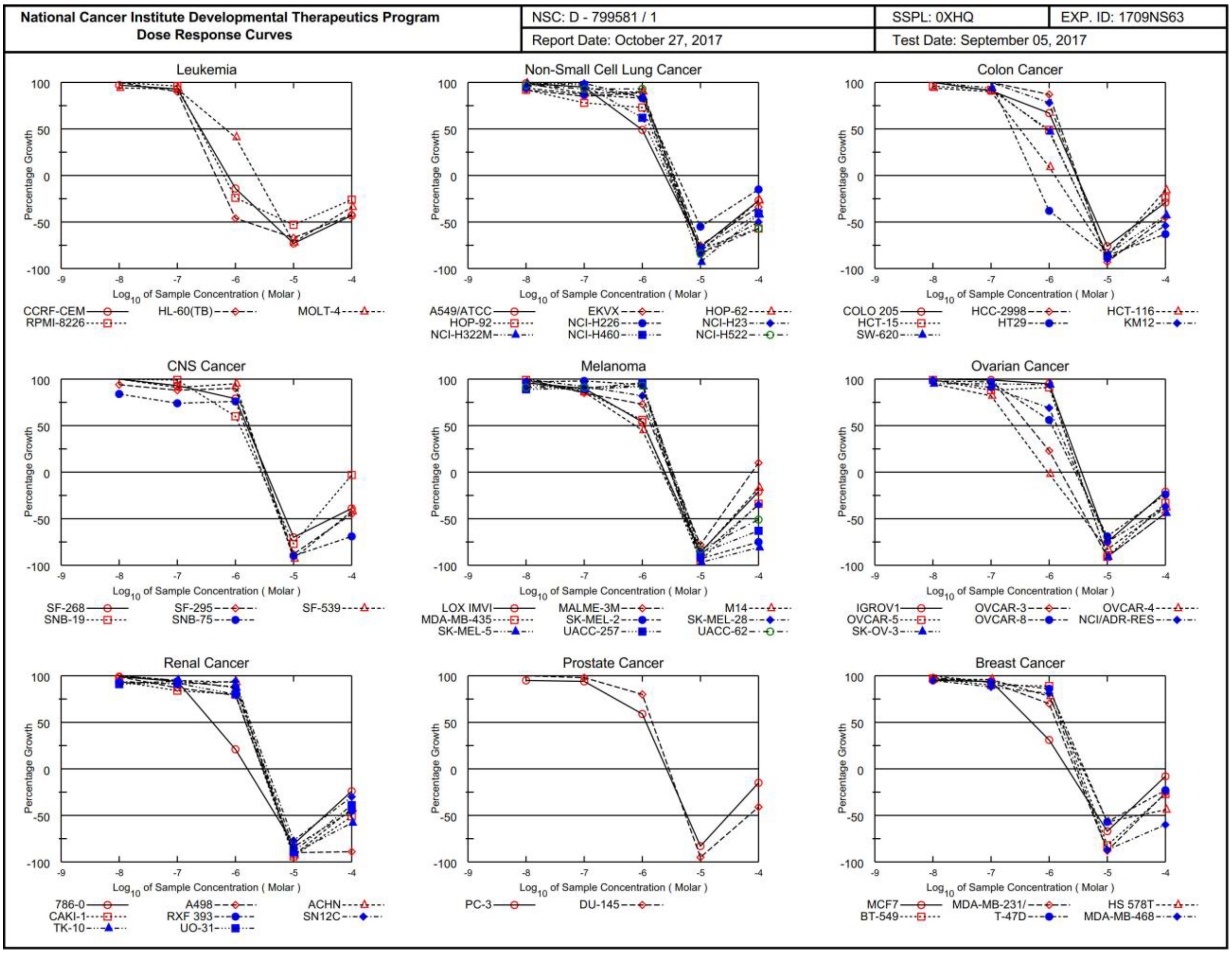

Figure S27. Anticancer screening data of compound 12 at a 5-dose assay

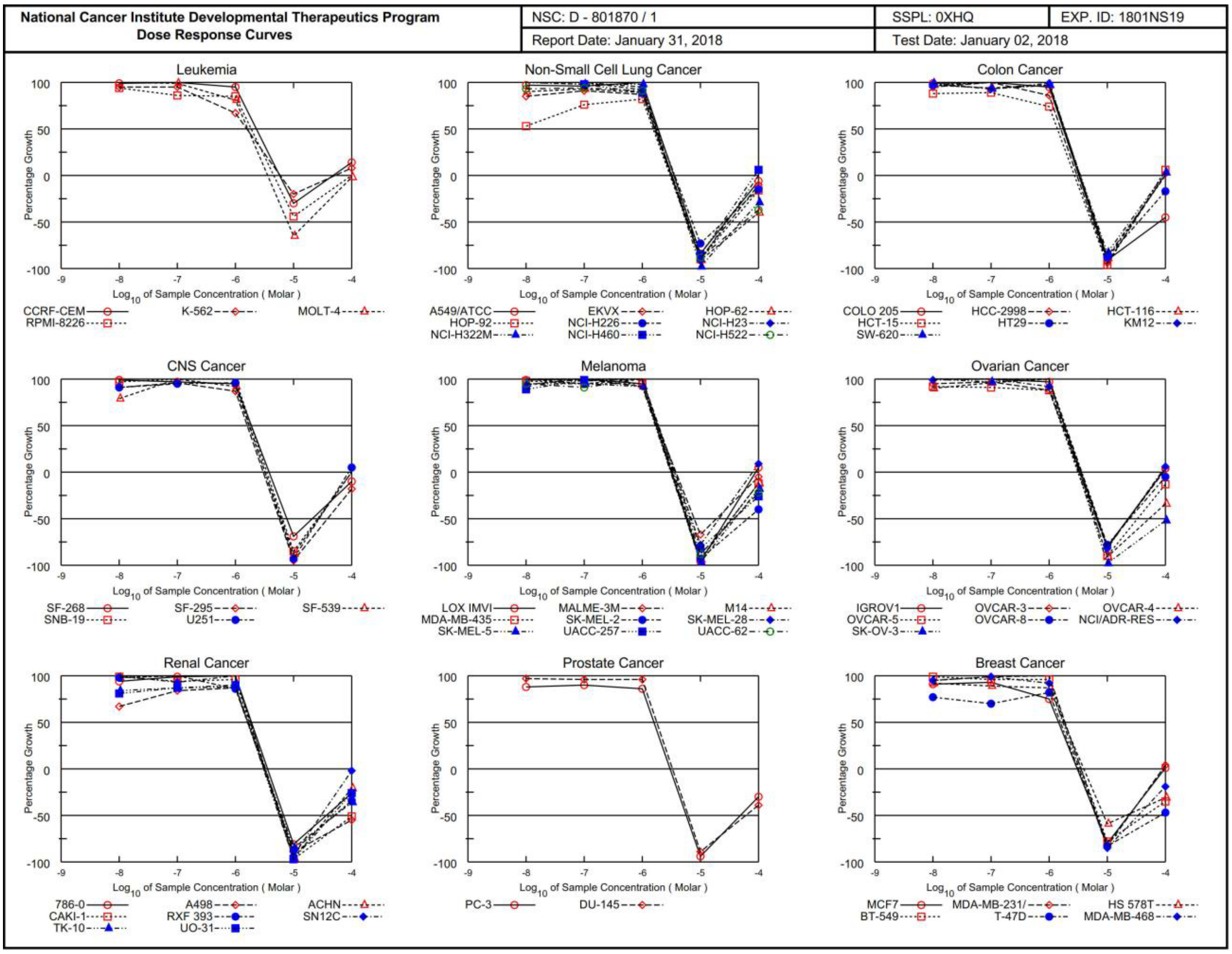

Figure S28. Anticancer screening data of compound 14 at a 5-dose assay

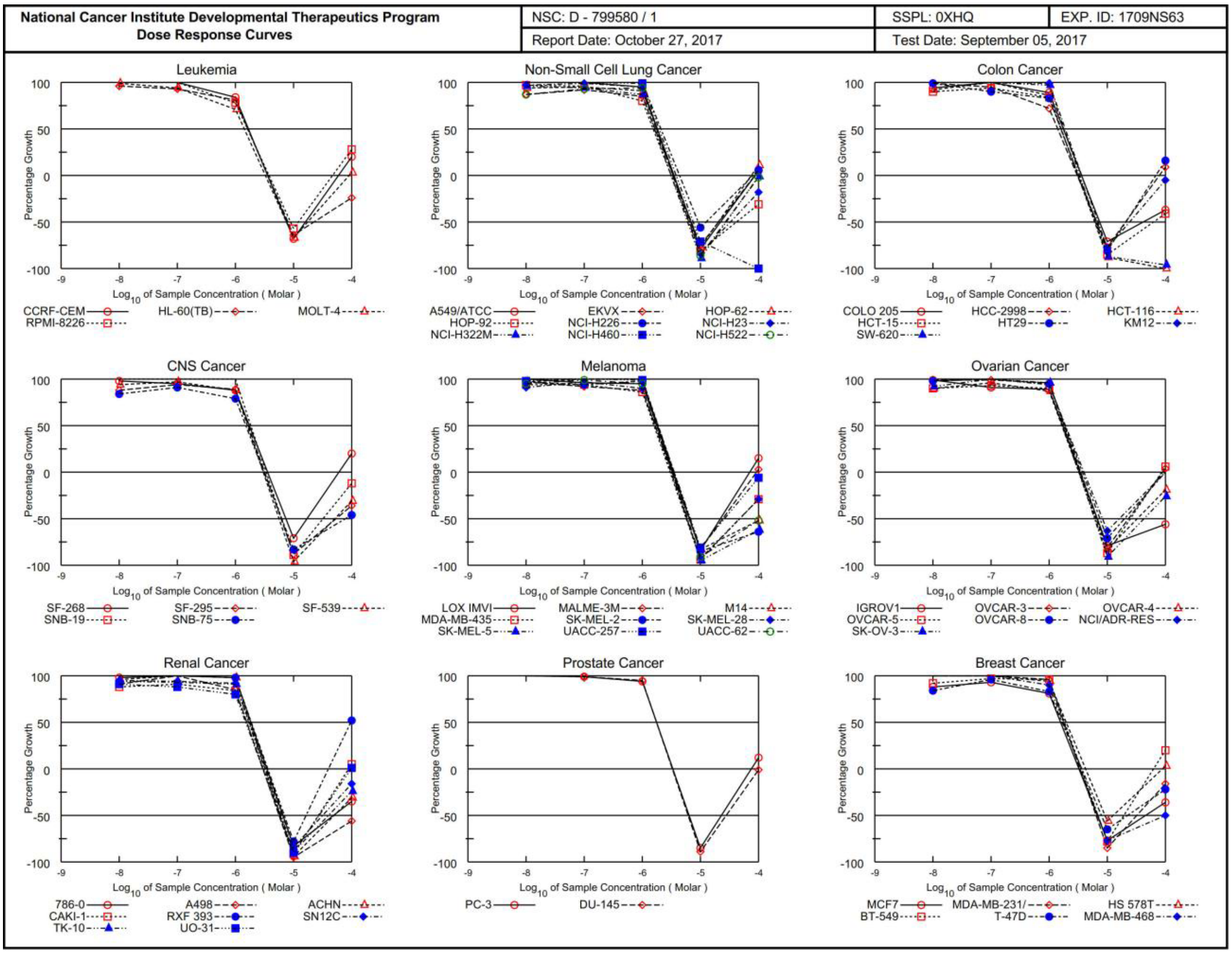

Figure S29. Anticancer screening data of compound 15 at a 5-dose assay

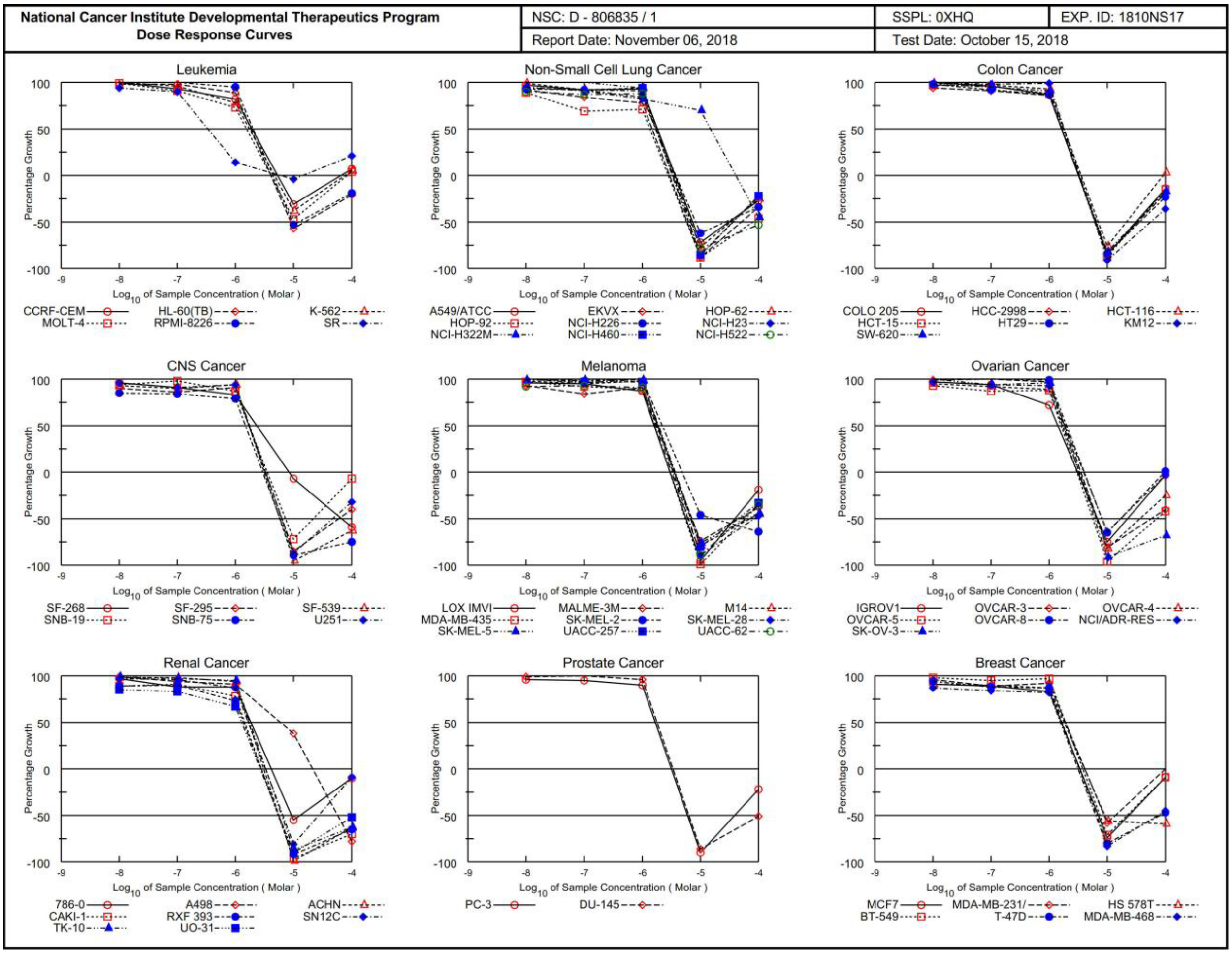

Figure S30. Anticancer screening data of compound 16 at a 5-dose assay

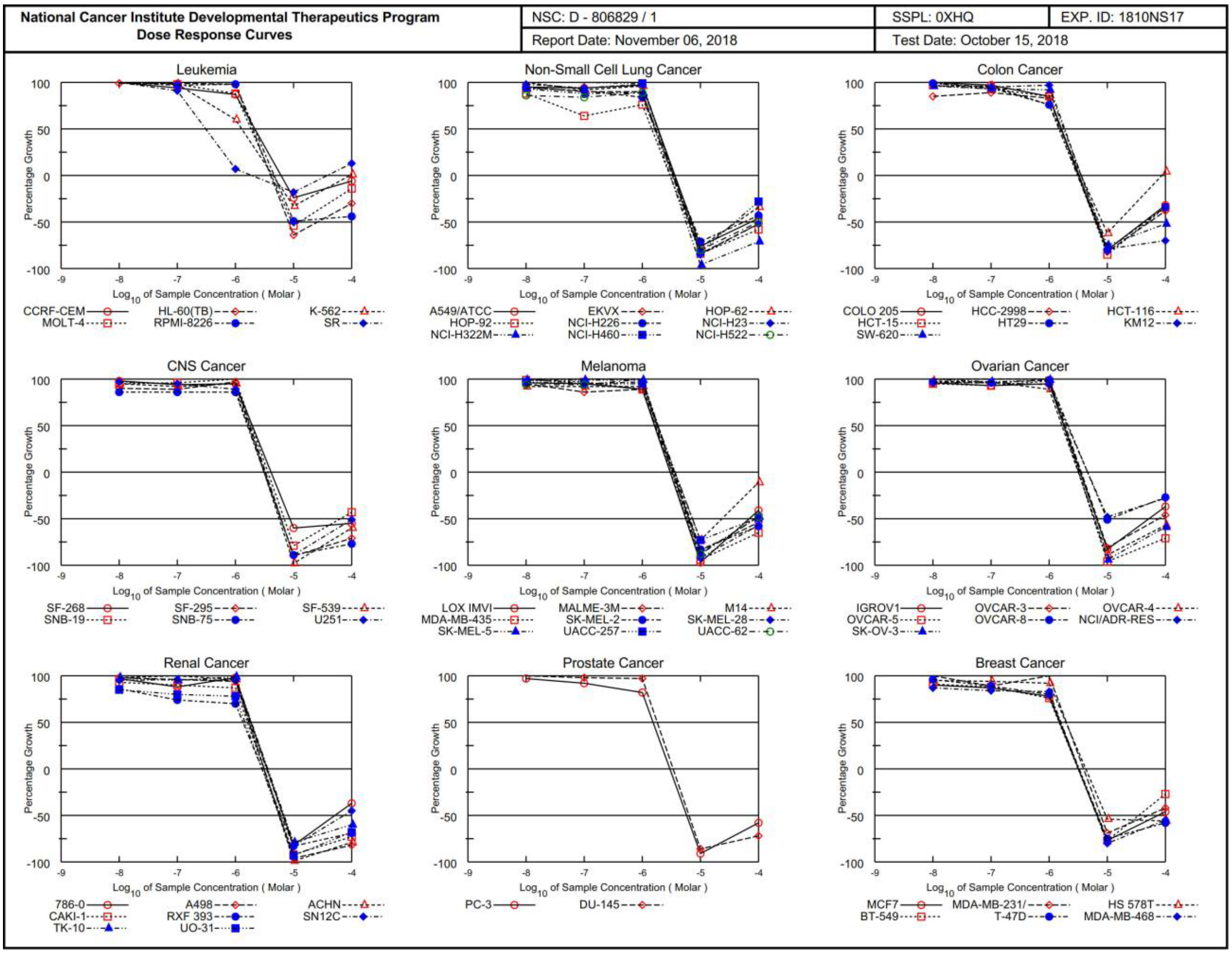

Figure S31. Anticancer screening data of compound 19 at a 5-dose assay

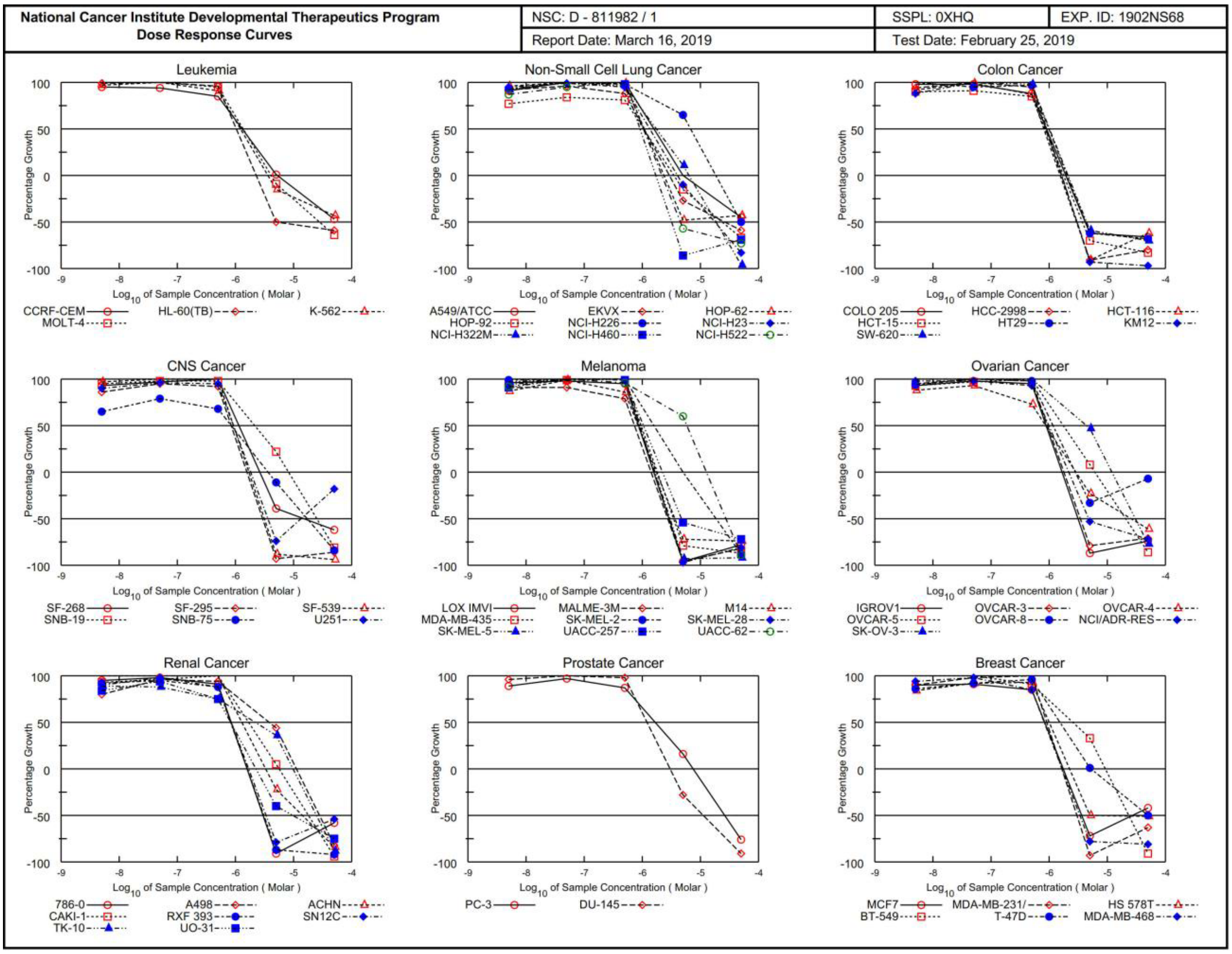

Figure S32. Anticancer screening data of compound 20 at a 5-dose assay

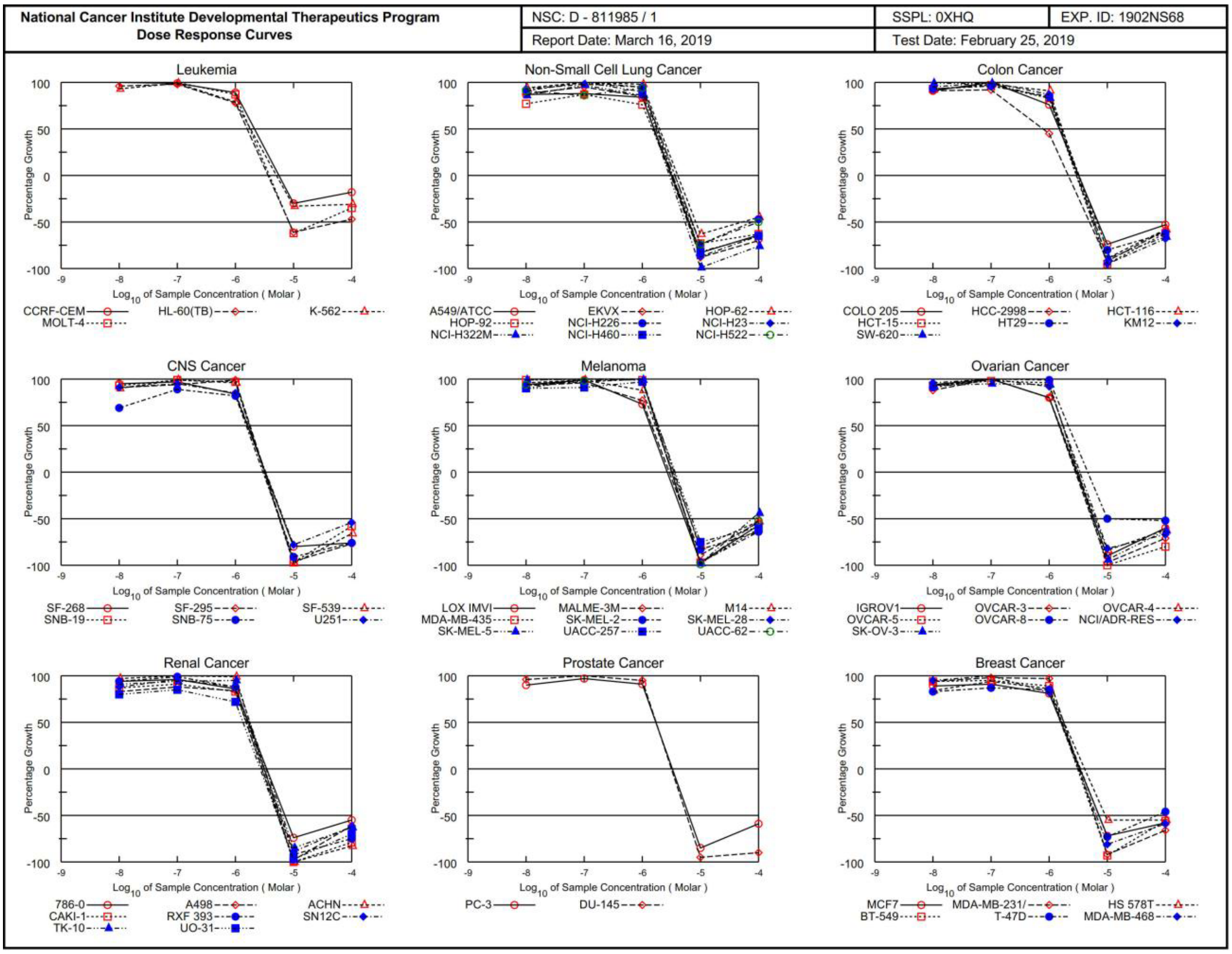

Table S1. Influence of compounds 1, 3, 4, 6, 7-9, 11, 12, 14-16, 19, 20 and standard drugs doxorubicin (DRB) and 5-fluorouracil (5-FU) on the growth of individual tumor cell panel.

Panel/cell line	GI_{50}[a], μM															
	1	3	4	6	7	8	9	11	12	14	15	16	19	20	DRB	5-FU
Leukemia																
CCRF-CEM	2.07	2.41	1.74	3.52	2.32	2.27	NT	0.25	2.28	1.67	1.93	2.14	1.31	2.12	0.08	9.97
HL-60(TB)	NT	1.95	NT	2.63	NT	NT	1.70	0.20	NT	1.64	1.85	1.98	1.03	1.58	0.19	2.30
K-562	1.08	0.40	0.47	3.18	1.96	0.32	1.25	NT	1.57	NT	1.75	1.28	1.22	1.80	NT	3.58
MOLT-4	1.79	2.50	1.37	2.46	2.10	1.09	1.59	0.67	1.62	1.42	1.55	1.84	1.35	1.78	0.03	0.35
RPMI-8226	1.94	2.71	1.85	3.91	1.94	2.26	2.06	0.24	1.86	1.60	2.03	2.12	NT	NT	0.08	0.04
SR	1.59	2.22	1.18	2.42	1.49	NT	1.14	NT	NT	NT	0.34	0.31	NT	NT	0.03	NT
MG_MID	1.69	2.03	1.32	3.02	1.96	1.48	1.55	0.34	1.83	1.58	1.57	1.61	1.23	1.82	0.08	3.25
NSC lung cancer																
A549/ATCC	1.73	4.62	1.74	1.89	1.81	2.02	1.79	0.96	1.87	1.81	1.83	1.86	1.59	1.60	0.06	0.18
EKVX	1.55	4.04	1.66	2.77	1.68	1.66	1.74	1.73	1.62	1.66	1.48	1.70	1.08	1.60	0.41	NT
HOP-62	1.81	5.89	1.81	2.30	1.91	1.82	1.70	1.71	1.65	1.76	1.81	1.84	1.08	1.98	0.07	0.39
HOP-92	1.73	1.36	1.52	3.10	1.41	1.37	1.39	1.42	1.54	1.54	1.35	1.45	1.04	1.48	0.10	77.9
NCI-H226	1.98	1.13	2.07	4.00	1.70	1.97	1.79	1.74	1.81	2.10	1.71	1.66	6.72	1.85	0.05	54.7
NCI-H23	1.71	1.11	1.65	3.15	1.76	1.83	1.78	1.60	1.75	1.76	1.70	1.82	1.34	1.77	0.15	0.33
NCI-H322M	1.63	3.69	1.73	4.78	1.64	1.73	1.68	1.55	1.76	1.72	14.9	1.62	1.74	1.57	NT	NT
NCI-H460	1.63	1.92	1.78	4.17	0.65	1.22	1.48	1.21	1.68	1.94	1.77	1.85	0.91	1.71	0.02	0.05
NCI-H522	1.65	2.04	1.71	2.38	1.90	1.93	1.80	1.75	1.70	1.75	1.69	1.70	1.04	1.83	0.03	7.27
MG_MID	1.71	2.87	1.74	3.17	1.49	1.73	1.68	1.52	1.71	1.78	3.14	1.72	1.84	1.71	0.11	20.12
Colon Cancer																
COLO 205	1.54	1.83	1.74	3.63	1.28	1.38	1.48	1.31	1.75	1.75	1.63	1.62	0.90	1.49	0.18	0.15
HCC-2998	1.51	1.89	1.75	10.1	1.54	1.31	1.74	1.61	1.58	1.40	1.63	1.58	0.87	0.79	0.26	0.05
HCT-116	1.29	1.64	1.49	2.73	1.52	1.37	1.73	0.31	1.79	1.60	1.80	1.54	0.81	1.69	0.08	0.22
HCT-15	1.39	3.06	1.39	4.21	1.31	1.44	1.40	0.97	1.38	1.59	1.65	1.61	0.84	1.53	6.46	0.11
HT29	1.27	1.59	0.63	3.25	1.78	1.20	0.94	0.24	1.93	1.59	1.64	1.48	0.99	1.63	0.12	0.17
KM12	1.73	2.07	1.84	6.09	1.86	1.93	1.92	1.47	1.84	1.89	1.82	1.86	0.91	1.60	0.27	0.21
SW-620	1.81	1.91	1.79	5.76	1.75	1.92	1.66	0.85	1.83	1.80	1.73	1.78	1.01	1.56	0.09	0.92
MG_MID	1.51	1.99	1.52	5.11	1.58	1.51	1.55	0.96	1.73	1.66	1.70	1.64	0.90	1.47	1.06	0.26
CNS cancer																
SF-268	1.74	2.90	1.79	3.27	2.04	1.91	1.97	1.56	1.88	1.73	2.32	1.95	1.22	1.61	0.10	1.62
SF-295	1.66	1.77	1.64	2.00	1.70	1.81	1.87	1.67	1.60	1.65	1.71	1.78	0.85	1.78	0.10	NT
SF-539	1.58	11.0	1.69	2.69	1.64	1.52	1.69	1.73	1.70	1.61	1.71	1.71	0.95	1.73	0.12	0.06
SNB-19	1.75	11.7	1.86	11.6	1.85	1.93	1.81	1.17	2.04	1.85	1.72	1.91	2.14	1.75	0.04	3.81
SNB-75	1.54	9.84	1.49	3.43	1.50	NT	1.98	1.43	NT	1.52	1.48	1.61	0.85	1.53	0.07	78.7
U251	1.65	1.94	1.67	4.17	1.74	1.76	1.69	NT	1.75	NT	1.74	1.66	0.92	1.64	0.04	0.92
MG_MID	1.65	6.53	1.69	4.53	1.58	1.79	1.83	1.51	1.79	1.67	1.78	1.77	1.15	1.67	0.08	17.02
Melanoma																
LOX IMVI	1.51	1.68	1.59	2.49	1.60	1.67	1.65	1.07	1.72	1.78	1.60	1.63	0.86	1.37	0.07	0.24
MALME-3M	1.50	2.02	1.68	2.64	1.79	1.70	1.77	1.42	1.84	1.66	1.77	1.68	0.73	1.46	0.12	0.05
M14	1.58	2.04	1.61	3.91	1.77	1.89	1.73	0.74	1.83	1.94	1.87	1.80	0.85	1.69	0.18	0.98
MDA-MB-435	1.56	1.71	1.59	3.76	1.65	1.87	1.85	1.10	1.73	1.58	1.61	1.64	0.95	1.78	0.25	0.07
SK-MEL-2	1.84	3.25	1.90	3.31	1.95	2.01	1.81	1.72	1.84	1.84	2.19	1.88	1.73	1.92	0.17	56.7
SK-MEL-28	1.62	1.70	1.75	5.87	1.66	1.81	1.81	1.55	2.15	1.66	1.84	1.77	0.95	1.82	0.21	1.03
SK-MEL-5	1.38	1.68	1.70	2.14	1.69	1.70	1.70	1.67	1.67	1.85	1.90	1.82	0.91	1.72	0.08	0.46
UACC-257	1.95	2.49	1.95	5.24	1.92	2.19	1.86	1.78	1.97	1.87	1.85	1.85	1.07	1.82	0.14	3.55
UACC-62	1.82	1.92	1.85	3.13	1.79	1.73	1.77	1.76	2.05	1.74	1.68	1.84	5.82	1.78	0.12	0.52
MG_MID	1.64	2.05	1.74	3.61	1.76	1.84	1.77	1.42	1.87	1.77	1.81	1.77	1.54	1.71	0.15	7.07
Ovarian Cancer																
IGROV1	1.65	NT	1.85	4.08	1.79	1.69	1.73	1.83	1.84	1.70	1.41	1.79	0.89	1.50	0.17	1.22
OVCAR-3	1.66	1.96	1.83	2.69	1.62	1.79	1.91	0.46	1.67	1.81	1.84	1.88	0.93	1.48	0.39	0.01
OVCAR-4	1.45	4.48	1.72	5.63	1.65	1.86	1.99	0.24	1.86	1.66	1.69	1.66	0.86	1.53	0.37	4.43
OVCAR-5	1.51	11.9	1.69	1.24	1.60	1.75	1.67	1.69	1.64	1.68	1.61	1.77	1.76	1.71	0.41	10.9
OVCAR-8	1.83	3.83	1.96	2.55	1.87	1.99	2.01	1.12	1.91	1.86	1.99	2.12	1.16	2.13	0.10	1.74
NCI/ADR-RES	1.63	10.2	1.76	2.02	1.78	1.98	1.84	1.35	1.74	1.91	1.89	2.05	0.99	1.74	7.16	0.31
SK-OV-3	1.83	14.1	2.54	14.5	2.73	1.81	1.89	1.73	1.87	1.77	1.76	1.79	4.42	1.72	0.22	21.8
MG_MID	1.65	7.75	1.91	4.67	1.86	1.84	1.86	1.20	1.79	1.77	1.74	1.86	1.57	1.69	1.26	5.77
Renal Cancer																
786-0	1.62	2.25	1.60	2.66	1.66	1.76	1.71	0.40	1.92	1.85	1.85	1.84	0.84	1.68	0.13	0.72
A498	2.17	16.7	1.63	11.4	1.68	2.03	1.87	1.49	1.63	1.56	5.99	1.71	3.83	1.54	0.10	0.35
ACHN	1.70	3.44	1.69	2.93	1.71	1.84	1.76	1.71	1.98	1.77	1.68	1.72	1.42	1.76	0.08	0.27
CAKI-1	1.55	3.05	1.62	3.70	1.62	1.81	1.69	1.49	1.74	1.56	1.45	1.61	1.69	1.52	0.95	0.07
RXF 393	1.68	2.88	1.81	1.84	1.56	1.74	1.61	1.67	1.62	NT	1.38	1.35	0.83	1.62	0.10	2.61

SN12C	1.62	4.02	1.64	3.34	1.68	1.79	1.83	1.81	1.88	1.73	1.67	1.79	0.84	1.64	0.07	0.49
TK-10	1.76	4.64	1.84	2.66	1.89	1.88	1.81	1.61	1.66	1.69	1.77	1.88	2.21	1.78	NT	1.12
UO-31	1.56	10.5	1.54	3.59	1.56	1.52	1.47	1.50	1.64	1.50	1.29	1.46	0.82	1.34	0.49	1.42
MG_MID	1.71	5.94	1.47	4.01	1.67	1.79	1.72	1.46	1.76	1.66	2.13	1.67	1.56	1.61	0.27	0.88
Prostate cancer																
PC-3	2.94	1.48	1.55	3.05	1.61	1.65	1.58	1.16	1.58	1.76	1.66	1.53	1.65	1.70	0.32	2.36
DU-145	3.02	2.30	1.72	2.25	1.74	1.77	1.98	1.48	1.77	1.76	1.79	1.81	1.21	1.72	0.11	0.36
MG_MID	2.98	1.89	1.64	2.65	1.67	1.71	1.78	1.32	1.67	1.76	1.72	1.67	1.43	1.71	0.21	1.36
Breast cancer																
MCF7	1.29	2.16	1.58	3.14	1.44	1.37	1.77	0.49	1.45	1.56	1.62	1.51	0.83	1.60	0.03	0.07
MDA-MB-31/ATCC	1.77	3.50	1.88	14.5	1.66	1.81	1.80	1.34	1.88	1.80	1.91	1.98	0.96	1.77	0.51	6.60
HS 578T	1.99	6.16	2.05	3.20	1.96	1.87	1.79	1.61	1.79	1.96	1.82	1.94	0.99	1.80	0.33	9.77
BT-549	1.79	1.01	1.55	3.46	1.68	1.68	1.66	1.69	1.84	1.84	1.90	1.48	2.56	1.64	0.23	10.6
T-47D	1.73	4.08	1.68	5.38	1.71	1.72	1.63	1.80	1.57	1.67	1.67	1.55	1.51	1.67	0.06	8.12
MDA-MB-468	1.66	1.79	1.70	10.4	1.61	1.78	1.68	1.52	1.74	1.74	1.56	1.59	0.82	1.58	0.05	NT
MG_MID	1.71	3.12	1.74	6.68	1.68	1.71	1.72	1.41	1.71	1.76	1.75	1.67	1.28	1.68	0.19	7.03
MG_MID_{60}	1.70	3.83	1.65	4.21	1.68	1.73	1.75	1.29	1.77	1.72	1.99	1.72	1.42	1.66	0.38	6.97

[a]GI_{50} was the drug concentration resulting in a 50% reduction in the net protein increase (as measured by SRB staining) in control cells during the drug incubation, determined at five concentration levels (100, 10, 1.0, 0.1 and 0.01 μM).

Table S2. Influence of compounds 1, 3, 4, 6, 7-9, 11, 12, 14-16, 19, 20 on the growth of individual tumor cell panel.

Panel/cell line	TGI[a], μM													
	1	3	4	6	7	8	9	11	12	14	15	16	19	20
Leukemia														
CCRF-CEM	4.91	8.96	3.93	14.5	6.73	5.63	NT	0.74	NT	NT	NT	6.06	5.26	5.56
HL-60(TB)	NT	NT	NT	7.02	NT	NT	3.50	0.46	NT	3.63	4.06	4.02	2.27	3.62
K-562	3.79	>100	2.02	10.8	7.00	NT	3.50	NT	NT	NT	NT	NT	3.63	5.04
MOLT-4	4.50	NT	3.15	6.80	5.04	NT	3.70	2.30	3.59	NT	NT	4.16	4.07	3.86
RPMI-8226	4.51	10.1	4.15	14.9	4.58	4.73	4.10	0.63	4.54	NT	4.42	4.63	NT	NT
SR	3.79	>100	3.13	7.17	4.12	NT	3.16	NT	NT	NT	NT	NT	NT	NT
MG_MID	4.30	54.76	3.28	10.19	5.49	5.18	3.59	1.03	4.06	3.63	4.24	4.72	3.81	4.52
NSC lung cancer														
A549/ATCC	3.24	17.8	3.28	3.71	3.43	3.73	3.52	2.45	3.50	NT	3.66	3.62	5.11	3.18
EKVX	3.03	20.1	3.29	8.27	3.37	3.21	3.38	3.49	3.11	NT	2.96	3.29	2.94	3.10
HOP-62	3.42	20.4	3.49	5.16	3.75	3.41	3.25	3.34	3.13	NT	3.56	3.59	2.36	4.04
HOP-92	3.33	30.7	3.19	14.6	2.96	2.77	2.77	3.01	3.01	3.14	2.80	2.97	3.46	3.21
NCI-H226	4.09	28.5	4.25	16.9	3.47	3.81	3.54	4.01	3.63	NT	3.77	3.47	18.3	3.66
NCI-H23	3.39	24.6	3.26	12.5	3.50	3.40	3.37	3.17	NT	3.39	3.27	3.42	4.00	3.32
NCI-H322M	3.03	10.3	3.19	16.9	3.03	3.14	3.07	2.79	3.16	3.11	4.05	3.01	6.38	2.91
NCI-H460	3.21	3.80	3.35	15.2	2.20	2.81	3.09	2.77	NT	3.82	3.37	3.50	1.71	3.33
NCI-H522	3.42	4.19	3.32	7.31	3.77	3.50	3.44	3.35	3.23	3.33	3.38	3.36	2.17	3.63
MG_MID	3.35	17.82	3.40	11.17	3.28	3.30	3.27	3.15	3.25	3.36	3.42	3.36	5.16	3.38
Colon Cancer														
COLO 205	3.24	3.25	3.46	13.9	2.68	2.79	2.97	2.94	3.24	3.59	3.19	3.26	1.94	3.23
HCC-2998	2.93	3.81	3.46	23.0	2.97	2.76	3.19	3.05	NT	NT	3.21	3.18	1.62	2.14
HCT-116	2.63	3.11	2.81	10.6	2.88	2.82	3.31	1.25	3.33	3.11	NT	NT	1.54	3.21
HCT-15	2.77	1.04	2.75	16.4	2.68	2.84	2.81	2.32	NT	3.15	3.20	3.17	1.76	2.92
HT29	2.85	3.62	1.97	13.8	3.63	2.58	2.31	0.54	3.50	NT	3.23	3.08	2.04	3.28
KM12	3.24	4.26	3.51	20.3	3.44	3.60	3.42	2.91	3.41	3.63	3.33	3.57	1.65	3.02
SW-620	3.41	4.00	3.54	18.8	3.49	3.75	3.23	2.26	NT	3.37	3.36	3.54	2.11	3.04
MG_MID	3.01	3.30	3.07	16.69	3.11	3.02	3.03	2.18	3.37	3.37	3.25	3.30	1.80	2.98
CNS cancer														
SF-268	3.54	9.77	3.50	11.6	5.31	3.64	3.71	3.39	3.79	NT	8.44	4.12	2.69	3.25
SF-295	3.13	3.62	3.06	4.04	3.19	3.26	3.42	3.21	3.01	3.17	3.30	3.29	1.58	3.21
SF-539	3.01	2.41	3.16	9.26	3.07	2.99	3.13	3.19	NT	3.01	3.15	3.11	1.73	3.13
SNB-19	3.20	2.61	3.43	25.3	3.35	3.44	3.36	2.72	3.68	3.40	3.54	3.62	8.11	3.17
SNB-75	2.91	2.17	2.90	14.8	2.94	NT	3.84	2.87	NT	3.09	2.94	3.10	3.62	2.97
U251	3.09	4.18	3.15	15.9	3.23	3.26	3.17	NT	NT	NT	3.29	3.17	1.82	3.32
MG_MID	3.15	4.13	3.20	13.48	3.51	3.31	3.44	3.08	3.49	3.17	4.11	3.40	3.26	3.18
Melanoma														
LOX IMVI	3.03	3.19	3.05	6.76	3.08	3.16	3.01	2.42	NT	NT	3.00	3.02	1.57	2.70
MALME-3M	3.05	3.56	3.44	8.01	3.47	3.46	3.48	NT	3.79	NT	3.56	3.26	1.42	2.91
M14	3.13	3.87	3.12	14.8	3.31	3.38	3.26	2.19	3.30	3.59	3.58	3.62	1.76	3.34
MDA-MB-435	2.95	3.36	3.21	14.0	3.02	3.49	3.28	2.36	3.19	3.00	2.97	3.07	1.81	3.20
SK-MEL-2	3.75	8.17	3.63	12.2	4.09	3.69	3.48	3.18	3.32	3.43	4.83	3.52	5.04	3.58
SK-MEL-28	3.15	3.14	3.30	18.4	3.18	3.35	3.32	3.03	NT	3.11	3.38	3.26	1.67	3.25
SK-MEL-5	2.70	3.14	3.09	4.94	3.06	3.14	3.08	3.07	3.08	3.32	3.68	3.39	1.65	3.19
UACC-257	3.73	6.47	3.70	17.0	3.89	4.29	3.63	3.34	3.65	3.54	3.54	3.68	2.21	3.65
UACC-62	3.25	3.93	3.35	10.9	3.21	3.16	3.22	3.39	3.64	3.23	3.20	3.39	12.6	3.17
MG_MID	3.19	4.31	3.32	11.89	3.37	3.46	3.30	2.87	3.42	3.32	3.53	3.36	3.30	3.22
Ovarian Cancer														
IGROV1	3.26	NT	3.72	14.8	3.48	3.28	3.27	3.61	NT	3.37	3.08	3.43	1.67	2.95
OVCAR-3	3.13	3.84	3.42	7.06	3.10	3.40	3.37	1.60	NT	NT	3.51	3.56	1.79	2.85
OVCAR-4	2.81	17.5	3.79	20.8	3.12	3.39	3.60	0.94	3.38	3.25	3.32	3.16	2.85	3.09
OVCAR-5	2.89	24.6	3.18	25.3	3.04	3.32	3.07	3.18	3.13	NT	3.01	3.21	6.15	3.06
OVCAR-8	3.51	15.6	3.61	6.66	3.66	3.94	4.25	2.80	3.65	NT	NT	4.57	2.80	4.61
NCI/ADR-RES	3.18	24.0	3.47	4.52	3.59	3.80	3.52	3.01	NT	NT	3.93	4.59	2.17	3.37
SK-OV-3	3.49	28.9	3.97	28.6	7.93	3.37	3.50	3.23	3.30	3.27	3.26	3.25	12.0	3.17
MG_MID	3.18	19.07	3.59	15.39	3.99	3.50	3.51	2.62	3.37	3.30	3.35	3.68	4.20	3.30
Renal Cancer														
786-0	3.16	4.84	3.06	8.59	3.21	3.51	3.48	1.60	3.59	3.48	4.14	3.51	1.58	3.44
A498	5.63	38.1	3.29	24.5	3.67	3.79	3.60	2.94	3.14	2.96	21.4	3.13	11.2	2.90
ACHN	3.09	11.5	3.06	9.56	3.08	3.26	3.14	3.16	3.45	3.24	3.07	3.10	3.40	3.14
CAKI-1	2.89	13.7	3.23	13.8	2.97	3.22	3.05	2.89	3.15	NT	2.82	3.06	5.58	2.85
RXF 393	3.18	11.7	3.51	4.15	2.98	3.32	3.10	3.27	3.13	NT	2.78	2.86	1.59	3.07

SN12C	3.03	16.8	3.11	12.0	3.20	3.31	3.44	3.55	3.37	3.33	3.30	3.41	1.67	3.15
TK-10	3.50	16.7	3.32	7.15	3.61	3.45	3.37	3.08	3.23	3.20	3.33	3.59	9.75	3.36
UO-31	2.99	25.5	2.99	15.7	3.00	2.89	2.82	2.96	3.04	NT	2.68	2.85	2.24	2.65
MG_MID	3.43	17.36	3.20	11.93	3.22	3.34	3.25	2.93	3.26	3.24	5.44	3.19	4.63	3.07
Prostate cancer														
PC-3	2.94	3.33	2.97	12.3	3.17	3.06	3.02	2.62	3.00	NT	3.16	2.98	7.48	3.28
DU-145	3.02	4.70	3.14	5.02	3.23	3.33	3.55	2.87	3.30	3.28	3.37	3.40	3.01	3.15
MG_MID	2.98	4.02	3.06	8.66	3.20	3.20	3.29	2.75	3.15	3.28	3.27	3.19	5.25	3.22
Breast cancer														
MCF7	2.77	5.54	3.39	14.5	3.01	2.92	3.54	2.06	NT	3.25	3.38	3.18	1.73	3.40
MDA-MB-31/ATCC	3.21	13.0	3.48	29.3	3.20	3.35	3.47	2.77	NT	3.40	4.12	3.91	1.71	3.27
HS 578T	4.79	24.7	4.86	13.2	4.69	4.39	4.04	3.78	3.94	NT	4.08	4.26	2.23	4.07
BT-549	3.46	24.5	2.96	12.7	3.24	3.23	3.14	3.32	3.56	NT	3.78	3.14	9.23	3.07
T-47D	3.71	16.3	4.03	20.9	3.69	3.47	3.46	4.02	3.15	3.64	3.31	3.26	5.15	3.45
MDA-MB-468	3.38	3.74	3.44	24.0	3.45	3.43	3.30	3.03	3.33	3.45	3.13	3.22	1.66	3.20
MG_MID	3.55	14.63	3.69	19.10	3.55	3.47	3.49	3.16	3.50	3.44	3.63	3.50	3.62	3.41
MG_MID_{60}	3.34	15.49	3.31	13.17	3.64	3.53	3.35	2.64	3.43	3.35	3.80	3.52	3.89	3.36

[a]TGI is a molar concentration of the compound leading to total inhibition

Table S3. Influence of compounds 1, 3, 4, 6, 7-9, 11, 12, 14-16, 19, 20 on the growth of individual tumor cell panel.

Panel/cell line	LC_{50}[a], μM													
	1	3	4	6	7	8	9	11	12	14	15	16	19	20
Leukemia														
CCRF-CEM	>100	>100	8.88	65.3	>100	>100	NT	NT	>100	NT	>100	>100	>50.0	>100
HL-60(TB)	NT	>100	NT	32.6	NT	NT	7.19	NT	NT	NT	NT	NT	5.00	NT
K-562	>100	>100	6.68	43.8	>100	NT	NT	NT	>100	NT	>100	>100	>50.0	>100
MOLT-4	>100	>100	7.25	38.2	74.8	NT	NT	NT	NT	NT	>100	NT	27.9	NT
RPMI-8226	>100	>100	9.27	52.9	96.8	NT	NT	NT	>100	NT	NT	>100	NT	NT
SR	NT	>100	NT	>100	>100	NT	NT	NT	NT	NT	>100	>100	NT	NT
MG_MID	>100	>100	8.02	55.47	94.32	>100	7.19	-	>100	-	>100	>100	33.22	>100
NSC lung cancer														
A549/ATCC	6.08	52.4	6.16	7.31	6.50	6.89	NT	NT	NT	NT	NT	NT	>50.0	6.34
EKVX	5.92	>100	6.53	38.4	6.77	NT	6.56	NT	NT	NT	NT	6.35	26.5	6.01
HOP-62	6.46	58.9	6.52	16.7	7.34	6.38	NT	NT	NT	NT	NT	NT	>50.0	NT
HOP-92	6.42	6.92	6.67	47.0	NT	5.61	5.52	6.42	NT	NT	NT	6.11	23.1	6.96
NCI-H226	8.43	71.8	NT	82.2	7.09	NT	NT	NT	NT	NT	NT	NT	49.9	NT
NCI-H23	6.68	54.5	6.47	40.0	6.95	NT	6.39	NT	NT	NT	NT	6.41	17.5	6.24
NCI-H322M	5.64	36.0	5.89	42.1	5.60	5.70	5.61	NT	NT	NT	>100	5.62	18.6	5.43
NCI-H460	6.30	7.56	6.30	46.2	6.12	NT	6.45	NT	NT	7.53	NT	NT	3.19	6.49
NCI-H522	NT	8.59	6.43	31.7	7.49	6.35	NT	6.40	NT	NT	6.78	6.64	4.54	NT
MG_MID	6.49	44.07	6.37	39.07	6.73	6.18	6.11	6.41	-	7.53	53.39	6.23	27.04	6.24
Colon Cancer														
COLO 205	6.82	5.77	6.86	43.0	5.61	5.64	NT	NT	NT	NT	NT	NT	4.17	6.96
HCC-2998	5.68	7.69	6.85	52.3	5.72	NT	NT	NT	NT	NT	NT	NT	3.02	4.97
HCT-116	5.38	5.92	5.31	52.9	5.47	NT	NT	NT	NT	6.05	NT	NT	2.93	6.08
HCT-15	5.52	42.3	5.52	45.4	5.47	5.59	NT	NT	NT	NT	NT	NT	3.69	5.56
HT29	NT	8.23	4.85	41.8	7.42	5.56	NT	1.75	NT	NT	NT	NT	4.22	6.59
KM12	6.09	8.78	6.68	55.1	NT	6.71	NT	5.78	NT	NT	NT	6.85	3.00	5.67
SW-620	6.44	8.35	7.03	47.0	6.95	NT	NT	NT	NT	6.31	NT	7.04	4.39	5.93
MG_MID	5.98	12.43	6.16	48.21	6.11	5.87	-	3.76	-	6.18	-	6.94	3.63	5.96
CNS cancer														
SF-268	7.19	65.6	6.84	49.6	31.5	NT	6.98	NT	NT	NT	67.9	8.68	14.8	6.56
SF-295	5.90	7.40	5.72	8.15	5.99	5.88	6.24	NT	NT	NT	NT	6.08	2.94	5.79
SF-539	5.72	52.7	5.90	33.0	5.78	NT	5.80	NT	NT	NT	5.79	5.66	3.17	5.67
SNB-19	5.86	58.1	6.31	55.2	6.07	NT	NT	NT	NT	NT	NT	NT	25.0	5.74
SNB-75	5.50	47.1	5.63	46.5	5.76	NT	7.44	5.72	NT	NT	5.85	5.97	17.1	5.77
U251	5.76	9.00	5.94	45.5	5.98	6.07	NT	NT	NT	NT	NT	6.07	NT	6.72
MG_MID	5.98	39.98	6.06	39.66	10.18	5.97	6.61	5.72	-	-	26.51	6.49	12.60	6.04
Melanoma														
LOX IMVI	NT	6.05	5.84	29.0	NT	NT	5.49	NT	NT	NT	NT	NT	2.86	5.31
MALME-3M	6.18	6.27	7.08	32.5	6.72	NT	6.84	NT	NT	NT	NT	6.34	2.74	5.82
M14	6.19	7.34	6.04	41.5	6.19	NT	6.12	NT	NT	6.65	NT	NT	3.64	6.62
MDA-MB-435	NT	6.59	6.49	43.0	5.55	6.51	5.81	NT	NT	NT	NT	5.75	3.45	5.76
SK-MEL-2	NT	36.7	6.91	38.9	8.57	6.80	6.69	5.87	NT	6.42	16.4	6.58	18.5	6.66
SK-MEL-28	6.13	5.78	6.22	44.0	6.07	6.18	6.11	NT	NT	NT	NT	5.97	2.94	5.80
SK-MEL-5	5.28	5.86	5.62	14.0	5.53	NT	5.56	5.64	NT	5.94	NT	NT	3.00	NT
UACC-257	7.14	27.6	7.01	43.8	7.77	8.38	7.09	6.25	NT	NT	NT	NT	4.68	7.14
UACC-62	5.80	8.07	6.06	37.0	5.77	5.76	5.85	6.52	NT	6.00	NT	NT	27.3	5.66
MG_MID	6.12	12.25	6.36	35.96	6.52	6.73	6.17	6.07	-	6.25	16.4	6.16	7.68	6.09
Ovarian Cancer														
IGROV1	6.44	NT	7.45	46.6	6.78	6.35	NT	NT	NT	6.69	NT	NT	3.14	5.80
OVCAR-3	5.91	7.52	6.39	34.8	5.94	NT	5.93	NT	NT	NT	NT	NT	3.44	5.46
OVCAR-4	NT	45.2	NT	63.1	5.88	6.17	NT	NT	NT	NT	NT	6.02	25.0	6.23
OVCAR-5	5.52	51.0	5.99	51.6	5.80	NT	5.65	NT	NT	NT	NT	5.81	20.9	5.57
OVCAR-8	6.73	54.1	6.67	30.8	NT	7.79	NT	NT	NT	NT	NT	NT	>50.0	10.0
NCI/ADR-RES	6.18	56.3	6.84	10.4	NT	7.30	6.73	NT	NT	NT	NT	>100	4.77	6.54
SK-OV-3	6.64	59.2	35.2	56.2	36.9	6.28	6.46	NT	5.82	NT	6.03	5.91	30.5	5.85
MG_MID	6.24	45.55	11.42	41.93	12.26	6.78	6.19	-	5.82	6.69	6.03	29.43	19.68	6.49
Renal Cancer														
786-0	6.19	12.7	5.85	40.9	6.18	NT	NT	NT	NT	NT	NT	NT	2.97	7.04
A498	33.0	86.7	6.63	52.7	8.00	7.08	6.93	5.81	6.04	5.60	57.6	5.74	27.7	5.44
ACHN	5.61	38.3	5.54	33.0	5.55	NT	5.62	NT	NT	NT	5.58	5.50	17.0	5.60
CAKI-1	5.40	53.6	6.43	39.3	5.45	5.73	5.53	5.60	5.71	NT	5.46	5.81	17.9	5.34
RXF 393	6.04	48.6	6.79	9.35	5.69	6.33	5.98	NT	NT	NT	5.59	6.05	3.07	5.81

SN12C	5.69	45.6	5.87	41.7	6.10	6.12	6.45	NT	NT	NT	NT	NT	3.34	6.03
TK-10	6.96	49.9	5.97	27.3	6.91	6.32	6.30	5.89	NT	NT	6.27	6.85	24.6	6.37
UO-31	5.74	62.1	5.79	42.1	5.77	5.52	5.42	NT	NT	NT	5.58	5.59	9.64	5.24
MG_MID	9.33	49.68	6.11	35.79	6.21	6.18	6.03	5.76	5.87	5.60	14.35	5.92	13.28	5.86
Prostate cancer														
PC-3	5.74	7.47	5.66	38.7	6.25	5.68	5.78	NT	NT	NT	NT	5.82	26.0	6.33
DU-145	5.58	9.61	5.75	13.8	6.00	6.26	6.38	NT	NT	NT	6.35	6.39	11.3	5.78
MG_MID	5.66	8.54	5.70	26.25	6.12	5.97	6.08	-	-	-	6.35	6.10	18.65	12.11
Breast cancer														
MCF7	5.94	22.7	7.28	46.7	6.30	6.22	NT	NT	NT	NT	NT	NT	NT	7.23
MDA-MB-31/ATCC	5.80	45.6	6.43	59.2	6.17	6.20	6.69	NT	NT	NT	NT	NT	3.04	6.02
HS 578T	31.0	77.4	32.6	71.2	2.44	15.9	9.11	NT	NT	NT	9.14	9.37	5.00	9.20
BT-549	NT	59.2	NT	39.0	NT	NT	5.96	NT	NT	NT	NT	NT	23.3	5.78
T-47D	7.96	48.6	9.66	59.5	7.99	7.00	NT	NT	NT	NT	NT	6.85	>50.0	NT
MDA-MB-468	6.88	7.81	6.98	55.1	7.39	6.60	6.50	6.01	NT	6.87	NT	6.51	3.36	6.47
MG_MID	11.52	43.55	12.59	55.12	6.06	8.38	7.06	6.01	-	6.87	9.14	7.57	16.94	6.94
MG_MID_{60}	7.16	32.0	7.64	41.94	17.17	6.51	6.43	5.62	5.84	6.52	18.9	9.35	16.97	

[a]LC_{50} is a parameter of cytotoxicity and reflects the molar concentration needed to kill 50% of the cells.
